# Machine learning-based in-silico analysis identifies signatures of lysyl oxidases for prognostic and therapeutic response prediction in cancer

**DOI:** 10.1186/s12964-025-02176-1

**Published:** 2025-04-05

**Authors:** Qingyu Xu, Ling Ma, Alexander Streuer, Eva Altrock, Nanni Schmitt, Felicitas Rapp, Alessa Klär, Verena Nowak, Julia Obländer, Nadine Weimer, Iris Palme, Melda Göl, Hong-hu Zhu, Wolf-Karsten Hofmann, Daniel Nowak, Vladimir Riabov

**Affiliations:** 1 Department of Hematology and Oncology, Medical Faculty Mannheim, Heidelberg University, Mannheim, 68169 Germany; 2 Department of Hematology, Beijing Chao-Yang Hospital, Capital Medical University, Beijing, China; 3 Chinese Institutes for Medical Research, Beijing, China

**Keywords:** Machine learning, Pan cancer, Lysyl oxidases, Response prediction, Prognostic model

## Abstract

**Background:**

Lysyl oxidases (LOX/LOXL1-4) are crucial for cancer progression, yet their transcriptional regulation, potential therapeutic targeting, prognostic value and involvement in immune regulation remain poorly understood. This study comprehensively evaluates LOX/LOXL expression in cancer and highlights cancer types where targeting these enzymes and developing LOX/LOXL-based prognostic models could have significant clinical relevance.

**Methods:**

We assessed the association of LOX/LOXL expression with survival and drug sensitivity via analyzing public datasets (including bulk and single-cell RNA sequencing data of six datasets from Gene Expression Omnibus (GEO), Chinese Glioma Genome Atlas (CGGA) and Cancer Genome Atlas Program (TCGA)). We performed comprehensive machine learning-based bioinformatics analyses, including unsupervised consensus clustering, a total of 10 machine-learning algorithms for prognostic prediction and the Connectivity map tool for drug sensitivity prediction.

**Results:**

The clinical significance of the LOX/LOXL family was evaluated across 33 cancer types. Overexpression of LOX/LOXL showed a strong correlation with tumor progression and poor survival, particularly in glioma. Therefore, we developed a novel prognostic model for glioma by integrating LOX/LOXL expression and its co-expressed genes. This model was highly predictive for overall survival in glioma patients, indicating significant clinical utility in prognostic assessment. Furthermore, our analysis uncovered a distinct LOXL2-overexpressing malignant cell population in recurrent glioma, characterized by activation of collagen, laminin, and semaphorin-3 pathways, along with enhanced epithelial-mesenchymal transition. Apart from glioma, our data revealed the role of LOXL3 overexpression in macrophages and in predicting the response to immune checkpoint blockade in bladder and renal cancers. Given the pro-tumor role of LOX/LOXL genes in most analyzed cancers, we identified potential therapeutic compounds, such as the VEGFR inhibitor cediranib, to target pan-LOX/LOXL overexpression in cancer.

**Conclusions:**

Our study provides novel insights into the potential value of LOX/LOXL in cancer pathogenesis and treatment, and particularly its prognostic significance in glioma.

**Supplementary Information:**

The online version contains supplementary material available at 10.1186/s12964-025-02176-1.

## Introduction

Lysyl oxidases (LOX and LOXL1-4) are copper-dependent enzymes that play a key role in the remodeling of extracellular matrix (ECM) [1, 2]. Through the process of LOX/LOXL-dependent oxidative deamination, peptidyl lysine residues in collagens and elastin undergo covalent cross-linking, thereby promoting the establishment and maintenance of the structural integrity and stability of ECM [1–3]. Previous studies reported that LOX/LOXL overexpression was associated with inferior survival in squamous cell carcinomas, prostate cancer, and breast cancer [4, 5] and promoted cancer invasion and distant metastasis by remodeling ECM in colorectal and breast cancer [6–8]. Moreover, the LOX/LOXL enzyme family has been implicated in signaling pathways regulating tumor progression, such as pathways of vascular endothelial growth factor (VEGF), transforming growth factor β (TGF-β), NF-κB, PI3K/AKT, and MAPK [9]. Since LOX/LOXL family members have been associated with inferior survival, efforts have been made to use LOX/LOXL expression as a predictive biomarker for survival in cancer patients. In glioma, higher *LOXL1* expression was associated with inferior survival, and *LOXL1*-based nomogram model showed moderate AUC values for 1- and 2-year survival [10]. Similar associations between LOX/LOXL expression and patient survival were observed in pancreatic adenocarcinoma. However, predictive models were not generated [11].

Due to the reported pro-tumor effects of LOX/LOXL enzymes in some cancers, specific small-molecule inhibitors of enzymatic activity, such as PXS-5505, have been generated and showed promising anti-tumor effects in pre-clinical models of pancreatic and hematological cancers [12, 13]. Based on the promising pre-clinical data, several clinical trials were initiated to investigate safety, tolerability, and efficacy of these inhibitors in myelofibrosis (NCT04676529) and hepatocellular carcinoma (NCT05152849). However, the identified non-enzymatic functions of LOX/LOXL members in cancer pathogenesis may require generation of alternative inhibitors and novel targeting approaches [14].

Considering a complex relationship between LOX/LOXL and neoplasia as well as the identified gaps of knowledge in signaling pathway interaction and cancer-specific involvement of LOX/LOXL family, this study explored the clinical significance and underlying biology of LOX/LOXL expression in cancer by incorporating multiple datasets. Our results revealed multifaceted roles of LOX/LOXL family in cancer progression on the level of signaling pathway activation, tumor microenvironment regulation, tumor cell invasiveness, and clinical parameters of tumor progression in multiple types of cancer. These results prompted us to extend and improve previously reported prognostic models resulting in highly sensitive nomogram model for glioma that incorporated expression of LOX/LOXL and their co-expressed genes. Our study also identified potential approaches to therapeutically counteract the LOX/LOXL expression signature in cancer.

## Materials and methods

Routine protocols are described in detail in Supplemental Methods.

### LOX/LOXL mRNA expression analysis

The RNA sequencing (RNA-seq) data of the Cancer Genome Atlas (TCGA) and Genotype-Tissue Expression (GTEx) were downloaded from the UCSC Xena platform [15]. GTEx contains data from 54 types of healthy human tissues [16]. The tumor cell line mRNA expression matrix was obtained from the Cancer Cell Line Encyclopedia (CCLE) dataset [17]. Unsupervised consensus clustering using the Euclidean distance metric with complete linkage was performed by ComplexHeatmap for displaying the distribution pattern of LOX/LOXL expression in cell lines [18]. The mRNA expression data and clinical information of glioma patients were obtained from the Chinese Glioma Genome Atlas (CGGA) [19]. The RNA-seq dataset of diffuse large B-cell lymphoma (DLBCL) from the NCI Center for Cancer Research (NCICCR) were downloaded using the TCGAbiolinks tool [20, 21]. The RNA-seq data and clinical information of GSE176307 and IMvigor210CoreBiologies datasets for bladder cancer were downloaded from the Gene Expression Omnibus (GEO) and R package "IMvigor210CoreBiologies", respectively [22, 23]. The RNA-seq data and clinical characteristics for the clinical trial of renal cell carcinoma (NCT03141177) were directly obtained from the publication [24].

Overall, the mRNA expression data were processed uniformly by Toil to get transcripts per million (TPM) [25] or converted from fragments per kilobase per million mapped fragments (FPKM) to TPM using a previously reported method [26]. The quantification and comparison were based on Log2(TPM + 1).

### Survival analysis and construction of the prognostic model

The survival information including overall survival (OS) of TCGA was obtained from Liu, et al. [27]. The association between LOX/LOXL expression and survival in TCGA pan-cancer database was assessed by the Log-rank test. Next, genes significantly correlated with LOX/LOXL expression were identified in TCGA dataset (Spearman's r ≥ 0.7). To obtain the genes tightly co-expressed with LOX/LOXL, we focused on those co-expressed with ≥ 3 out of five LOX/LOXL genes. Finally, to establish a consensus of LOX/LOXL-related signatures (LOXRS, including LOX/LOXL and LOX/LOXL co-expressed genes) with high accuracy and stability for the purpose of survival prediction, we integrated 10 machine-learning algorithms covering 101 types of algorithm combinations [28]. The algorithms included stepwise Cox, random survival forest (RSF), elastic network (Enet), Lasso, Ridge, CoxBoost, supervised principal components (SuperPC), generalized boosted regression modeling (GBM), partial least squares regression for Cox (plsRcox), and survival support vector machine (survival-SVM). The rationale for selection and integration of machine-learning tools is presented in Supplementary Methods.

The procedure for generating the signatures involved the following steps: (a) The LOXRS systems were subjected to the 101 algorithm combinations to construct prognostic models using leave-one-out cross-validation (LOOCV) in TCGA, (b) Based on the final LOXRS gene panel filtered by each model, a multivariable Cox model was constructed to calculate the single-sample LOXRS score using Cox regression coefficients and feature expressions, (c) All models were further cross-validated using CGGA1 and CGGA2 cohorts, (d) The Harrell's concordance index (C-index) was calculated across all validation datasets, and the model with the highest average C-index was deemed optimal.

The final LOXRS score was established by the following equation using the Log2(TPM + 1) of *n* = 12 genes and indices calculated by the best algorithm combination:

$$LOXL\mathit1\ast0.2943642\;+\;LOXL\mathit2\ast0.1144352\;+\;LOXL\mathit4\ast0.1634691\;+\;CASP\mathit4\ast0.3219774\;+\;CLIC\mathit1\ast0.1709626\;+\;COL\mathit1A1\ast(-0.1321534)\;+\;EMP\mathit3\ast0.1853599\;+\;GLA\ast0.63907\;+\;MSN\ast0.4161365\;+\;MYL12A\ast(-0.2278614)\;+\;PYGL\ast(-0.3132768)\;+\;VASP\ast(-0.4621118)\;+\;3.461644079$$

The Nomogram model was further established using the multivariable Cox analysis (R package "rms" v.6.3-0), including LOXRS score, WHO grade, age and IDH mutational status of TCGA glioma. The final Cox score was established by the following equation:

$$-0.047877593\;+\;\mathrm{LOXRS}\;\mathrm{score}\ast0.833485862\;+\;\mathrm{WHO}\;\mathrm{grade}\;\mathrm{III}-\mathrm{IV}\ast0.614080855\;+\;\mathrm{Age}>40\ast0.60851696\;+\;\mathrm{mutant}\;\mathrm{IDH}\ast(-0.053501247)$$

Kaplan-Meier analysis was conducted using R packages "survival", "survminer" (v. 0.4.9) and "ggplot2" (v.3.4.2). Log-rank tests were performed to evaluate statistical significance.

### Biological pathway analysis

Single-gene differential analysis of RNA-seq was performed using the R package "DESeq2" (v.1.36.0) to obtain differentially expressed genes (DEGs) between the datasets of samples with top 30% of LOX/LOXL expression levels vs. bottom 30%. The Gene Set Enrichment Analysis (GSEA) was performed for pathway enrichment based on DEGs using the R package "clusterProfiler" (v.4.6.2) [29, 30]. The enrichment score of specific pathways was calculated by R Bioconductor package "GSVA" (Gene Set Variation Analysis, v.1.46.0).

### Single-cell RNA sequencing (scRNA-seq) analysis

Analysis of the distribution of LOX/LOXL gene expression on the single cell level in healthy tissues was done using the DISCO tool [31].

The scRNA-seq data of *n* = 7 cases with glioblastoma multiforme were obtained from GEO, including primary tumor and matched recurrent samples (GSM5319506, GSM5319507, GSM5319509, GSM5319513, GSM5319517, GSM5319518, GSM5319519, GSM5319545, GSM5319546, GSM5319547, GSM5319548, GSM5319549, GSM5319562 and GSM5319565) [32]. The scRNA-seq data of *n* = 3 cases with renal cell carcinoma were obtained from GSE121638 [33]. The scRNA-seq expression matrix was processed with the package "Seurat" (v.4.3.0) [34]. Batch effect correction was performed using the package "Harmony" (v.0.1.1) [35]. Dimensionality reduction was carried out and visualized using PCA and UMAP. We used the package "SingleR" (v.2.0.0) [36] and "CellMarker" website [37] for cluster annotation. Cluster visualization and gene expression distribution were visualized using "scRNAtoolVis" (v.0.0.5). The package "CellChat" (v.1.6.1) was employed to identify potential cell–cell communication using a curated database of receptor-ligand interactions [38]. The package "infercnv" (v.1.14.2) was used to infer CNVs with scRNA-seq data and identify malignant cells.

### Drug prediction for targeting pan-LOX/LOXL overexpression

Unsupervised consensus clustering was performed using the R package "ConsensusClusterPlus" (v.1.64.0) [39]. This analysis utilized agglomerative pam, km or kmdist clustering with a 1-Pearson correlation distance and 80% sample resampling for 10 repetitions. The optimal number of clusters was determined using the empirical cumulative distribution function plot.

Via this unsupervised consensus clustering, patients were grouped into two clusters (cluster1 and cluster2) based on LOX/LOXL mRNA expression. We next chose cancers showing uniformly increased expression of each LOX/LOXL gene by comparing cluster1 to cluster2 using Wilcoxon rank sum test (*p* < 0.05). The DEGs were identified by comparing the mRNA datasets of cluster1 to cluster2 using the R package "DESeq2" (v.1.36.0). The top 150 positively and top 150 negatively correlated genes were subjected to the Connectivity map tool (cMap) [40]. Compounds with enrichment score < -60 were regarded as the inhibitors targeting pan-LOX/LOXL overexpression.

In addition, the prediction of drug sensitivities was applied by the R package "oncoPredict" (v.0.2) [41], which utilized the half-maximal inhibitory concentration (IC50) of 198 drugs obtained from the Genomics of Drug Sensitivity in Cancer (GDSC) database [42]. Potential IC50 of 198 drugs were predicted in both TCGA and NCICCR projects. The Venn diagram was generated by the R package "VennDiagram" (v.1.7.3).

### Statistical analysis

Except for the statistical methods specifically mentioned, all statistical analyses and algorithms were implemented by R software (v.4.0.3, Vienna, Austria). The "ggplot2" package and Graphpad Prism (v.8.4.3, San Diego, CA, USA) were used for visualization. Appropriate statistical methods were automatically chosen based on the characteristics of the data format using the packages "stats" (v.4.2.1) and "car" (v.3.1-0). The Shapiro-Wilk test was used to assess the data normality. If unpaired continuous variables were approximately normally distributed within each group (*p* > 0.05), a parametric test was chosen, including Student's t test for two-group comparison and one-way ANOVA for multiple-group comparison. Otherwise, a non-parametric test was performed, including Wilcoxon rank sum test for two-group comparison and Kruskal-Wallis test with Dunn's multiple comparisons for multiple-group comparison. The Spearman correlation analysis was conducted to identify significant abundance relationships. *P*-values less than 0.05 were considered statistically significant.

## Results

### LOX/LOXL expression is dysregulated in tumor cells

**Fig. 1:**
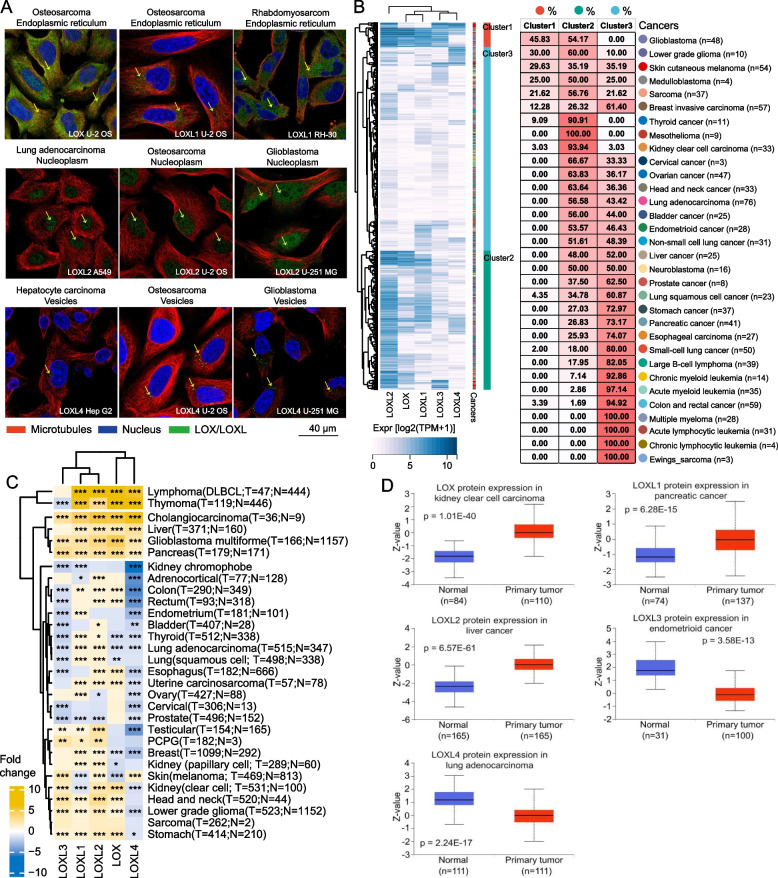
LOX/LOXL genes are expressed in tumor and stroma cells. **A** Intracellular protein expression of LOX/LOXL in cell lines of osteosarcoma, rhabdomyosarcoma, lung adenocarcinoma, glioblastoma and hepatocellular carcinoma. Scanning images of immunofluorescence staining for LOX/LOXL proteins were obtained from the Human Protein Atlas database. LOX/LOXL-positive areas are indicated in green colour. **B** Left panel: LOX/LOXL mRNA expression pattern is shown in *n* = 946 types of human tumor cell lines from 32 cancer types based on CCLE. The unsupervised consensus clustering of LOX/LOXL expression reveals three distinct clusters in the right box. Unsupervised clustering used Euclidean distance metric with complete linkage. In the heatmap each column represents a gene and each row is a type of cell line. Blue colour indicates relatively higher expression. The expression data were presented as Log2(TPM + 1). Three clusters are shown: cluster1 (red), cluster2 (green), cluster3 (blue). Right panel: distribution of *n* = 946 cell lines in the three clusters. Each row is a cancer type, and each column represents a cluster. The number and the red colour intensity in each box show the percentage of cell lines classified in the corresponding cluster. **C** Dysregulation of LOX/LOXL mRNA expression in pan cancer. LOX/LOXL gene expression was compared between tumor (TCGA) and healthy tissues (TCGA + GTEx) in pan cancer. Wilcoxon rank sum test was performed. The FC comparing median LOX/LOXL gene expression in tumor to healthy tissues are shown in the heatmap. In case of increase of expression in tumor compared to healthy controls, positive FC values were assigned. *p < 0.05, **p < 0.01, ***p < 0.001, ****p < 0.0001. **D** Dysregulation of LOX/LOXL protein expression in pan cancer. LOX and LOXL1-4 protein expression was compared between healthy tissues and tumor using the UALCAN portal. *P*-value < 0.05 is considered statistically significant. Abbreviations: DLBCL, diffuse large B-cell lymphoma; FC, fold changes; PCPG, paraganglioma & pheochromocytoma

LOX, LOXL1, LOXL2, and LOXL4 proteins were initially observed in different intracellular compartments of tumor cells, including endoplasmic reticulum, nucleoplasm and vesicles in osteosarcoma, rhabdomyosarcoma, lung adenocarcinoma, glioblastoma and hepatocellular carcinoma cell lines (Fig. 1A). Subsequently, we analyzed the expression patterns of LOX/LOXL in pan cancer, including a total of *n *= 946 human cell lines representing 32 different cancer types, utilizing the data from CCLE (Fig. 1B). The unsupervised consensus clustering analysis revealed three distinct clusters of LOX/LOXL expression patterns, with cluster 1 displaying the highest expression of LOX and LOXL1-3 genes (Fig. 1B, left panel). Cluster 2 was characterized by specific overexpression of *LOXL2*, while cluster 3 demonstrated the lowest levels of LOX/LOXL expression (Fig. 1B, left panel). Examining the distribution of cancer types within these clusters, we observed that neural-related tumors, skin melanoma, and sarcoma largely located in clusters 1 and 2, whereas hematological malignancies, small-cell lung cancer and gastrointestinal tumors predominantly resided in cluster 3 (Fig. 1B, right panel). Cluster 2 additionally contained over 90% of cell lines of thyroid cancer, mesothelioma and kidney carcinoma (Fig. 1B, right panel). These findings suggest a dysregulated expression profile of LOX/LOXL that varies depending on cancer types.

We further observed heterogeneous LOX/LOXL expression patterns in tumor vs. normal tissues in TCGA, including nine tumor types (e.g., thymoma and glioma) with overexpression of ≥ 4 LOX/LOXL genes in malignant tissues (Fig. 1C). *LOXL2* was overexpressed in 24 tumor types, while *LOXL4* expression decreased in 18 cancer types. Protein expression data from the UALCAN tool confirmed altered LOX/LOXL expression in tumors (Fig. 1D and Fig. S1A). LOXL1-4 proteins were also detected in primary tumor tissues of colon, liver, breast, and lung cancers by immunohistochemistry (Fig. S1B).

### LOX/LOXL expression correlates with tumor progression and metastasis and with the activation of pro-tumor pathways

**Fig. 2:**
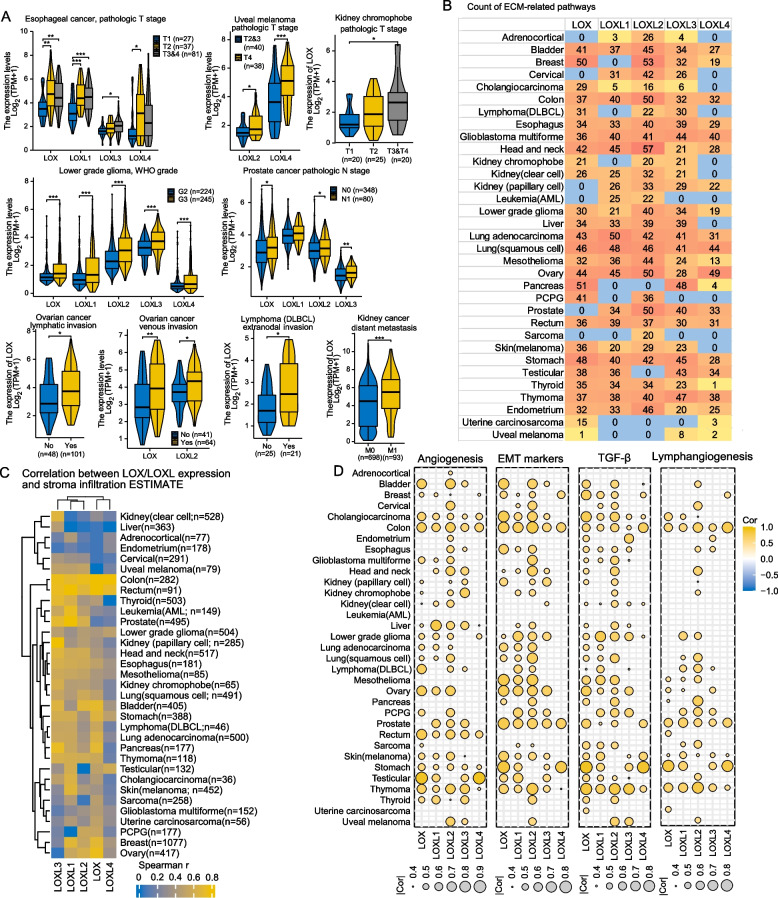
LOX/LOXL expression is correlated with tumor stage, invasion, and metastasis through multiple signalling pathways. **A** The comparison of LOX/LOXL expression based on tumor (T) and node (N) stage, invasion, and distant metastasis. All boxplots and violin plots represent the median and quartiles of the data. Appropriate statistical methods were automatically chosen based on the characteristics of the data format using the statistical R packages "stats" (v.4.2.1) and "car" (v.3.1-0). * *p* < 0.05, **p < 0.01, ***p < 0.001. **B** Count summary of activated ECM-related pathways in pan cancer. ECM-related pathways were enriched by GSEA based on LOX/LOXL overexpression. **C** Correlation between LOX/LOXL expression and stromal infiltration. The stromal infiltration was calculated by the R package "ESTIMATE" (v.1.0.13) and correlated with LOX/LOXL expression by the Spearman correlation analysis. Bright yellow color indicated higher Spearman's r values. **D** Correlation between LOX/LOXL expression and enrichment score of biological pathways including angiogenesis, EMT, TGF-β and lymphangiogenesis. The enrichment score was calculated by the R package "GSVA" (Gene Set Variation Analysis, v.1.46.0) and correlated with LOX/LOXL expression by the Spearman correlation analysis. The size of circles indicated Spearman's r values. Only results with *p* < 0.05 were displayed. For panels **A** and **D**, *p*‐value < 0.05 is considered statistically significant. Abbreviations: AML, acute myeloid leukemia

Our study found the relationship between LOX/LOXL overexpression and advanced tumor stage, lymphatic invasion, vascular invasion, and distant metastasis in different tumor types (Fig. 2A, Fig. S2). To further understand the underlying mechanisms, we conducted pathway enrichment analysis.

GSEA identified 33 activated pathways in pan cancer, with ECM remodeling pathways being the most significantly enriched (Fig. 2B and Fig. S3). This finding was further validated by a positive correlation between LOX/LOXL expression and GSVA scores of ECM remodeling pathways, such as collagen formation (Fig. S4). The ESTIMATE stromal score and cancer-associated fibroblast infiltration were also positively correlated with LOX/LOXL overexpression (Fig. 2C and Fig. S5). We additionally found highly enriched activated pathways of vascular function, cell movement, cell migration and invasion, and integrin signaling (Fig. S6). LOX/LOXL expression was positively linked with angiogenesis, epithelial-mesenchymal transition (EMT), TGF-β, and lymphangiogenesis (Fig. 2D).

LOX/LOXL^high^ cohort showed enrichment of inflammation- and carcinogenesis-related pathways, including Wnt, PI3K, JAK/STAT, and MET pathways. (Fig. S7). In addition, LOX/LOXL overexpression in 12 types of tumors led to the enrichment of cell cycle and chromosome pathways (Figs. S8A and B). LOX/LOXL^high^ groups had higher levels of loss of heterozygosity (LOH) score, indicating the role in disrupting genome integrity, particularly in glioma (Fig. S8C).

Taken together, our findings suggest the critical role of LOX/LOXL family in regulating tumorigenesis, invasion, and metastasis in the clinical setting through the activation of various signaling pathways.

### Dysregulated LOX/LOXL expression affects prognosis in cancer, especially in glioma

Previous studies investigated the prognostic significance of LOX/LOXL mRNA expression in various cancers, such as squamous cell carcinomas, hepatocellular carcinoma, pancreatic cancer, and glioma [4, 13, 43]. We further performed survival analyses in pan cancer and observed significant associations between LOX/LOXL expression and prognosis in *n* = 24 types of tumor, with patients diagnosed with glioma (lower grade glioma and glioblastoma multiforme) experiencing the most unfavorable outcomes (Table S1).

**Fig. 3:**
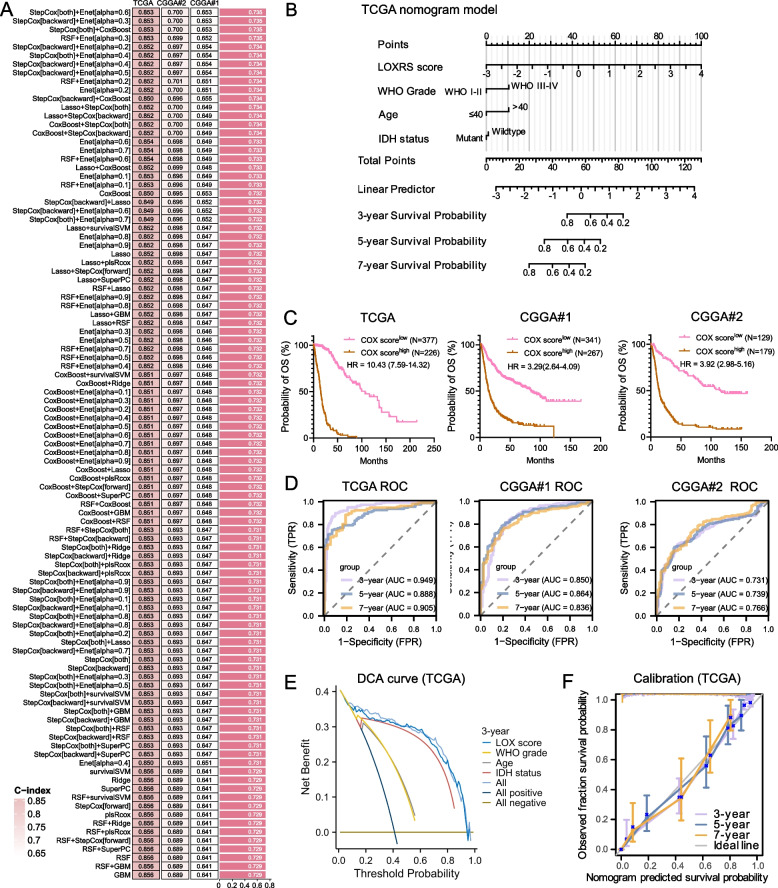
Dysregulated LOX/LOXL mRNA expression affects prognosis of patients with glioma. **A** The C-indexes of 101 prediction models via the LOOCV framework followed by the multivariable Cox model was calculated across TCGA and two CGGA datasets. **B** Prognostic nomogram model based on the LOX score and three clinical features of glioma in the TCGA cohort. The predicted OS probability is calculated by summarizing points from each of parameter. **C** OS comparison between high and low Cox score in TCGA and CGGA cohorts. Kaplan-Meier analysis and log-rank was used for survival comparison. Log-rank test was performed to evaluate the statistical significance. *P*‐value < 0.05 is considered statistically significant. **D** ROC analysis of LOX score prediction capability for 3-, 5-, and 7-year OS in TCGA and CGGA cohorts. **E** DCA evaluation of the clinical practicability of the Cox model and calculation and comparison of the clinical benefit rate of each variable. **F** Calibration curve analysis for validation of the accuracy and stability of Cox score. Abbreviations: AUC: Area Under the Curve

To more accurately predict OS in glioma, we identified the genes co-expressed with LOX/LOXL (Spearman's r ≥ 0.7, Fig. S9A). These 21 genes, along with LOX/LOXL genes, were then subjected to our machine learning-based integrative approach to generate LOXRS. Subsequently, in TCGA-glioma dataset we fitted 101 prediction models via the LOOCV framework and calculated the C-index of each model across all validation datasets (two CGGA cohorts). Notably, the top three models, "StepCox[both] + Enet[alpha = 0.6]", "StepCox[backward] + Enet[alpha = 0.3]", and "StepCox[both] + CoxBoost", showed the highest average of C-indices (Table S2) covering TCGA and the two CGGA cohorts (Fig. 3A), and resulted in inclusion of *n* = 12 out of the initial 21 genes into the final LOXRS (*LOX*, *LOXL2*, *LOXL4*, *CASP4*, *CLIC1*, *COL1A1*, *EMP3*, *GLA*, *MSN*, *MYL12A*, *PYGL* and *VASP*). The LOXRS score was further calculated based on the expression of these genes in a multivariable Cox model.

To more accurately predict OS probability, a nomogram model was developed through the Cox analysis incorporating the LOXRS score and three prevalent clinical features (age, WHO grade, and IDH mutation status) (Fig. 3B). The concordance index of this model was as high as 0.870 (0.859-0.882). As exemplified in Table S3 the predicted OS probability of *n *= 7 TCGA and CGGA cases were consistent with their real survival.

Utilizing the coefficients of four variables in the nomogram model and the Cox analysis, a Cox risk score was computed. By taking the optimal threshold (1.064) of the Cox score all TCGA patients with high score showed significantly decreased OS, which was validated by the two CGGA cohorts (Fig. 3C, *p *< 0.0001). Receiver Operating Characteristic (ROC) validated the predictive ability of this model, showing Area Under the Curve (AUC) values of > 0.73 for 3-, 5-, and 7-year survival prediction (Fig. 3D). The decision curve analysis (DCA) showed that the clinical benefit rate of LOXRS score alone was comparable to the combination of four variables, especially for 3-year OS (Fig. 3E, Fig. S9B). Calibration curves showed the consistence between predicted values and observed values for 3-, 5-, and 7-year OS in TCGA (Fig. 3F).

We conclude that dysregulated LOX/LOXL expression affects prognosis in pan cancer, especially in glioma.

### ScRNA-seq reveals a distinct role of LOXL2 in recurrent IDH wild-type GBM

**Fig. 4:**
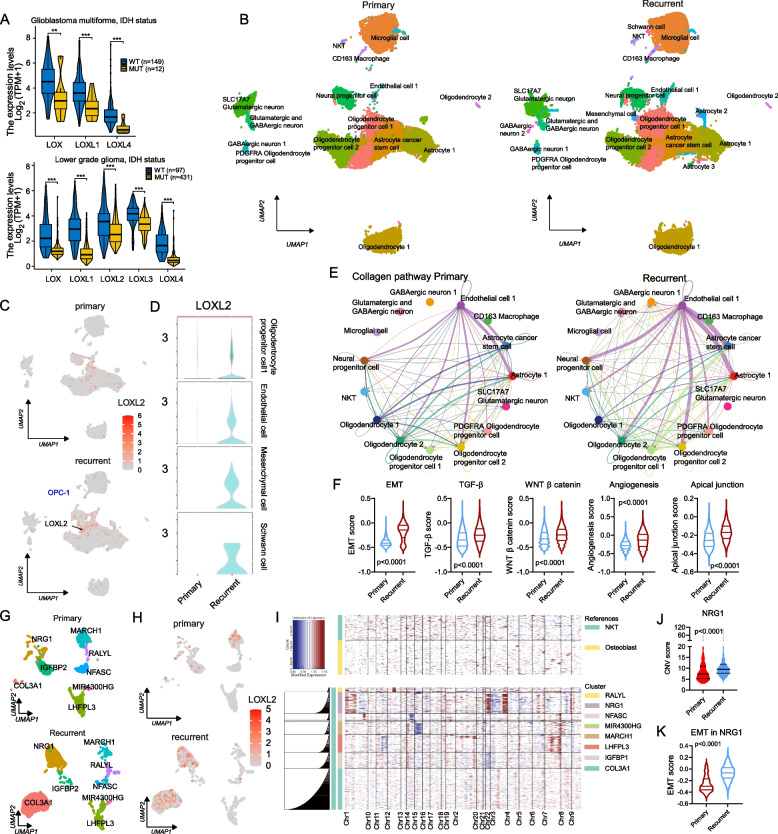
scRNA-seq reveals a distinct role of *LOXL2* in recurrent IDH wild-type glioblastoma multiforme. **A** The comparison of LOX/LOXL mRNA expression between IDH wild-type and mutant glioma. All boxplots and violin plots represent the median and quartiles of the data. **B** UMAP represented the landscape of IDH wild-type glioblastoma multiforme from primary and patient-matched recurrent tissues (*n* = 7 patients). *N* = 20 clusters were identified. **C** Distribution of *LOXL2* mRNA expression in primary and recurrent status. **D** Violin plots visualization of *LOXL2* mRNA expression in clusters of OPC-1, endothelial cell 1, mesenchymal cell, and Schwann cell. **E** The comparison of cell-cell collagen pathway communication network between primary and recurrent cell populations. In the circle plots, edge colours are consistent with the sources as signal sender of pathways, and edge widths are proportional to the interaction strength. Thicker edge line indicates a stronger signal. **F** Pathway enrichment score comparison between primary and recurrent OPC-1 population. Scores were calculated using the R package "GSVA" (v.1.46.0), including pathways involving EMT, TGF-β, WNT/β-catenin signalling, angiogenesis and apical junction. **G** Using UMAP dimensionality reduction, OPC-1 cells were further divided into eight sub-clusters. **H** Distribution of *LOXL2* mRNA expression in the *n* = 8 sub-clusters of both primary and recurrent OPC-1 population. **I** Malignant cells in sub-clusters of OPC-1 population were identified by inferring large-scale CNVs with the total NKT cells and mesenchymal cells as references. **J** CNV score comparison between primary and recurrent NRG1 cluster. **K** EMT enrichment score comparison between primary and recurrent NRG1 cluster from OPC-1 population. For panels **A**, **F**, **J** and **K**, Wilcoxon rank sum test was performed for statistical analyses. *P*‐value < 0.05 is considered statistically significant. **p < 0.01, ***p < 0.001. Abbreviations: NKT, natural killer T cell

Given the significant correlation between LOX/LOXL overexpression and prognosis, WHO grade, and IDH mutational status in glioma (Fig. 4A), scRNA-seq was employed to compare LOX/LOXL expression in primary and patient-matched recurrent tumor tissues from *n* = 7 cases with IDH wild-type glioblastoma multiforme. We identified 20 distinct cellular clusters in an integrated dataset of 47,485 cells (Fig. 4B and Fig. S10). Recurrent samples exhibited additional clusters including astrocytes, mesenchymal cells, GABAergic neurons and Schwann cells compared to primary tumors (Fig. 4B and Fig. S10). Endothelial cells, mesenchymal cells, Schwann cells and oligodendrocyte progenitor cells (OPC-1) overexpressed *LOXL2* in recurrent samples (Figs. 4C and D).

The "CellChat" tool [38] identified increased cellular interaction in recurrent tumor via ECM (collagen, laminin) and semaphorin (SEMA3) networks (Fig. 4E and Fig. S11), especially shown in populations of endothelial cells, OPC-1, and oligodendrocytes. Both OPC-1 and endothelial cells were prominent sources of signals in recurrence compared to primary status (Fig. S12). Next, we identified underlying biological diversities between recurrent and primary tumors by GSVA scores of EMT, TGF-β, WNT/β-catenin, angiogenesis, and apical junction in recurrent OPC-1 (Fig. 4F). KEGG and GSEA also indicated that in recurrent stage EMT-related pathways were activated (Fig. S13).

To deeper characterize OPC-1 cluster, we conducted sub-clustering and identified eight distinct populations, which were named by the most overexpressing genes (Fig. 4G). *LOXL2* gene exhibited extensive expression in *COL3A1* and *NRG1* clusters (Fig. 4H). CNV analysis revealed pronounced CNV changes in *NRG1* cluster, but not in *COL3A1* cluster as compared to NKT and mesenchymal cell controls (Fig. 4I). Moreover, CNV burden in *NRG1* cluster significantly increased in recurrent stage compared to primary tumors (Fig. 4J) and showed higher EMT score (Fig. 4K), suggesting that *NRG1* cluster represented a distinct *LOXL2-*overexpressing malignant population.

Overall, our findings indicate that recurrent IDH wild-type glioblastoma multiforme is characterized by the appearance of *LOXL2*-overexpressing cell population associated with EMT and malignant phenotype.

### Upstream regulation of LOX/LOXL family by transcription factors and miRNA

The complex roles of the LOX/LOXL family in pan cancer prompted us to explore the upstream regulation of these genes. We firstly utilized the genetic perturbation similarity analysis database (GPSAdb) to explore the upstream genes regulating LOX/LOXL expression, using 3,048 gene knockdown/knockout RNA-seq datasets [44]. This analysis revealed 23 genes regulating LOX/LOXL expression, which significantly associated with LOX/LOXL expression in pan cancer (all *p* < 0.05, Fig. S14). Notably, ten of these genes were transcription factors, including *PRRX1*, *ETS1*, *KLF6*, *RUNX1*, *CBFB*, *TCF4*, *NFE2L2*, *PU1*, *STAT3*, and *SUPT6H*.

We next interrogated miRNA regulation of LOX/LOXL expression using "GSCAlite" [45]. The visNetwork was used to generate miRNA regulation networks. *N* = 42 miRNAs were found to negatively regulate *LOX*, *LOXL1*, *LOXL2* and *LOXL4* expression (*p* < 0.05; Fig. S15). In particular, hsa-miR-148b-3p strongly down-regulated *LOXL1* expression, whereas hsa-miR-29c-3p, hsa-miR-29a-3p and hsa-miR-26b-5p remarkably down-regulated *LOXL2* expression.

### Prediction of potential compounds targeting pan-LOX/LOXL overexpression by cMap and machine-learning

**Fig. 5:**
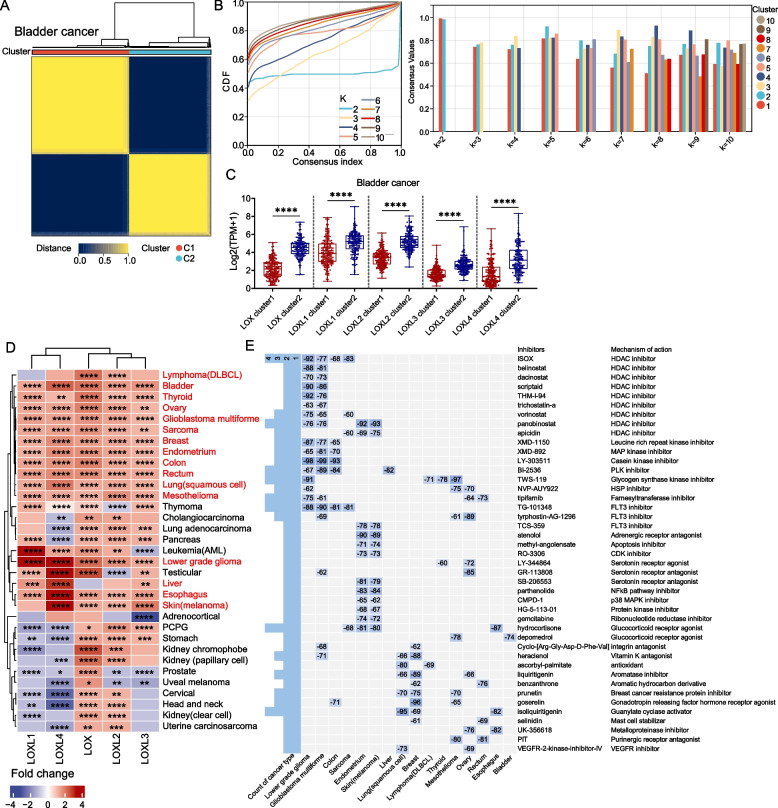
Prediction of potential compounds targeting pan-LOX/LOXL overexpression by cMap. **A** TCGA patients were grouped into two clusters based on pan-LOX/LOXL expression pattern by using "ConsensusClusterPlus" (v.1.64.0). Bladder cancer is shown as an example. **B** The left panel for empirical CDF plots displays consensus distributions for each k (k means cluster) after clustering of bladder cancer patients. K = 2 showed a maximum stability. The right panel for cluster-consensus displays similarity of clusters in each k value. Strong similarity of clusters was achieved at k = 2. **C** Based on results obtained from panels **A** and **B**, patients were grouped into cluster1 and cluster2. Expression of each LOX/LOXL gene was higher in cluster2 as compared to cluster1. **D** All TCGA patients were grouped in two clusters based on pan-LOX/LOXL expression pattern as described in panels **A-C**. The FC comparing median expression of LOX/LOXL between cluster2 and cluster1 are shown in the heatmap. In case of expression increase in cluster2 compared to cluster1, positive FC values were assigned. 16 types of cancer displayed statistically significant pan-LOX/LOXL overexpression in cluster2 compared to cluster1 (labelled as red fonts). **E** cMap analysis revealed distinct compounds targeting pan-LOX/LOXL overexpression in 16 types of malignancies identified in panel **D**. Only compounds found in ≥ 2 tumor types and with values ranged from -99 to -60 were displayed in the heatmap. Lower values shown in each box indicated stronger inhibiting effects. For panels **C** and **D**, Wilcoxon rank sum test was performed for statistical analyses. *P*‐value < 0.05 is considered statistically significant. *p < 0.05, **p < 0.01, ***p < 0.001, ****p < 0.0001. Abbreviations: CDF, cumulative distribution function; CDK, cyclin-dependent kinase; FLT3, fms-like tyrosine kinase 3; HDAC, histone deacetylase; HSP, heat shock protein; MAPK, mitogen-activated protein kinase; MAP, mitogen-activated protein; PLK, Polo-like kinase; VEGFR, vascular endothelial growth factor receptor

Given the consistent activation of pathways associated with tumor progression in LOX/LOXL-overexpressing tumors, we performed unsupervised consensus clustering analysis using "ConsensusClusterPlus" to identify patients with consistently altered expression patterns of these genes. Totally, in 16 tumor types patients were classified into two distinct groups (cluster 1 and 2), showing significant overexpression of each LOX/LOXL gene in cluster2 as compared to cluster1 (Figs. 5A-C, bladder cancer as an example, Fig. 5D).

To identify potential compounds that may counteract pathways involving LOX/LOXL overexpression in the 16 malignancies, we utilized the cMap method, which uses genetic perturbation similarity analysis to establish connections between genes and chemicals [40]. We finally identified 118 potential inhibitors. Figure 5E illustrates the compounds predicted to be effective in ≥ 2 tumor types.

**Fig. 6:**
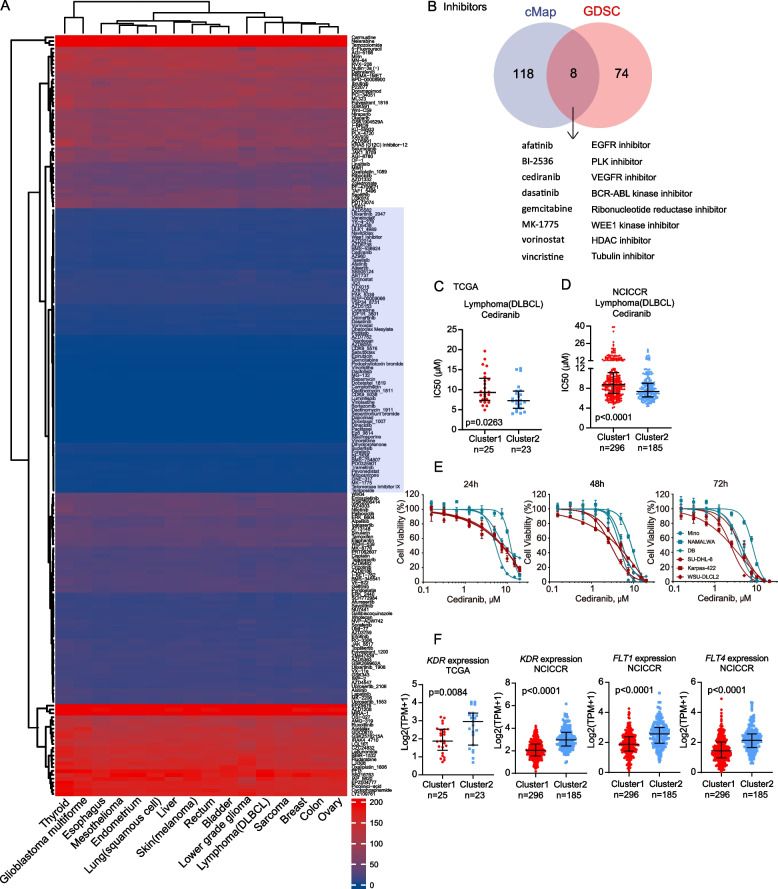
Prediction of drug sensitivity targeting pan-LOX/LOXL overexpression by machine-learning. **A** The heatmap summarized the predicted drugs IC50 values (µM) of each TCGA sample in the 16 types of tumor identified in Fig. 6D. The predicted IC50 values were calculated by the machine-learning based R package "oncoPredict" (v.0.2). In the heatmap each column represents a type of cancer and each row is a drug. A total of *n* = 198 drugs was included in this analysis. Stronger blue color indicates lower IC50 value. The unsupervised consensus clustering in the heatmap reveals a distinct cluster marked by a blue color in the column of drug annotation, showing *n* = 74 compounds with relatively lower IC50 values (0.006–16.305 µM). Unsupervised clustering used Euclidean distance metric with complete linkage. **B** Venn diagram showing inhibitors of pan-LOX/LOXL overexpression transcription signature shared by both cMap and "oncoPredict" analysis. **C-D** IC50 values of cediranib were compared between pan-LOX/LOXL high and low group in TCGA DLBCL (**C**) and NCICCR DLBCL cohort (**D**). **E** LOX/LOXL-high cell lines (SU-DHL-6, Karpas-422, WSU-DLBCL2) and -low cells lines (Mino, NAMALWA, DB) of lymphoma were treated with the indicated concentrations of cediranib, and cell viability was assessed using CellTiter-Glo assay; mean ± SD of 5 cell culture replicates; **F** Expression of *KDR* (VEGFR2), *FLT1* (VEGFR1), and *FLT4* (VEGFR3) genes were compared between pan-LOX/LOXL high and low group in TCGA DLBCL and NCICCR DLBCL cohort; For panel **C**, **D**, and **F**, Wilcoxon rank sum test was performed for statistical analysis. *P*‐value < 0.05 is considered statistically significant. Abbreviations: EGFR, epidermal growth factor receptor; VEGFR: vascular endothelial growth factor receptor

Furthermore, to forecast drug sensitivity in the 16 malignancies, we utilized the "oncoPredict" package, which utilizes ridge regression analysis and the GDSC database containing information on 198 drugs. Unsupervised consensus clustering identified 98 drugs predicted to act at lower doses (0.006–16.305 µM, Fig. 6A). The Venn diagram analysis revealed eight overlapped predicted drugs between cMap and oncoPredict, including afatinib, BI-2536, cediranib, dasatinib, gemcitabine, MK-1775, vorinostat and vincristine (Fig. 6B).

We finally compared sensitivity of the selected 16 tumor types to the eight overlapped drugs using IC50 values calculated by "oncoPredict". When stratified into pan-LOX/LOXL^high^ and LOX/LOXL^low^ groups (Fig. 5D), LOX/LOXL^high^ patients with DLBCL, skin melanoma and thyroid cancer exhibited higher sensitivity to cediranib (pan-VEGFR inhibitor, Fig. 6C), gemcitabine (Fig. S16A) and dasatinib (Fig. S16B), respectively. The sensitivity to cediranib was subsequently confirmed in a larger cohort of n = 481 DLBCL patients (Fig. 6D). As a confirmation of in-silico data, n = 3 types of LOX/LOXL^high^ lymphoma cell lines showed significantly reduced viability compared to n =3 types of LOX/LOXL^low^ cell lines after cediranib treatment (Fig. 6E, Fig. S16C, Table S4). Consistent upregulation of all three VEGFRs (*KDR*/VEGFR2, *FLT1*/VEGFR1, and *FLT4*/VEGFR3) and vascular pathway was observed in LOX/LOXL^high^ DLBCL cohorts (Fig. 6F, Fig. S16D). Nevertheless, the lack of RNA-seq data in extensive cohorts of skin melanoma and thyroid cancer precluded the validation of the results for gemcitabine and dasatinib.

### *LOXL3* expression correlates with immune infiltration and may predict response to immune checkpoint blockade (ICB)

**Fig. 7:**
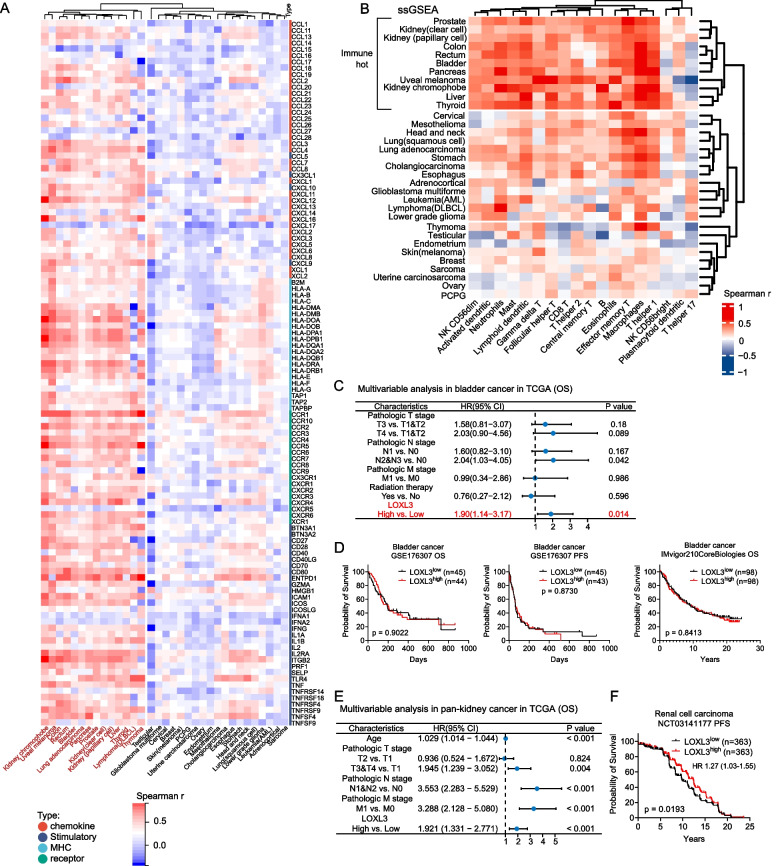
*LOXL3* mRNA expression correlates with immune infiltration and potentially predict response to ICB therapy (**A**) Heatmap of the Spearman correlation coefficient between *LOXL3* expression and immunomodulators including chemokines, immune stimulatory factors, MHC molecules and receptors in 33 malignancies. The unsupervised consensus clustering in the heatmap reveals a distinct cluster showing 14 malignancies displaying positive correlation. **B** Heatmap of the Spearman correlation coefficient between *LOXL3* expression and the infiltration abundance of *n* = 18 immune cell types in 33 malignancies. The infiltration abundance was calculated by ssGSEA. The unsupervised consensus clustering in the heatmap reveals a distinct cluster showing *n* = 11 malignancies displaying positive correlation, regarded as immunologically hot cancers. In panels **A** and **B**, unsupervised clustering of Spearman's r used Euclidean distance metric with complete linkage. Red colour indicates positive correlation, and blue colour indicates negative correlation. **C** Multivariable analysis of *LOXL3* expression and clinical characteristics as risk factors for inferior OS in TCGA bladder cancer without ICB treatment. **D** OS and PFS comparison between high and low *LOXL3* expression in two validation cohorts (GSE176307 and IMvigor210CoreBiologies cohorts with bladder cancer and receiving ICB treatment). **E** Multivariable analysis of *LOXL3* expression and other clinical characteristics as risk factors for inferior OS in TCGA kidney cancer without ICB treatment. **F** PFS comparison between high and low *LOXL3* expressed patients in the external validation cohort of renal cell carcinoma receiving ICB treatment (NCT03141177). For panels **C-F**, patients were grouped by the median level of *LOXL3* expression level. For panels **D** and **F**, Kaplan-Meier analysis and Log-rank test were used for survival comparison. *P*‐value < 0.05 is considered statistically significant. Abbreviations: HR, hazard ratio; N, node stage; M, metastasis; T, tumor stage

GSEA of LOX/LOXL^high^ vs. LOX/LOXL^low^ tumors identified enrichment of immune cell (especially T cells) associated pathways specifically in *LOXL3*-overexpressing tumors (Fig. S17). *LOXL3* positively correlated with gene expression of chemokines, immune stimulatory factors, MHC molecules and receptors in 14 malignancies (Fig. 7A). Of these malignancies, we identified 10 malignancies as immunologically hot cancers (such as kidney and bladder cancer) by assessing the infiltration abundance using four types of algorithms including ssGSEA, QUANTIseq, MCPcounter, and ImmunCellAI (Fig. 7B, Fig. S18). *LOXL3* expression in these tumors showed high positive correlation with immune infiltration, which was validated by ESTIMATE immune scores (Fig. S19).

Previous research indicated that immunologically hot tumors may be suitable candidates for ICB. Our study reveaed that *LOXL3* overexpression was an independent risk factor of OS in TCGA bladder cancer (*n* = 397) not receiving ICB (median age: 68 (34-90) years old) (Fig. 7C). However, the inferior outcome associated with *LOXL3* overexpression was not observed in two large ICB-treated cohorts with bladder cancer (GSE176307 [median age: 70 (41-89) years old; age-matched with TCGA, *p* = 0.4177] and IMvigor210CoreBiologies; KM analysis: *p* > 0.8; Fig. 7D). Moreover, although *LOXL3* overexpression was related to worse prognosis in TCGA kidney cancer not receiving ICB (*n* = 845; Fig. 7E), in a large ICB-treated cohort *LOXL3* overexpression was significantly associated with improved PFS (NCT03141177; *p* = 0.0193; Fig. 7F).

To further explore the functions of *LOXL3 i*n kidney cancer, we performed scRNA-seq re-analyses in two independent datasets (GSE121638 and data from Kevin Bi et al., Cancer Cell 2021) [33, 46]. *LOXL3* was predominantly expressed in macrophages in both datasets (Fig. S20, Fig. S21A-B). Based on GSE121638, *LOXL3* ^high^ macrophages exhibited activated immune-related pathways, including antigen processing/presentation and IFN-γ response (Fig. S21C), suggesting a potential role in modulating tumor microenvironment in kidney cancer.

Overall, *LOXL3* overexpression may influence tumor immune microenvironment and potentially predict the response to ICB in renal cell carcinoma and bladder cancer.

## Discussion

The predictive value of pan-LOX/LOXL mRNA expression in prognosis and therapeutic response in pan cancer remains uncertain. In our study data from TCGA and GEO databases were used to study LOX/LOXL genes in over 9000 samples from 33 cancer types. This analysis identified the crucial impact of LOX/LOXL overexpression on disease progression and prognosis in pan cancer, particularly in glioma. Importantly, although prognostic significance of *LOXL2* in glioma was previously reported and one study reported prognostic significance of *LOXL1* expression in glioma [10, 47], integrated LOX/LOXL predictive score reported in our study achieved much higher sensitivity in predicting 3-, 5- and 7-year survival in glioma patients. Our findings demonstrate that a prognostic model incorporating LOX/LOXL expression can effectively stratify patient risk in glioma, providing valuable predictive value.

Consistent with previous research [6–8], our study found a strong link between LOX/LOXL overexpression and cancer progression, such as metastasis and invasion, suggesing the crucial role of LOX/LOXL in driving aggressive cancer behavior and spreading to surrounding and distant sites. The observed association between LOX/LOXL and multiple pathways identified in this study further supports the complex interplay between LOX/LOXL and tumor progression [9]. Our analysis of scRNA datasets from primary and recurrent IDH wild-type glioblastoma multiforme showed an increase in stromal and cancer cell subpopulations with *LOXL2* overexpression in recurrent tumors. These subpopulations promoted intercellular communication through collagen and laminin pathways. A previous study of recurrent glioblastoma multiforme showed a shift to a mesenchymal phenotype [32]. Interestingly, our study indicated that OPC-1 with *LOXL2* overexpression had a higher EMT score compared to primary tumors, indicating the role of LOX/LOXL in promoting a more aggressive phenotype during tumor recurrence. Targeting LOX/LOXL and its pathways may help prevent glioma recurrence.

Using machine learning we predicted potential therapeutic drugs targeting LOX/LOXL overexpression. We finally identified candidates targeting DLBCL, skin melanoma and thyroid cancer, which can guide future research and development of targeted therapies for LOX/LOXL overexpression in cancer. Of note, blocking VEGF did not show significant clinical benefit in DLBCL [48], but our data suggest that cediranib, an oral small molecule blocking VEGF, might be a promising candidate targeting DLBCL with LOX/LOXL overexpression. Further research is warranted to explore the potential of *LOXL3* in improving survival for bladder and renal cancer undergoing ICB treatment. This could lead to advancements in understanding LOX/LOXL-overexpressing cancers and the development of new therapies.

However, several limitations should be noted. Further animal experiments should be conducted to validate the reliability of drug prediction involving cediranib, dasatinib, and gemcitabine. The predictive probability of *LOXL3* in immunotherapy efficacy and the corresponding mechanisms should be validated in prospective studies. It will be necessary to identify whether the *LOXL2* overexpression triggers glioma recurrence using animal models. However, the lack of functional data does not diminish the potential of LOX/LOXL as a prognostic biomarker in all types of cancer.

## Conclusions

Our study highlights the importance of LOX/LOXL as a prognostic marker and potential therapeutic target in cancer. Further research is needed to explore its role in cancer signaling pathways and potential for improving patient outcomes.

## Supplementary Information

Supplementary Material 1.

## Data Availability

All data analysed during this study are from CCLE, TCGA, CGGA, GEO, and GTEx public datasets.

## Abbreviations

AML: Acute myeloid leukemia
AUC: Area Under the Curve
CCLE: Cancer Cell Line Encyclopedia
CNVs: Copy number variants
CGGA: Chinese Glioma Genome Atlas
C-index: Concordance index
cMap: Connectivity map tool
CDF: Cumulative distribution function
DLBCL: Diffuse large B-cell lymphoma
DEGs: Differentially expressed genes
DCA: Decision curve analysis
ECM: Extracellular matrix
Enet: Elastic network
EMT: Epithelial-mesenchymal transition
FPKM: Fragments per kilobase per Million mapped fragments
GEO: Gene Expression Omnibus
GTEx: The Genotype-Tissue Expression
GSEA: Gene Set Enrichment Analysis
GDSC: Genomics of Drug Sensitivity in Cancer
ICB: Immune checkpoint blockade
LOX: Lysyl oxidase
LOXL1-4: Lysyl oxidase-like protein 1-4
LOXRS: LOX/LOXL-related signatures
LOOCV: Leave-one-out cross-validation
LOH: Loss of heterozygosity
NCICCR: NCI Center for Cancer Research
NKT: Natural killer T cell
OS: Overall survival
OPC-1: Oligodendrocyte progenitor cell 1
PFS: Progression-free survival
PCPG: Paraganglioma & pheochromocytoma
PCA: Principal Components Analysis
plsRcox: Partial least squares regression for Cox
RNA-seq: RNA sequencing
RSF: Random survival forest
ROC: Receiver Operating Characteristic
survival-SVM: Survival support vector machine
scRNA-seq: Single cell RNA sequencing
SuperPC: Supervised principal components
TGF-β: Transforming growth factor β
TCGA: The Cancer Genome Atlas Program
TPM: Transcripts per million
UMAP: Uniform Manifold Approximation and Projection
VEGFR: Vascular endothelial growth factor receptor
