# Supplementary material for: Machine learning-based in-silico analysis identifies signatures of lysyl oxidases for prognostic and therapeutic response prediction in cancer
Source: Cell Commun Signal. 2025 Apr 5;23:169. doi: 10.1186/s12964-025-02176-1 (PMC11971788; doi:10.1186/s12964-025-02176-1)
Supplement: Supplementary file 1 — Supplementary Material 1. [file 12964_2025_2176_MOESM1_ESM.pdf]

Supplementary methods

Somatic mutation analysis

After logging into the cBioPortal web (https://www.cbioportal.org/) [1, 2], we selected the “TCGA Pan Cancer Atlas Studies” in the “Query by gene” section and entered “LOX”, “LOXL1”, “LOXL2”, “LOXL3” and “LOXL4” for queries of the genetic alteration characteristics in n=10528 TCGA patients. Only the “mutations” section was selected. The mutational frequency across all TCGA tumors were analyzed in both of the “OncoPrint” and “Cancer Types Summary” modules.

Protein expression of LOX/LOXL family

Scanning images of LOX/LOXL protein expression in cell lines (immunofluorescence staining) and primary cancer specimens (immunohistochemical staining) were obtained from the Human Protein Atlas (www.proteinatlas.org) database [3].

We utilized the UALCAN portal (http://ualcan.path.uab.edu/analysis-prot.html) [4-6], an interactive web resource designed for analyzing cancer Omics data, to perform protein expression analysis on the CPTAC (Clinical Proteomic Tumor Analysis Consortium) dataset [5]. Herein, we compared the expression levels of total LOX/LOXL proteins between primary tumor and normal tissues.

Identification of co-expressed genes of LOX/LOXL gene

Spearman’s correlation coefficients between LOX/LOXL gene expression and mRNA expression of all other genes were calculated within TCGA dataset. P values were adjusted by BH method.

Survival analysis and construction of the prognostic model

The survival information including overall survival of TCGA patients were identified and obtained from Liu, et al. [7]. To examine the association between LOX/LOXL expression and TCGA pan-cancer prognosis, we employed the Cox proportional hazards regression model by using the “coxph” function from package "survival" (v.3.3.1) [8]. Log-rank test was used for statistical test to obtain prognostic significance and patients with survival lower than 30 days were excluded from this analysis.

We used R package "maxstat" (v.0.7-25) to calculate the optimal cutoff value for the Cox risk score using maximally selected rank statistics with several p-value

approximations. The minimum and maximum sample sizes were set at >25% and <75%, respectively. Subsequently, patients were stratified into score-high and -low groups based on these cutoff values, and the "survfit" function in R package "survival" was employed to examine the difference in prognosis between the two groups.

To evaluate predictive ability of Cox risk score, we utilized R package "timeROC" (v.0.4) to perform Receiver Operating Characteristic (ROC) analysis and obtain the Area Under the Curve (AUC) at three time points: 3, 5 and 7 years. AUC ≥ 70% indicated reliable predictive ability. To assess the accuracy of cox risk score, Calibration curves were performed using R package rms (v.6.3-0). Finally, decision curve analysis (DCA) was conducted to assess the clinical practicality of the nomogram model by R package "survival" and running the stdca.R file [9].

Rationale for Machine-Learning Model Selection and Integration

1. Rationale for Selecting Specific Machine-Learning Models:

The 10 machine-learning algorithms (covering 101 combinations) were chosen based on their complementary strengths in handling survival data, high-dimensional feature spaces, and potential multicollinearity, as well as their established utility in biomedical prognostication [10]:

Cox-based models (Stepwise Cox, Lasso, Ridge, Enet, CoxBoost, plsRcox): (1) Cox proportional hazards regression is the gold standard for survival analysis. However, standard Cox models face limitations in high-dimensional settings (e.g., gene expression data). (2) Lasso/Ridge/Elastic Net (Enet): These regularization techniques address overfitting and multicollinearity. Lasso (L1) performs feature selection, Ridge (L2) stabilizes coefficients, and Enet combines both to balance sparsity and stability. (3) CoxBoost: Handles high-dimensional data by iteratively selecting genes and updating coefficients, robust against noise. (4) plsRcox: Combines partial least squares (PLS) with Cox regression, reducing dimensionality while preserving prognostic information.

Random Survival Forest (RSF): Captures nonlinear relationships and interactions between genes without assuming proportional hazards. Its ensemble approach (tree-based) mitigates overfitting and improves generalizability.

Supervised Principal Components (SuperPC): Reduces dimensionality by selecting features correlated with survival before applying Cox models, ideal for datasets with more features than samples.

Generalized Boosted Regression Modeling (GBM): Iteratively builds additive models to optimize predictive performance, accommodating complex relationships between genes and survival.

Survival-SVM: Adapts support vector machines to survival outcomes, effective for nonlinear patterns in high-dimensional data.

2. Rationale for Integrating Multiple Methods

The integration of 101 algorithm combinations was driven by the following considerations:

Robustness and Consensus: No single algorithm universally outperforms others across all datasets. By combining diverse methods (parametric, nonparametric, linear, nonlinear), we ensured that the final LOXRS signature reflects consensus across models, reducing bias from any single approach.

High-Dimensional Challenges: Gene expression data often exhibit multicollinearity (e.g., co-expressed LOX/LOXL partners), sparse signals, and nonlinear interactions. Integrating regularized Cox models (Enet, Lasso), tree-based methods (RSF, GBM), and kernel-based approaches (SVM) allowed us to address these challenges comprehensively.

Stability Through Cross-Validation: Using leave-one-out cross-validation (LOOCV) in TCGA and external validation in CGGA cohorts, we prioritized models with high average C-indices across datasets. This rigorous validation ensured that the selected algorithm combination (final LOXRS formula) was stable and generalizable.

Clinical Translation: Prognostic models must balance interpretability and accuracy. By including Cox-based models (e.g., stepwise Cox, Enet), we retained clinical interpretability (e.g., hazard ratios), while RSF and SVM improved predictive performance for complex biological interactions.

3. Why These Models Were Ultimately Optimal

The final LOXRS formula (12 genes weighted by Enet-derived coefficients) and the nomogram (integrating LOXRS score, WHO grade, age, and IDH status) reflect the following:

Enet's Superiority: Elastic Net outperformed other models in average C-index across validation cohorts, likely due to its ability to handle correlated genes (e.g., LOXL1/LOXL2/LOXL4) while performing feature selection.

Biological Relevance: The retained genes (e.g., COL1A1, CASP4, CLIC1) are mechanistically linked to LOX/LOXL biology (extracellular matrix remodeling, apoptosis, ion transport), ensuring the signature's biological plausibility.

Clinical Utility: The nomogram's inclusion of established clinical factors (IDH mutation, WHO grade) alongside the LOXRS score aligns with clinical decision-making frameworks, enhancing translational relevance.

Biological pathway analysis

Single-gene differential analysis of RNA-seq was performed using DESeq2 (v.1.36.0). The differentially expressed genes (DEGs) were identified by comparing the mRNA dataset of the patients with top 30% of LOX/LOXL expression level to the dataset of bottom 30%. We filtered DEGs using adjust $p < 0.05$. Next, the Gene Set Enrichment Analysis (GSEA) was performed based on DEGs using the R package "clusterProfiler" (v.4.6.2) [11, 12], regarding "c2.cp.all.v2022.1.Hs.symbols.gmt" (https://www.gsea-msigdb.org/gsea/msigdb/index.jsp) as the reference dataset. The potential LOX/LOXL-associated pathways were inferred as significantly enriched based on false discovery rate < 0.25 and p.adjust < 0.05. Normalized enrichment score (NES) was calculated for each enriched signaling pathway. NES > 0 indicated an enrichment of upregulated pathways associated with LOX/LOXL-high status.

The ssGSEA is a rank-based algorithm that calculates a score illustrating the level of absolute enrichment of a particular gene set in each sample [13]. We collected the gene set contained in relevant pathways and feed them into ssGSEA for calculating the enrichment score of each sample in each pathway. A typical execution was to use R Bioconductor package "GSVA" (Gene Set Variation Analysis, v.1.46.0) choosing parameter as method="ssgsea". The output for each signature was a near-Gaussian list of decimals used in visualization and statistical analysis without further processing.

Single cell RNA sequencing (scRNA-seq) analysis

The UMAP of the study from Kevin Bi et al. (Cancer Cell 2021)[14] was directly obtained from the published web tool https://singlecell.broadinstitute.org/single_cell. The UMAP of GSE121638 was directly obtained from the published web tool https://immucanscdb.vital-it.ch.

Quality control, batch effect correction and cell clustering

The scRNA-seq expression datasets were imported into the R package “Seurat” (v.4.3.0) for quality control and downstream analysis of our single cell RNA-seq data [15]. All functions were run with default parameters, unless specified otherwise. Cells that expressed < 200 genes or mitochondrial gene content > 5% of the total UMI count were excluded. Before incorporating a sample into our merged dataset, we individually inspected the cells-by-genes matrix of each as a Seurat object. After merging Seurat objects, the “NormalizeData” function was used to normalize the gene expression data, and “FindVariableFeatures” was used to identify 2,000 highly variable genes. The ‘ScaleData’ were used to scale and center the expression levels of highly variable genes, excluding the influence of mitochondrial genes and the total number of molecules detected within a cell. The data was next analyzed using the principal component analysis (PCA). Batch effect correction was performed using R package "Harmony" (v.0.1.1) [16]. UMAP was subquently performed for visualizing the cells. Next, “FindNeighbors” was used to determine the k-nearest neighbors of each cell, and “FindClusters” was used to determine optimal clusters. The cluster-specific marker genes were identified by running “FindAllMarkers” function. Both R package “SingleR” (v.2.0.0) [17] and “CellMarker” website (http://xteam.xbio.top/CellMarker/) [18] were used for identifying cluster annotation. Cluster visualization, gene expression distribution and comparison in each cluster were visualized using the "scRNAtoolVis" package (v.0.0.5, https://github.com/junjunlab/scRNAtoolVis).

Cell communication analysis using CellChat in glioblastoma multiforme

To further analyze and compare cell communications difference between primary and recurrent glioma tissues, the package CellChat (v.1.6.1) was employed [19]. First, we inferred cell communications for primary and recurrent datasets separately, including n=15 primary-recurrent shared cell clusters of GABAergic neuron 1, Glutamatergic and GABAergic neuron, microglial cell, neural progenitor cell, natural killer T (NKT),

oligodentrocyte 1, oligodentrocyte 2, oligodentrocyte progenitor cell 1 (OPC-1), oligodentrocyte progenitor cell 2, PDGFRA oligodentrocyte progenitor, SLC17A7 glutamatergic neuron, astrocyte 1, astrocyte cancer stem cell, CD163 macrophage and entholial cell 1. We next analyzed the two datasets together by employing joint manifold learning and quantitative contrasts of multiple cell-cell communication networks. This approach enabled us to identify major signaling changes, as well as conserved and context-specific signaling patterns from primary status to recurrence.

The specific signaling in the cell-cell communication could be visualized and compared between the two dataset by running "netVisual_aggregate". Circle plot was used for this analysis. In addition, using the "netAnalysis_signalingRole_scatter" function, we compared outgoing and incoming interaction strengths in a 2D space. This analysis identified critical cell populations with significant changes in signal sending or receiving between primary and recurrent status. Furthermore, the critical signaling changes of cell populations identified in the last step could be shown by running "netAnalysis_signalingChanges_scatter".

scRNA-seq differential gene analysis

GSVA (Gene Set Variation Analysis) is a non-parametric, unsupervised algorithm that calculates enrichment scores for specific gene sets in each sample. GSVA provides a measure of the overall pathway activity or gene set enrichment, allowing for the identification of biological processes or pathways that are differentially regulated between different sample groups or conditions. We therefore calculated enrichemnt scores of n=50 pathways using the R package "GSVA" (v.1.46.0) and regarding the Hallmark gene sets as reference (h.all.v7.0.symbols.gmt, https://www.gsea-msigdb.org/gsea/msigdb/human/collections.jsp) in both primary and recurrent OPC-1 group in glioblastoma multiforme.

Differential expression analyses between primary and recurrent OPC-1 in glioblastoma multiforme and between *LOXL3*-positive and *LOXL3*-negative macrophages in kidney cancer were conducted using the Wilcoxon-test by running "FindMarkers" of Seurat. The differentially expressed genes were further analyzed by running Gene Set Enrichment Analysis "GSEA" from package "clusterProfiler" (v.4.6.2) to get enriched signaling pathways [11, 12].

Loss of Heterozygosity (LOH) calculation

The LOH data for each tumor sample of TCGA project were obtained from a previous study [20].

CellTiter-Glo (CTG) cell viability assay

Six types of lymphoma cell lines were cultured in RPMI 1640 medium (Gibco, 11875093) supplemented with Fetal Bovine Serum (1: 5, Gibco, A5670801) and Penicillin-Streptomycin liquid (100U/ml, Gibco, 15140122). The cells were seeded at a concentration of 5×10^4 cells/ml in 96-well plates (100 µl/well) and treated using cediranib (MedChemExpress, HY-10205) as described in figures. Cediranib was dissolved in DMSO. At the end of treatment, 100 µl of cell suspension were mixed with 100 µl of CTG reagent (Promega, G7571) and incubated for 2 min at room temperature with shaking to ensure cell lysis. The lysates were transferred into 96-well white microplates (WHB SCIENTIFIC, WHB-96-03) and luminescent signal was measured using Tecan Infinite F200 Pro Microplate Reader.

RNA isolation, cDNA synthesis and quantitative reverse transcription PCR (RT-qPCR)

The RT-qPCR methodology has been described in detail in previous publications[21]. Here is a brief overview of the experimental procedure: RNA was isolated using the AllPrep DNA/RNA mini Kit (Qiagen, 80204). cDNA synthesis was performed using the QuantiTect Reverse Transcription kit (Qiagen, 205311). RT-qPCR for the analysis of *LOX* and *LOXL1-4* gene expression was performed using a QuantStudio 6 Pro (Thermo Fisher Scientific) and SYBR Green PCR Master Mix (Thermo Fisher Scientific, 4309155). Gene expression data were normalized to the expression of β-glucuronidase (*GUS*). Gene expression of target genes was normalized to gene expression of a housekeeping gene (target/reference ratio). Primer sequences are summarized as below:

GUS:
Forward primer (5’− 3’), gaaaatatgtggttggagagctc
Reverse primer (5’− 3’), ccgagtgaagatccccttttta
LOX:
Forward primer (5’− 3’), ggatatagcggtacatatgatccttag
Reverse primer (5’− 3’), aagctctgtctgtattgtctactggt

LOXL2:

Forward primer (5'− 3'), ggagaggacatacaataccaaagtgt

Reverse primer (5'− 3'), ccatggagaatggccagtag

LOXL4:

Forward primer (5'− 3'), gctgcacaactgccacac

Reverse primer (5'− 3'), ggttgttcctgagacgctgt

Immune-related analysis

The ESTIMATE R package (v.1.0.13) was used to calculate stroma and immune infiltration on RNA sequencing data [22]. The ssGSEA scores for infiltration abundance of each individual immune cell type were standardized across all tumor samples in the investigated 33 tumor types using R package "GSVA" (v.1.46.0) [23]. The infiltration abundance of each individual immune cell type was also calculated by QUANTIseq and MCPcounter algorithms using IOBR package (version 0.99.9) and by immunCellAI [24, 25] .

Besides, the TISIDB database (http://cis.hku.hk/TISIDB) provided us the list of markers including chemokine, stimulatory, MHC and receptors [26]. Spearman's correlation coefficients between LOXL3 gene expression and mRNA expression of immunomodulators or abundance of immune cells were calculated within each cancer type. TIMER2.0 was used for assessing cancer-associated fibroblast infiltration abundance using four algorithms including EPIC, MCPCOUNTER, TIDE and XCELL [27-29].

Upstream regulation analysis of LOX/LOXL family

We utilized the genetic perturbation similarity analysis database (GPSAdb, https://www.gpsadb.com/) to identify the genes, especially transcription factors, responsible for regulating the LOX/LOXL family [30]. Based on 3048 gene knockdown/out RNA-seq datasets GPSAdb compiles information on transcriptome changes that correspond to single gene knockdown/out in cells. After identifying the upstream-related genes of LOX/LOXL via GPSAdb, we calculated expression correlation between these genes and each LOX/LOXL gene.

The microRNA (miRNA) regulation of LOX/LOXL family was explored by using the GSCAlite tool (http://bioinfo.life.hust.edu.cn/web/GSCALite/) [31]. The miRNA Network module of this tool integrates miRNA targeting information from both verified target databases and previously published prediction methods [32, 33], and utilizes negative correlation with gene expression levels to construct a comprehensive miRNA-gene regulatory network for the LOX/LOXL gene set across all cancer types.

References

1. Cerami, E., et al., *The cBio Cancer Genomics Portal: An Open Platform for Exploring Multidimensional Cancer Genomics Data.* Cancer Discovery, 2012. **2**(5): p. 401-404.
2. Gao, J., et al., *Integrative analysis of complex cancer genomics and clinical profiles using the cBioPortal.* Sci Signal, 2013. **6**(269): p. pl1.
3. Pontén, F., K. Jirström, and M. Uhlen, *The Human Protein Atlas—a tool for pathology.* The Journal of Pathology: A Journal of the Pathological Society of Great Britain and Ireland, 2008. **216**(4): p. 387-393.
4. Zhang, Y., et al., *Proteogenomic characterization of 2002 human cancers reveals pan-cancer molecular subtypes and associated pathways.* Nature Communications, 2022. **13**(1): p. 2669.
5. Chen, F., et al., *Pan-cancer molecular subtypes revealed by mass-spectrometry-based proteomic characterization of more than 500 human cancers.* Nature Communications, 2019. **10**(1): p. 5679.
6. Chandrashekar, D.S., et al., *UALCAN: An update to the integrated cancer data analysis platform.* Neoplasia, 2022. **25**: p. 18-27.
7. Liu, J., et al., *An Integrated TCGA Pan-Cancer Clinical Data Resource to Drive High-Quality Survival Outcome Analytics.* Cell, 2018. **173**(2): p. 400-416.e11.
8. Andersen, P.K. and R.D. Gill, *Cox's Regression Model for Counting Processes: A Large Sample Study.* The Annals of Statistics, 1982. **10**(4): p. 1100-1120.
9. Vickers, A.J. and E.B. Elkin, *Decision curve analysis: a novel method for evaluating prediction models.* Med Decis Making, 2006. **26**(6): p. 565-74.
10. Liu, Z., et al., *Machine learning-based integration develops an immune-derived lncRNA signature for improving outcomes in colorectal cancer.* Nat Commun, 2022. **13**(1): p. 816.
11. Wu, T., et al., *clusterProfiler 4.0: A universal enrichment tool for interpreting omics data.* The Innovation, 2021. **2**(3): p. 100141.
12. Yu, G., et al., *clusterProfiler: an R Package for Comparing Biological Themes Among Gene Clusters.* OMICS: A Journal of Integrative Biology, 2012. **16**(5): p. 284-287.
13. Hänzelmann, S., R. Castelo, and J. Guinney, *GSVA: gene set variation analysis for microarray and RNA-Seq data.* BMC Bioinformatics, 2013. **14**(1): p. 7.
14. Bi, K., et al., *Tumor and immune reprogramming during immunotherapy in advanced renal cell carcinoma.* Cancer Cell, 2021. **39**(5): p. 649-661.e5.
15. Satija, R., et al., *Spatial reconstruction of single-cell gene expression data.* Nature biotechnology, 2015. **33**(5): p. 495-502.
16. Korsunsky, I., et al., *Fast, sensitive and accurate integration of single-cell data with Harmony.* Nat Methods, 2019. **16**(12): p. 1289-1296.
17. Aran, D., et al., *Reference-based analysis of lung single-cell sequencing reveals a transitional profibrotic macrophage.* Nature immunology, 2019. **20**(2): p. 163-172.
18. Zhang, X., et al., *CellMarker: a manually curated resource of cell markers in human and mouse.* Nucleic Acids Research, 2018. **47**(D1): p. D721-D728.
19. Jin, S., et al., *Inference and analysis of cell-cell communication using CellChat.* Nature communications, 2021. **12**(1): p. 1088.

20. Thorsson, V., et al., *The Immune Landscape of Cancer.* Immunity, 2018. **48**(4): p. 812-830.e14.
21. Xu, Q., et al., *Inhibition of lysyl oxidases synergizes with 5-azacytidine to restore erythropoiesis in myelodysplastic and myeloid malignancies.* Nat Commun, 2023. **14**(1): p. 1497.
22. Yoshihara, K., et al., *Inferring tumour purity and stromal and immune cell admixture from expression data.* Nature communications, 2013. **4**(1): p. 2612.
23. Bindea, G., et al., *Spatiotemporal Dynamics of Intratumoral Immune Cells Reveal the Immune Landscape in Human Cancer.* Immunity, 2013. **39**(4): p. 782-795.
24. Zeng, D., et al., *IOBR: Multi-Omics Immuno-Oncology Biological Research to Decode Tumor Microenvironment and Signatures.* Front Immunol, 2021. **12**: p. 687975.
25. Miao, Y.R., et al., *ImmuCellAI: A Unique Method for Comprehensive T-Cell Subsets Abundance Prediction and its Application in Cancer Immunotherapy.* Adv Sci (Weinh), 2020. **7**(7): p. 1902880.
26. Ru, B., et al., *TISIDB: an integrated repository portal for tumor-immune system interactions.* Bioinformatics, 2019. **35**(20): p. 4200-4202.
27. Li, T., et al., *TIMER2.0 for analysis of tumor-infiltrating immune cells.* Nucleic Acids Res, 2020. **48**(W1): p. W509-w514.
28. Li, T., et al., *TIMER: A Web Server for Comprehensive Analysis of Tumor-Infiltrating Immune Cells.* Cancer Res, 2017. **77**(21): p. e108-e110.
29. Li, B., et al., *Comprehensive analyses of tumor immunity: implications for cancer immunotherapy.* Genome Biol, 2016. **17**(1): p. 174.
30. Guo, S., et al., *GPSAdb: a comprehensive web resource for interactive exploration of genetic perturbation RNA-seq datasets.* Nucleic Acids Research, 2023. **51**(D1): p. D964-D968.
31. Liu, C.-J., et al., *GSCALite: a web server for gene set cancer analysis.* Bioinformatics, 2018. **34**(21): p. 3771-3772.
32. Zhang, H.-M., et al., *Transcription factor and microRNA co-regulatory loops: important regulatory motifs in biological processes and diseases.* Briefings in bioinformatics, 2015. **16**(1): p. 45-58.
33. Zhang, H.-M., et al., *miR-146b-5p within BCR-ABL1–Positive Microvesicles Promotes Leukemic Transformation of Hematopoietic CellsNetwork Analysis of the Leukemia Transformation.* Cancer research, 2016. **76**(10): p. 2901-2911.

Table S1. Survival summary of TCGA patients with LOX/LOXL high vs. low expression.

	Cancer	n of patients (low/high)	HR (95% CI)	P value
**LOX**	LGGGBM	474/224	5.871 (4.375-7.879)	0
	KIRP	132/158	3.261 (1.802-5.902)	0.0008
	LGG	386/144	2.876 (1.942-4.259)	8.66E-11
	MESC	22/64	2.229 (1.397-3.558)	0.0015
	CESC	114/192	2.014 (1.257-3.225)	0.0086
	LIHC	241/132	1.998 (1.373-2.907)	5.56E-05
	SARC	122/141	1.907 (1.286-2.828)	0.0018
	BLCA	280/131	1.873 (1.365-2.568)	1.89E-05
	SKCM	126/53	1.825 (1.120-2.973)	0.0047
	STAD	242/128	1.743 (1.221-2.487)	0.0008
	UCEC	361/192	1.569 (1.018-2.420)	0.0285
	LUAD	151/379	1.540 (1.135-2.088)	0.0102
	GBM	56/112	1.527 (1.088-2.142)	0.0145
	KIRC	404/137	1.471 (1.046-2.069)	0.0155
	UVM	42/38	0.374 (0.163-0.857)	0.016
**LOXL1**	LGGGBM	423/275	6.219 (4.718-8.197)	0
	LGG	393/137	4.038 (2.575-6.332)	0
	THCA	383/129	3.617 (1.187-11.018)	0.0063
	MESC	23/63	2.549 (1.605-4.048)	0.0003
	GBM	125/43	2.053 (1.281-3.290)	0.0001
	KIRC	356/185	1.825 (1.319-2.525)	5.97E-05
	SARC	195/68	1.757 (1.087-2.841)	0.0076
	COAD	304/173	1.615 (1.069-2.439)	0.0144
	AML	70/69	1.567 (1.026-2.393)	0.0333
	STAD	144/226	1.553 (1.112-2.168)	0.0147
	LUAD	318/212	1.389 (1.033-1.869)	0.0233

	SKCM	146/311	1.365 (1.037-1.798)	0.03
	ESCA	92/71	0.526 (0.324-0.855)	0.0136
**LOXL2**	PCPG	113/71	4.581 (1.111-18.899)	0.0374
	LGGGBM	418/280	4.106 (3.180-5.303)	0
	MESO	20/66	3.458 (2.175-5.497)	3.37E-06
	UVM	38/42	2.746 (1.195-6.311)	1.38E-02
	LGG	377/153	2.562 (1.754-3.742)	1.05E-08
	CESC	144/162	2.483 (1.562-3.947)	0.0002
	KIRP	130/160	2.068 (1.145-3.735)	0.0191
	SARC	196/67	2.015 (1.238-3.279)	0.0007
	LUAD	381/149	1.952 (1.397-2.726)	5.12E-06
	PAAD	93/86	1.907 (1.264-2.879)	0.0017
	LUSC	324/172	1.592 (1.185-2.138)	0.0008
	BLCA	254/157	1.565 (1.158-2.116)	0.0024
	LIHC	171/202	1.547 (1.096-2.183)	0.0156
	UCEC	372/181	1.516 (1.001-2.366)	0.0479
	KIRC	370/171	1.497 (1.079-2.076)	0.0092
	GBM	123/45	1.486 (1.002-2.271)	0.0364
	STAD	256/114	1.478 (1.033-2.115)	2.16E-02
	OV	260/119	1.441 (1.079-1.924)	7.10E-03
	HNSC	222/281	1.336 (1.023-1.746)	0.0372
**LOXL3**	THYM	66/53	9.276 (2.502-34.384)	0.0098
	LGGGBM	478/220	4.224 (3.167-5.633)	0
	UVM	52/28	3.520 (1.451-8.543)	0.0016
	LGG	375/155	2.520 (1.709-3.715)	2.18E-08
	LIHC	181/192	1.880 (1.333-2.652)	0.0004
	UCEC	413/140	1.670 (1.017-2.742)	0.0198
	COAD	325/152	1.665 (1.089-2.546)	0.0097
	BLCA	248/163	1.547 (1.147-2.087)	0.0031

	STAD	268/102	1.522 (1.054-2.198)	0.0145
	BRCA	737/349	1.487 (1.026-2.155)	0.02
	KIRC	240/301	1.477 (1.098-1.986)	0.01
	SKCM	339/118	0.684 (0.515-0.908)	0.013
	AML	58/81	0.632 (0.410-0.974)	0.0289
	LUAD	135/395	0.622 (0.449-0.861)	0.0015
**LOXL4**	LGGGBM	396/302	5.436 (4.173-7.080)	0
	UVM	46/34	4.002 (1.665-9.618)	0.0005
	LGG	377/153	3.726 (2.424-5.729)	1.11E-16
	STAD	106/264	1.780 (1.250-2.534)	0.0047
	GBM	34/102	1.714 (1.209-2.431)	0.0043
	OV	111/268	1.645 (1.265-2.140)	0.0004
	MESC	61/25	1.635 (1.001-2.819)	0.0429
	BLCA	156/255	1.588 (1.179-2.139)	0.0043
	UCEC	404/149	1.586 (1.001-2.523)	0.031
	LIHC	192/181	1.528 (1.082-2.158)	0.015
	SARC	137/126	1.523 (1.024-2.264)	0.0356
	COAD	213/264	1.500 (1.019-2.207)	0.0406
	KIRC	209/332	0.721 (0.531-0.978)	0.03
	SKCM	236/221	0.545 (0.416-0.713)	7.21E-06
	ESCA	113/50	0.533 (0.314-0.904)	0.0426
	AML	97/42	0.397 (0.258-0.610)	0.0002
	PCPG	45/139	0.239 (0.051-1.109)	0.0267

P-value < 0.05 is considered statistically significant. Abbreviations: BRCA, Breast Cancer; UCEC, Uterine Corpus Endometrial Carcinoma; KIRC, Kidney Renal Clear Cell Carcinoma; HNSC, Head and Neck Squamous Cell Carcinoma; LUAD, Lung Adenocarcinoma; LGG, Low Grade Glioma; THCA, Thyroid Cancer; LUSC, Lung Squamous Cell Carcinoma; SKCM, Cutaneous Melanoma; COAD, Colorectal Adenocarcinoma; OV, Ovarian Cancer; STAD, Stomach Adenocarcinoma; BLCA, Bladder Urothelial Carcinoma; LIHC, Liver Hepatocellular Carcinoma; CESC, Cervical Squamous Cell Carcinoma and Endocervical Adenocarcinoma; KIRP, Kidney Renal Papillary Cell Carcinoma; SARC, Sarcoma; ESCA, Esophageal Carcinoma; PCPG, Pheochromocytoma and Paraganglioma; PAAD, Pancreatic Adenocarcinoma; GBM, Glioblastoma Multiforme; THYM, Thymoma; MESO, Malignant Mesothelioma; UVM, Uveal Melanoma.

Table S2. Summary of C-index in 101 algorithm combinations

	C-index of TCGA	C-index of CGGA#2	C-index of CGGA#1	Average of C-indices
StepCox[both]+Enet[alpha=0.6]	0.85344	0.69993	0.65291	0.73543
StepCox[backward]+Enet[alpha=0.3]	0.85344	0.69993	0.65291	0.73543
StepCox[both]+CoxBoost	0.85344	0.69993	0.65291	0.73543
RSF+Enet[alpha=0.3]	0.85312	0.69897	0.65192	0.73467
StepCox[backward]+Enet[alpha=0.2]	0.85177	0.69712	0.65416	0.73435
StepCox[both]+Enet[alpha=0.4]	0.85177	0.69712	0.65416	0.73435
StepCox[backward]+Enet[alpha=0.4]	0.85177	0.69712	0.65416	0.73435
StepCox[backward]+Enet[alpha=0.5]	0.85177	0.69712	0.65416	0.73435
RSF+Enet[alpha=0.2]	0.85150	0.70051	0.65100	0.73434
Enet[alpha=0.2]	0.85163	0.69991	0.65112	0.73422
StepCox[backward]+CoxBoost	0.84982	0.69633	0.65488	0.73367
Lasso+StepCox[both]	0.85179	0.69986	0.64892	0.73352
Lasso+StepCox[backward]	0.85179	0.69986	0.64892	0.73352
CoxBoost+StepCox[both]	0.85179	0.69986	0.64892	0.73352
CoxBoost+StepCox[backward]	0.85179	0.69986	0.64892	0.73352
Enet[alpha=0.6]	0.85367	0.69779	0.64879	0.73342
Enet[alpha=0.7]	0.85367	0.69779	0.64879	0.73342
RSF+Enet[alpha=0.6]	0.85367	0.69779	0.64879	0.73342
Lasso+CoxBoost	0.85191	0.69912	0.64771	0.73291
Enet[alpha=0.1]	0.85340	0.69573	0.64907	0.73273
RSF+Enet[alpha=0.1]	0.85340	0.69573	0.64907	0.73273
CoxBoost	0.84998	0.69491	0.65252	0.73247
StepCox[backward]+Lasso	0.84930	0.69594	0.65213	0.73246
StepCox[backward]+Enet[alpha=0.6]	0.84930	0.69594	0.65213	0.73246
StepCox[both]+Enet[alpha=0.7]	0.84930	0.69594	0.65213	0.73246
Lasso+survivalSVM	0.85204	0.69823	0.64651	0.73226

Enet[alpha=0.8]	0.85204	0.69823	0.64651	0.73226
Enet[alpha=0.9]	0.85204	0.69823	0.64651	0.73226
Lasso	0.85204	0.69823	0.64651	0.73226
Lasso+plsRcox	0.85204	0.69823	0.64651	0.73226
Lasso+StepCox[forward]	0.85204	0.69823	0.64651	0.73226
Lasso+SuperPC	0.85204	0.69823	0.64651	0.73226
RSF+Lasso	0.85204	0.69823	0.64651	0.73226
RSF+Enet[alpha=0.9]	0.85204	0.69823	0.64651	0.73226
RSF+Enet[alpha=0.8]	0.85204	0.69823	0.64651	0.73226
Lasso+GBM	0.85204	0.69823	0.64651	0.73226
Lasso+RSF	0.85204	0.69823	0.64651	0.73226
Enet[alpha=0.3]	0.85216	0.69818	0.64604	0.73213
Enet[alpha=0.5]	0.85216	0.69818	0.64604	0.73213
RSF+Enet[alpha=0.7]	0.85216	0.69818	0.64604	0.73213
RSF+Enet[alpha=0.5]	0.85216	0.69818	0.64604	0.73213
RSF+Enet[alpha=0.4]	0.85216	0.69818	0.64604	0.73213
CoxBoost+survivalSVM	0.85131	0.69681	0.64804	0.73205
CoxBoost+Ridge	0.85131	0.69681	0.64804	0.73205
CoxBoost+Enet[alpha=0.1]	0.85131	0.69681	0.64804	0.73205
CoxBoost+Enet[alpha=0.3]	0.85131	0.69681	0.64804	0.73205
CoxBoost+Enet[alpha=0.2]	0.85131	0.69681	0.64804	0.73205
CoxBoost+Enet[alpha=0.4]	0.85131	0.69681	0.64804	0.73205
CoxBoost+Enet[alpha=0.5]	0.85131	0.69681	0.64804	0.73205
CoxBoost+Enet[alpha=0.6]	0.85131	0.69681	0.64804	0.73205
CoxBoost+Enet[alpha=0.7]	0.85131	0.69681	0.64804	0.73205
CoxBoost+Enet[alpha=0.8]	0.85131	0.69681	0.64804	0.73205
CoxBoost+Enet[alpha=0.9]	0.85131	0.69681	0.64804	0.73205
CoxBoost+Lasso	0.85131	0.69681	0.64804	0.73205
CoxBoost+plsRcox	0.85131	0.69681	0.64804	0.73205

CoxBoost+StepCox[forward]	0.85131	0.69681	0.64804	0.73205
CoxBoost+SuperPC	0.85131	0.69681	0.64804	0.73205
RSF+CoxBoost	0.85131	0.69681	0.64804	0.73205
CoxBoost+GBM	0.85131	0.69681	0.64804	0.73205
CoxBoost+RSF	0.85131	0.69681	0.64804	0.73205
RSF+StepCox[both]	0.85348	0.69339	0.64743	0.73143
RSF+StepCox[backward]	0.85348	0.69339	0.64743	0.73143
StepCox[both]+Ridge	0.85348	0.69339	0.64743	0.73143
StepCox[backward]+Ridge	0.85348	0.69339	0.64743	0.73143
StepCox[both]+plsRcox	0.85348	0.69339	0.64743	0.73143
StepCox[backward]+plsRcox	0.85348	0.69339	0.64743	0.73143
StepCox[both]+Enet[alpha=0.9]	0.85348	0.69339	0.64743	0.73143
StepCox[backward]+Enet[alpha=0.9]	0.85348	0.69339	0.64743	0.73143
StepCox[both]+Enet[alpha=0.1]	0.85348	0.69339	0.64743	0.73143
StepCox[backward]+Enet[alpha=0.1]	0.85348	0.69339	0.64743	0.73143
StepCox[both]+Enet[alpha=0.8]	0.85348	0.69339	0.64743	0.73143
StepCox[backward]+Enet[alpha=0.8]	0.85348	0.69339	0.64743	0.73143
StepCox[both]+Enet[alpha=0.2]	0.85348	0.69339	0.64743	0.73143
StepCox[both]+Lasso	0.85348	0.69339	0.64743	0.73143
StepCox[backward]+Enet[alpha=0.7]	0.85348	0.69339	0.64743	0.73143
StepCox[both]	0.85348	0.69339	0.64743	0.73143
StepCox[backward]	0.85348	0.69339	0.64743	0.73143
StepCox[both]+Enet[alpha=0.3]	0.85348	0.69339	0.64743	0.73143
StepCox[both]+Enet[alpha=0.5]	0.85348	0.69339	0.64743	0.73143
StepCox[both]+survivalSVM	0.85348	0.69339	0.64743	0.73143
StepCox[backward]+survivalSVM	0.85348	0.69339	0.64743	0.73143
StepCox[both]+GBM	0.85348	0.69339	0.64743	0.73143
StepCox[backward]+GBM	0.85348	0.69339	0.64743	0.73143
StepCox[both]+RSF	0.85348	0.69339	0.64743	0.73143

StepCox[backward]+RSF	0.85348	0.69339	0.64743	0.73143
StepCox[both]+SuperPC	0.85348	0.69339	0.64743	0.73143
StepCox[backward]+SuperPC	0.85348	0.69339	0.64743	0.73143
Enet[alpha=0.4]	0.85022	0.69311	0.65075	0.73136
survivalSVM	0.85637	0.68909	0.64058	0.72868
Ridge	0.85637	0.68909	0.64058	0.72868
SuperPC	0.85637	0.68909	0.64058	0.72868
RSF+survivalSVM	0.85637	0.68909	0.64058	0.72868
StepCox[forward]	0.85637	0.68909	0.64058	0.72868
plsRcox	0.85637	0.68909	0.64058	0.72868
RSF+Ridge	0.85637	0.68909	0.64058	0.72868
RSF+plsRcox	0.85637	0.68909	0.64058	0.72868
RSF+StepCox[forward]	0.85637	0.68909	0.64058	0.72868
RSF+SuperPC	0.85637	0.68909	0.64058	0.72868
RSF	0.85637	0.68909	0.64058	0.72868
RSF+GBM	0.85637	0.68909	0.64058	0.72868
GBM	0.85637	0.68909	0.64058	0.72868

Table S3. Examples of survival prediction in nomogram model.

Patient	LOX score	WHO grade	Age	IDH mutation	Total points by nomogram model	Predicted survival probability	Real survival (years)
CGGA_842	4.58	IV	>40	No	122	3-year <0.2<br>5-year <0.2<br>7-year <0.2	0.57
CGGA_698	-0.41	II	>40	Yes	46	3-year >0.8<br>5-year 0.6-0.7<br>7-year 0.4-0.6	5.14
CGGA_1031	-1.09	II	<40	Yes	28	3-year >0.8<br>5-year >0.8<br>7-year 0.7-0.8	6.83
TCGA-S9-A6TU-01A-12R-A32Q-07	-1.80	I-II	<40	Yes	15	3-year >0.8<br>5-year >0.8<br>7-year >0.8	7.26
TCGA-HT-7854-01A-11R-2256-07	0.71	I-II	>40	No	45	3-year >0.8<br>5-year 0.6-0.8<br>7-year 0.4-0.6	3.29
TCGA-27-1832-01A-01R-1850-01	3.50	III-IV	>40	No	115	3-year <0.2<br>5-year <0.2<br>7-year <0.2	0.83
TCGA-S9-A6WI-01A-21R-A33Z-07	-1.25	I-II	>40	Yes	35	3-year >0.8<br>5-year >0.8<br>7-year 0.7-0.8	7.08

Table S4. LOX/LOXL expression (log_2(TPM+1)) of lymphoma cell lines in CCLE dataset.

	LOX	*LOXL1*	*LOXL2*	*LOXL3*	*LOXL4*	**Lymphoma**
**NAMALWA**	0.14	0.05	0.69	0.27	0.17	Burkitt lymphoma
**Mino**	0.10	0.00	0.94	0.25	0.07	mantle cell lymphoma
**DB**	0.12	0.00	1.93	0.19	0.03	DLBCL
**SU-DHL-6**	0.43	0.26	4.87	0.41	0.25	B-NHL, diffuse, mixed small and large cell type
**KARPAS-422**	0.28	0.01	7.00	0.56	0.06	DLBCL
**WSU-DLCL2**	0.67	0.41	8.84	1.20	0.33	DLBCL

Figure S1

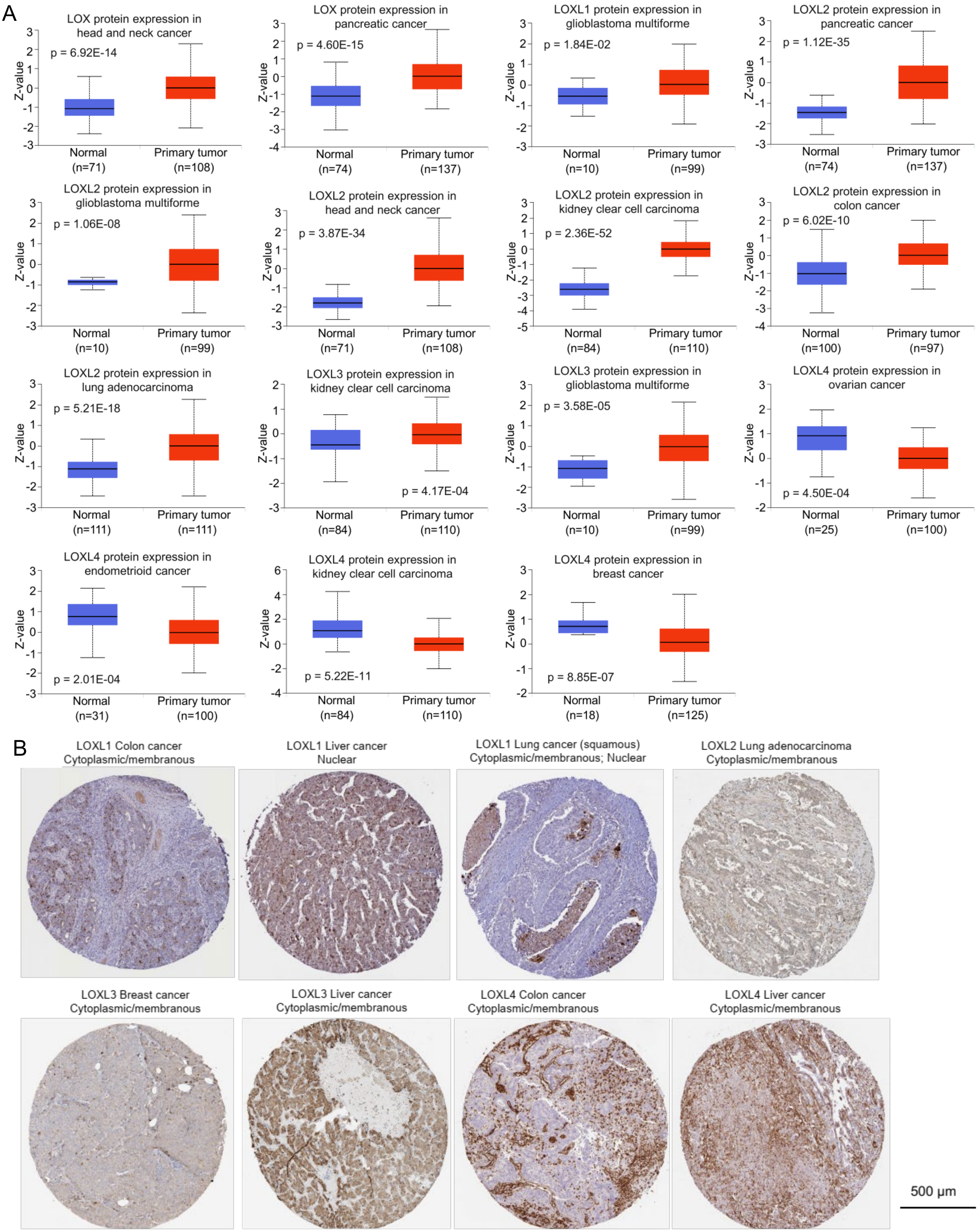

Figure S1. LOX/LOXL protein expression in pan cancer. (A) LOX and LOXL1-4 protein expression was compared between healthy tissues and tumor using the UALCAN portal. P-value < 0.05 is considered statistically significant; (B) Immunohistochemical detection of LOX/LOXL protein in primary tumor specimens obtained from colon, liver, lung and breast cancers. Scanning images of immunohistochemical staining were obtained from HPA database. LOX/LOXL positive areas are indicated in brown color.

Figure S2

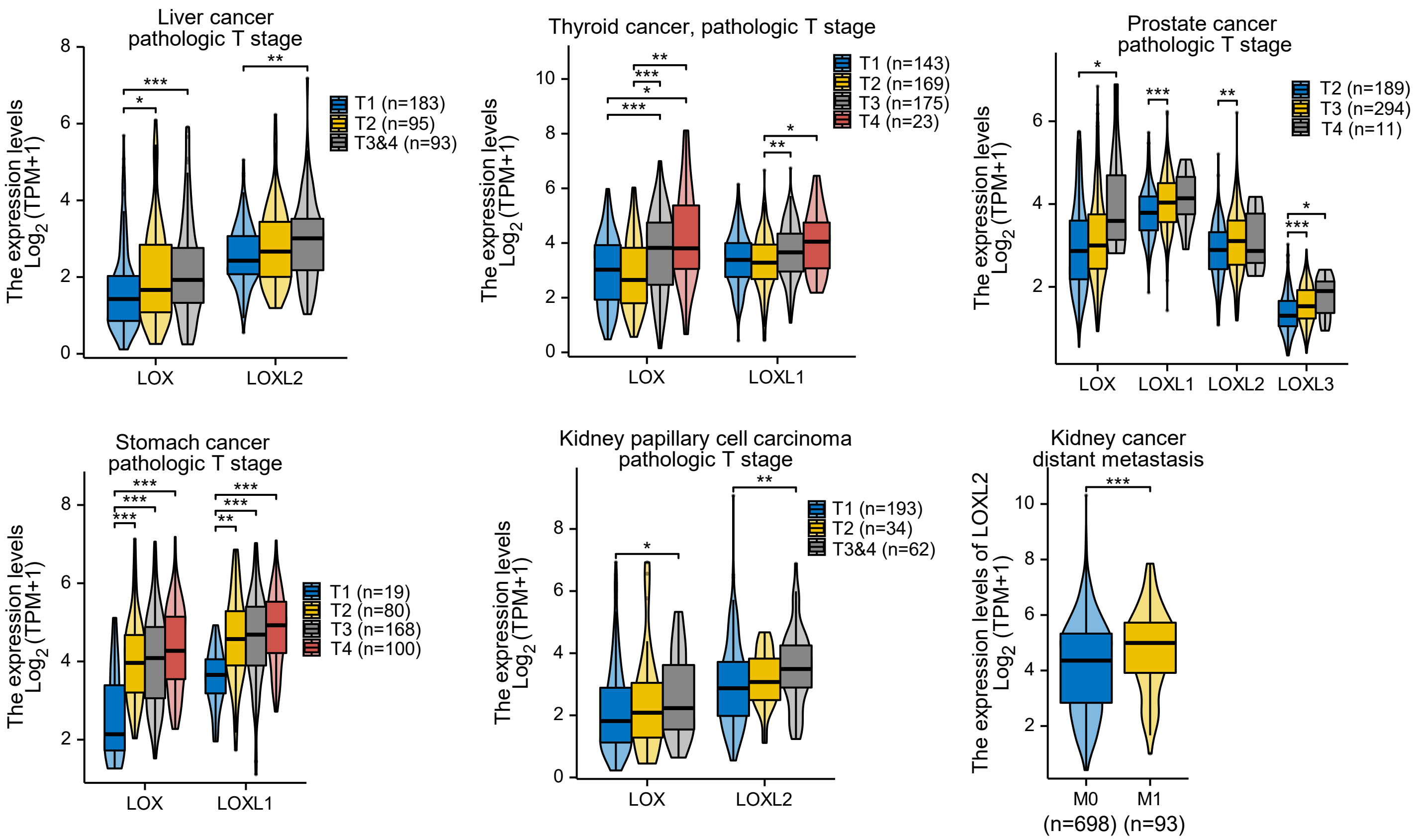

Figure S2. The comparison of LOX/LOXL expression between different tumor (T) stages and distant metastasis status. All boxplots and violin plots represent the median and quartiles of the data. Appropriate statistical methods were automatically chosen based on the characteristics of the data format using the statistical R packages "stats" (v.4.2.1) and "car" (v.3.1-0). * p<0.05, **p<0.01, ***p<0.001. P-value < 0.05 is considered statistically significant.

Figure S3

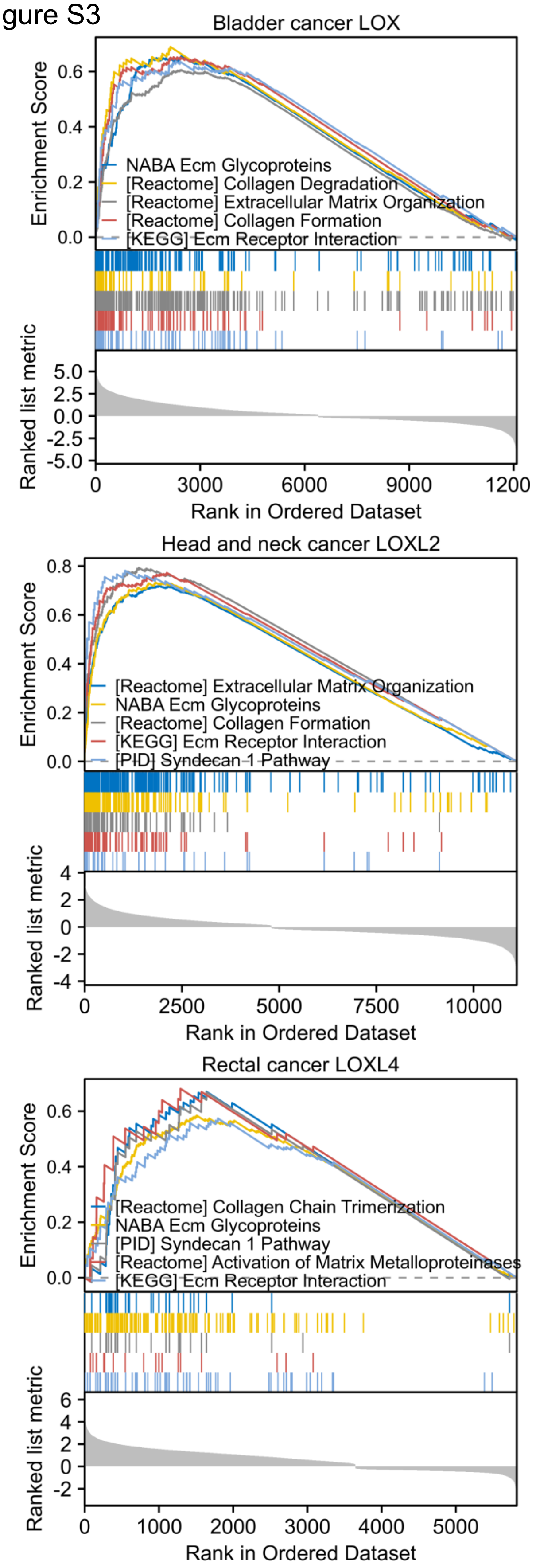

Figure S3. Extracellular matrix-related pathway enrichment is associated with LOX and LOXL1-4 overexpression. The pathways were enriched by the Gene Set Enrichment Analysis (GSEA).

Figure S4

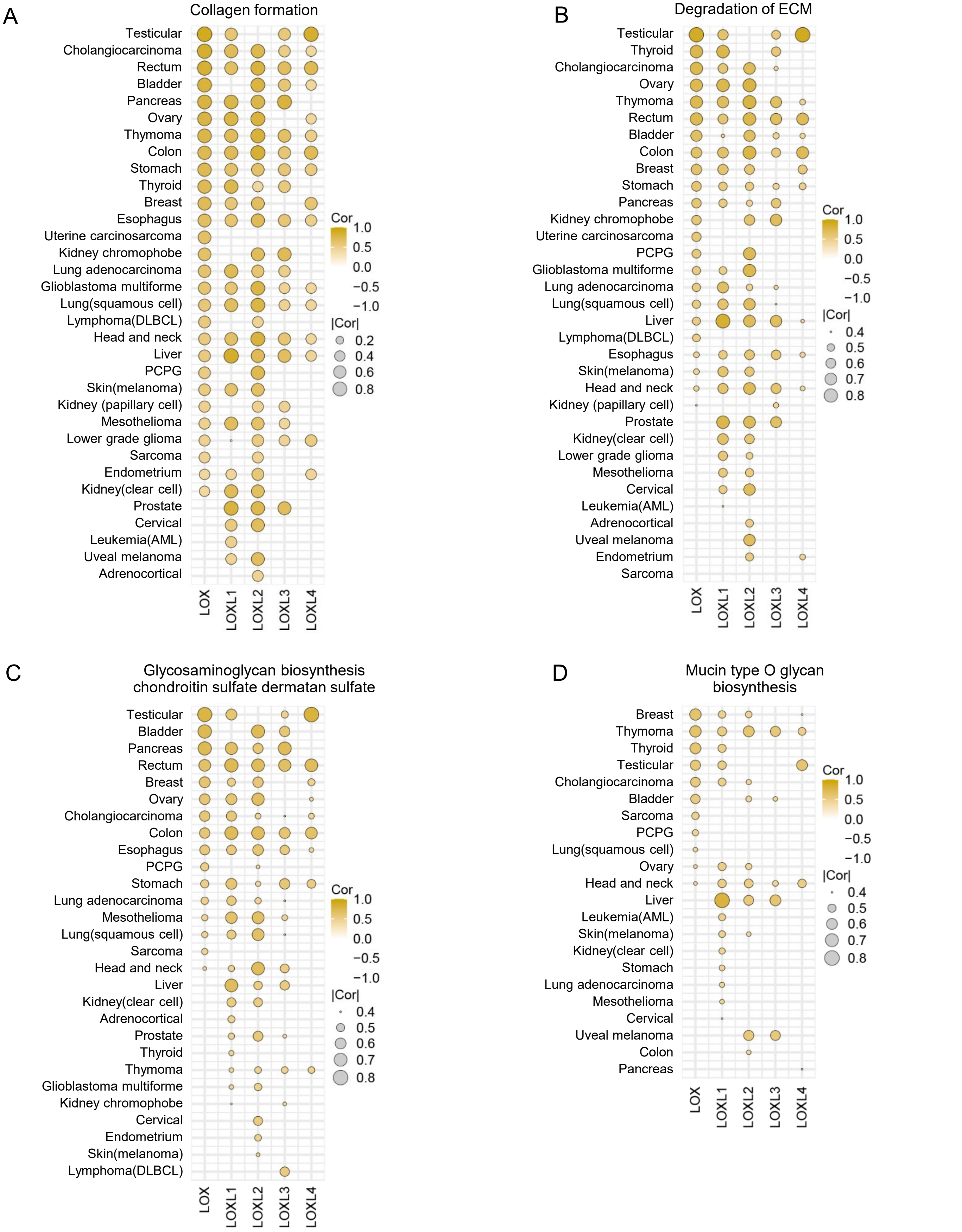

Figure S4. Correlation between LOX/LOXL expression and enrichment score of extracellular matrix (ECM) pathways, including collagen formation (A), degradation of ECM (B), glycosaminoglycan biosynthesis chondroitin sulfate dermatan sulfate (C), and mucin type O glycan biosynthesis (D). The enrichment score was calculated by the R package "GSVA" (Gene Set Variation Analysis, v.1.46.0) and correlated with LOX/LOXL expression by the spearman correlation analysis. The size of circles indicated spearman's r values. Only results with p<0.05 were displayed.

Figure S5

A LOX

	EPIC	MCPCOUNTER	TIDE	XCELL
Adrenocortical				
Bladder	0.74	0.75	0.78	
Breast	0.80	0.79	0.81	
Cervical				
Cholangiocarcinoma	0.73	0.76	0.81	
Colon	0.88	0.87	0.76	0.59
Lymphoma(DLBCL)	0.66	0.58	0.53	
Esophagus	0.68	0.66	0.70	
Glioblastoma multiforme	0.51	0.58	0.65	
Head and neck	0.55	0.53	0.50	
Kidney chromophobe	0.61	0.41	0.63	
Kidney(clear cell)				
Kidney (papillary cell)	0.42		0.48	
Lower grade glioma				
Liver	0.44		0.48	
Lung adenocarcinoma	0.66	0.72	0.76	
Lung(squamous cell)	0.57	0.55	0.60	
Mesothelioma	0.60	0.62	0.54	
Ovary	0.77	0.75	0.71	
Pancreas	0.84	0.82	0.85	0.47
PCPG	0.46	0.57	0.63	
Prostate				
Rectum	0.90	0.78	0.77	
Sarcoma	0.62		0.52	
Skin(melanoma)	0.49	0.53	0.61	
Stomach	0.83	0.77	0.72	0.46
Testicular	0.87	0.86	0.88	0.56
Thyroid	0.67	0.53	0.51	
Thymoma	0.80	0.61	0.80	
Endometrium			0.41	
Uterine carcinosarcoma	0.61	0.55	0.67	
Uveal melanoma				

B LOXL1

	EPIC	MCPCOUNTER	TIDE	XCELL
Adrenocortical				
Bladder		0.43	0.40	0.44
Breast	0.72	0.74	0.71	0.60
Cervical	0.44	0.48	0.51	
Cholangiocarcinoma	0.49	0.61	0.58	0.45
Colon	0.78	0.78	0.76	0.64
Lymphoma(DLBCL)				0.63
Esophagus	0.69	0.70	0.67	0.61
Glioblastoma multiforme	0.43	0.48	0.63	
Head and neck	0.69	0.71	0.60	0.66
Kidney chromophobe				
Kidney(clear cell)	0.62	0.65	0.57	
Kidney (papillary cell)				0.40
Lower grade glioma		0.50	0.45	
Liver	0.67	0.90	0.69	0.59
Lung adenocarcinoma	0.60	0.65	0.66	0.47
Lung(squamous cell)	0.59	0.63	0.62	
Mesothelioma	0.68	0.76	0.59	
Ovary	0.67	0.69	0.72	
Pancreas	0.71	0.70	0.62	0.43
PCPG				
Prostate	0.71	0.69	0.69	0.57
Rectum	0.81	0.81	0.72	0.56
Sarcoma				
Skin(melanoma)	0.62	0.65	0.46	
Stomach	0.60	0.67	0.74	0.64
Testicular	0.61	0.66	0.59	0.59
Thyroid	0.76	0.68	0.62	
Thymoma	0.61	0.55	0.60	0.55
Endometrium				
Uterine carcinosarcoma				
Uveal melanoma		0.51	0.50	

C LOXL2

	EPIC	MCPCOUNTER	TIDE	XCELL
Adrenocortical	0.52	0.44		
Bladder	0.70	0.73	0.76	
Breast	0.71	0.75	0.74	
Cervical	0.55	0.62	0.75	
Cholangiocarcinoma	0.77	0.76	0.75	
Colon	0.85	0.85	0.80	0.52
Lymphoma(DLBCL)				
Esophagus	0.75	0.72	0.58	
Glioblastoma multiforme	0.63	0.65	0.72	
Head and neck	0.78	0.79	0.84	
Kidney chromophobe	0.69	0.56	0.51	
Kidney(clear cell)	0.54	0.59	0.63	
Kidney (papillary cell)			0.42	
Lower grade glioma			0.41	
Liver	0.67	0.74	0.58	
Lung adenocarcinoma	0.67	0.65	0.57	
Lung(squamous cell)	0.73	0.75	0.80	
Mesothelioma	0.64	0.67	0.47	
Ovary	0.79	0.78	0.76	
Pancreas	0.76	0.75	0.69	
PCPG	0.47	0.52	0.42	
Prostate	0.73	0.58	0.70	
Rectum	0.79	0.69	0.70	
Sarcoma	0.42			
Skin(melanoma)	0.56	0.67	0.67	
Stomach	0.64	0.55	0.43	
Testicular				
Thyroid	0.49	0.60	0.52	
Thymoma	0.76	0.68	0.75	0.54
Endometrium			0.57	
Uterine carcinosarcoma		0.42	0.52	
Uveal melanoma	0.64	0.55	0.47	

D LOXL3

	EPIC	MCPCOUNTER	TIDE	XCELL
Adrenocortical				
Bladder	0.55	0.59	0.55	
Breast				
Cervical	0.51	0.50	0.40	0.42
Cholangiocarcinoma	0.53	0.58	0.55	
Colon	0.74	0.75	0.62	0.53
Lymphoma(DLBCL)	0.51			
Esophagus	0.66	0.67	0.61	0.52
Glioblastoma multiforme	0.43		0.47	
Head and neck	0.67	0.67	0.57	0.54
Kidney chromophobe	0.52		0.45	
Kidney(clear cell)	0.48	0.47		
Kidney (papillary cell)		0.45		
Lower grade glioma				
Liver	0.53	0.56		
Lung adenocarcinoma	0.57	0.58	0.44	
Lung(squamous cell)	0.61	0.62	0.54	
Mesothelioma	0.51	0.46		
Ovary				
Pancreas	0.84	0.83	0.82	0.49
PCPG				
Prostate	0.75	0.43	0.59	
Rectum	0.74	0.67	0.59	
Sarcoma				
Skin(melanoma)			0.41	
Stomach	0.63	0.61	0.58	0.43
Testicular	0.59	0.56	0.56	
Thyroid	0.66	0.64	0.46	
Thymoma	0.65	0.51	0.75	
Endometrium				
Uterine carcinosarcoma				
Uveal melanoma			0.51	

E LOXL4

	EPIC	MCPCOUNTER	TIDE	XCELL
Adrenocortical				
Bladder			0.40	
Breast				
Cervical				
Cholangiocarcinoma			0.54	
Colon	0.72	0.79	0.72	0.65
Lymphoma(DLBCL)				
Esophagus		0.41	0.53	0.41
Glioblastoma multiforme			0.43	
Head and neck	0.43	0.43		0.51
Kidney chromophobe			0.55	
Kidney(clear cell)				
Kidney (papillary cell)				
Lower grade glioma	0.40	0.40	0.49	
Liver				
Lung adenocarcinoma				
Lung(squamous cell)				
Mesothelioma	0.47			
Ovary				
Pancreas				
PCPG				
Prostate				
Rectum	0.58	0.77	0.72	0.55
Sarcoma				
Skin(melanoma)				
Stomach	0.41	0.56	0.68	0.51
Testicular	0.88	0.91	0.88	0.64
Thyroid				
Thymoma			0.60	
Endometrium	0.44	0.56	0.47	
Uterine carcinosarcoma			0.48	
Uveal melanoma				

Figure S5. Correlation between LOX/LOXL expression and infiltration abundance of cancer-associated fibroblasts (CAF) in pan cancer (A-E, LOX, LOXL1-LOXL4). The infiltration abundance of CAF was calculated by TIMER2.0 using four algorithms including EPIC, MCPCOUNTER, TIDE and XCELL, and correlated with LOX/LOXL expression by the spearman correlation analysis. Only results with $p<0.05$ were displayed.

Figure S6

A

	Vascular function					Cell migration and invasion					Cell junction and adhesion					Integrin signaling				
	LOX	LOXL1	LOXL2	LOXL3	LOXL4	LOX	LOXL1	LOXL2	LOXL3	LOXL4	LOX	LOXL1	LOXL2	LOXL3	LOXL4	LOX	LOXL1	LOXL2	LOXL3	LOXL4
Adrenocortical	0	0	0	0	0	0	0	1	1	0	0	0	0	0	0	0	0	4	0	0
Bladder	2	2	4	1	0	4	3	6	6	2	3	2	5	1	1	9	8	9	6	5
Breast	5	0	4	1	0	5	0	9	1	1	5	0	4	0	2	11	0	13	5	2
Cervical	0	0	0	0	0	0	1	4	1	0	0	0	1	0	0	0	3	4	3	0
Cholangiocarcinoma	1	0	0	0	0	1	0	0	0	0	1	0	0	0	0	4	0	1	1	0
Colon	2	3	8	1	2	2	4	8	2	2	2	3	10	1	4	5	7	9	3	4
Lymphoma(DLBCL)	1	0	0	5	0	2	0	1	3	0	0	2	0	2	0	5	0	4	6	0
Esophagus	0	1	3	1	0	0	1	4	3	0	0	0	2	2	0	4	5	10	9	3
Glioblastoma multiforme	1	1	2	3	1	1	4	3	4	2	1	1	4	4	0	8	10	11	12	9
Head and neck	6	2	10	0	0	5	4	10	1	0	4	1	11	1	0	10	4	15	5	3
Kidney chromophobe	0	0	1	0	0	0	0	0	1	0	0	0	1	1	0	3	0	2	5	0
Kidney(clear cell)	1	0	8	7	0	0	0	6	1	0	0	0	3	1	0	4	3	5	6	0
Kidney (papillary cell)	0	0	1	3	0	0	1	2	1	1	0	0	2	2	1	0	3	4	6	5
Leukemia(AML)	0	0	0	0	0	0	1	1	0	0	0	0	0	0	0	0	4	4	0	0
Lower grade glioma	1	0	2	1	0	2	0	4	2	1	1	0	3	2	1	5	4	8	7	2
Liver	0	0	1	2	0	1	2	3	4	0	2	1	5	3	0	3	4	5	5	0
Lung adenocarcinoma	4	3	2	4	0	7	4	7	4	0	2	1	2	3	0	8	9	10	7	0
Lung(squamous cell)	3	2	3	5	2	5	8	5	5	2	5	4	5	1	2	8	9	11	6	7
Mesothelioma	0	0	3	0	0	2	1	7	2	0	0	0	5	1	1	4	6	7	3	3
Ovary	5	3	8	0	7	6	6	10	2	6	2	3	4	3	4	11	8	10	3	11
Pancreas	5	0	0	5	0	7	0	0	4	0	6	0	0	3	0	12	0	0	10	1
PCPG	4	0	5	0	0	4	0	3	0	0	2	0	0	0	0	9	0	4	0	0
Prostate	0	2	10	5	3	0	5	11	5	3	0	5	7	3	5	0	11	10	10	4
Rectum	2	1	1	1	2	4	3	2	1	1	1	2	0	1	0	7	0	5	5	5
Sarcoma	0	0	0	0	0	0	0	2	0	0	0	0	0	0	0	0	0	0	0	0
Skin(melanoma)	0	0	1	0	0	2	0	0	0	0	1	1	0	1	0	5	4	4	4	0
Stomach	5	5	6	2	2	8	6	10	8	2	3	3	2	4	2	10	9	10	9	4
Testicular	2	1	0	1	1	1	1	0	1	1	5	4	0	5	2	4	4	0	4	4
Thyroid	0	0	1	0	0	2	0	1	1	0	5	5	0	3	3	11	8	4	2	0
Thymoma	1	1	1	5	0	1	2	3	4	1	2	3	4	8	7	7	5	6	9	6
Endometrium	0	0	5	0	0	1	8	8	2	0	1	9	5	1	1	6	3	9	2	4
Uterine carcinosarcoma	0	0	0	0	0	0	0	0	0	1	0	0	0	0	1	2	0	0	0	0
Uveal melanoma	0	0	0	0	0	0	0	0	0	0	0	0	0	2	1	0	0	0	2	0

B

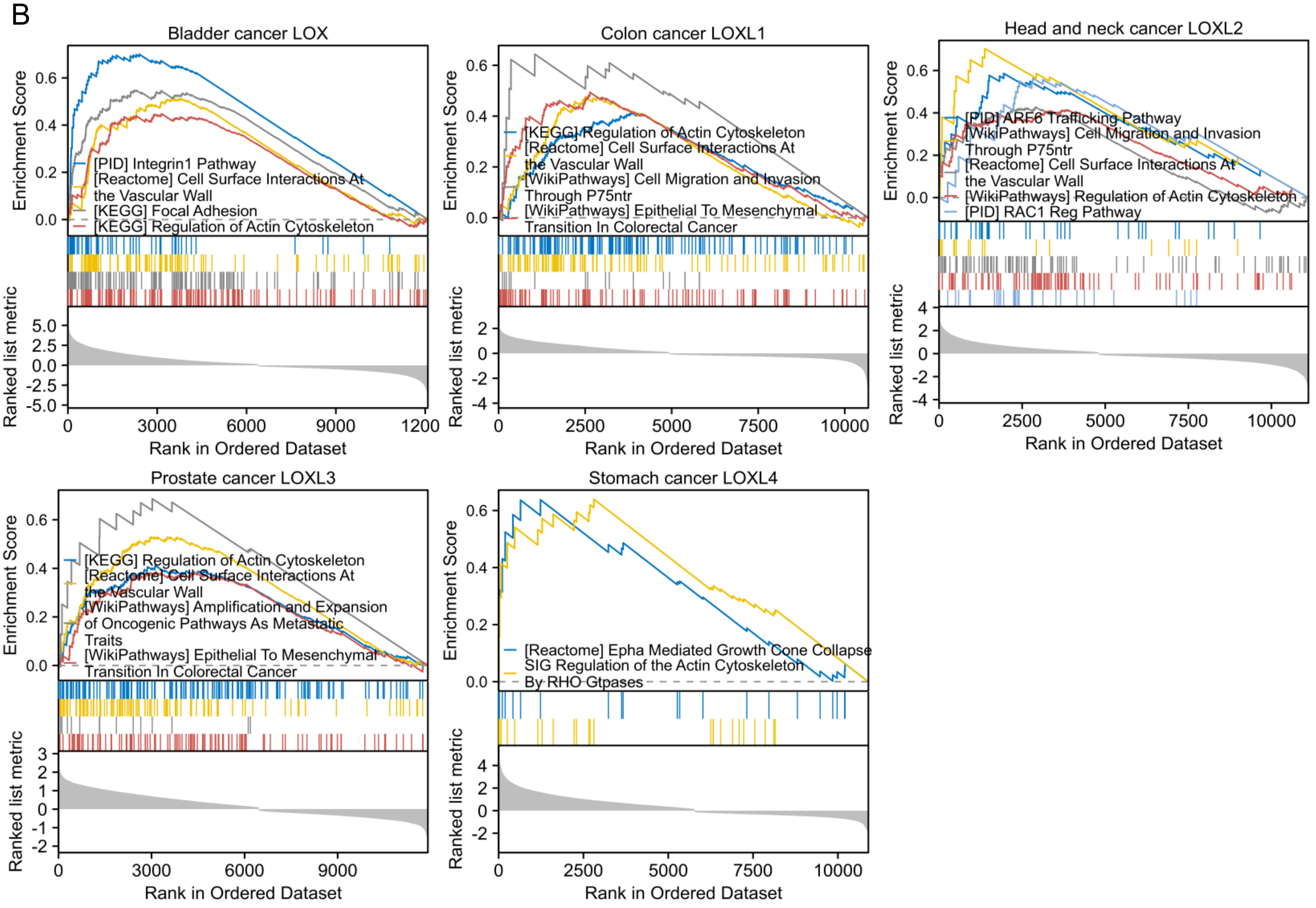

Figure S6. Enrichment of activated pathways including vascular function, cell migration and invasion, cell junction and adhesion, as well as integrin signaling. (A) Count summary of activated pathways in pan cancer. (B) Examples showing the activated pathways enriched by the Gene Set Enrichment Analysis (GSEA) in case of LOX/LOXL overexpression.

Figure S7

Wnt signaling pathway

	LOX	LOXL1	LOXL2	LOXL3	LOXL4
Adrenocortical	0	0	1	0	0
Bladder	0	0	1	0	1
Breast	4	0	4	1	3
Cervical	0	0	2	0	0
Cholangiocarcinoma	6	0	0	0	0
Colon	0	5	6	0	0
Lymphoma(DLBCL)	3	0	0	1	0
Esophagus	0	1	0	5	4
Glioblastoma multiforme	2	5	4	3	4
Head and neck	1	7	11	0	0
Kidney chromophobe	0	0	1	0	0
Kidney(clear cell)	0	3	6	0	0
Kidney (papillary cell)	0	9	1	1	5
Leukemia(AML)	0	0	0	0	0
Lower grade glioma	0	0	2	1	0
Liver	4	5	8	8	0
Lung adenocarcinoma	3	5	0	0	0
Lung(squamous cell)	2	9	2	8	7
Mesothelioma	0	0	2	0	0
Ovary	3	10	8	6	7
Pancreas	5	0	0	7	0
PCPG	2	0	1	0	0
Prostate	0	4	12	7	4
Rectum	3	4	0	0	0
Sarcoma	0	0	4	0	0
Skin(melanoma)	4	2	2	0	0
Stomach	7	10	2	10	0
Testicular	7	4	0	10	5
Thyroid	0	1	0	1	0
Thymoma	1	0	3	5	0
Endometrium	1	9	11	2	2
Uterine carcinosarcoma	0	0	0	0	0
Uveal melanoma	0	0	0	0	0

GPCRs-related

	LOX	LOXL1	LOXL2	LOXL3	LOXL4
Adrenocortical	0	0	4	0	0
Bladder	7	0	10	8	6
Breast	3	0	0	5	4
Cervical	0	0	5	3	0
Cholangiocarcinoma	2	0	0	5	0
Colon	8	0	14	9	12
Lymphoma(DLBCL)	1	0	0	8	0
Esophagus	2	0	5	6	0
Glioblastoma multiforme	2	0	5	0	3
Head and neck	13	0	15	7	8
Kidney chromophobe	0	0	4	7	0
Kidney(clear cell)	10	0	2	5	0
Kidney (papillary cell)	0	0	11	12	9
Leukemia(AML)	0	0	1	0	0
Lower grade glioma	1	0	1	0	4
Liver	11	0	7	9	0
Lung adenocarcinoma	10	0	0	11	1
Lung(squamous cell)	11	0	9	13	4
Mesothelioma	0	0	5	9	0
Ovary	8	0	12	15	10
Pancreas	7	0	0	5	1
PCPG	7	0	1	0	0
Prostate	0	0	13	11	11
Rectum	5	0	4	5	8
Sarcoma	0	0	0	0	0
Skin(melanoma)	9	0	7	6	0
Stomach	9	0	3	13	11
Testicular	4	0	0	9	4
Thyroid	5	2	8	7	0
Thymoma	10	9	11	13	9
Endometrium	8	8	3	5	10
Uterine carcinosarcoma	3	0	0	0	0
Uveal melanoma	0	0	0	10	3

TGF-beta signaling pathway

	LOX	LOXL1	LOXL2	LOXL3	LOXL4
Adrenocortical	0	0	1	0	0
Bladder	1	2	0	0	0
Breast	7	0	8	1	0
Cervical	0	0	3	0	0
Cholangiocarcinoma	1	0	0	0	0
Colon	0	3	4	0	0
Lymphoma(DLBCL)	4	0	0	2	0
Esophagus	0	3	3	0	0
Glioblastoma multiforme	3	5	4	4	3
Head and neck	4	1	9	0	0
Kidney chromophobe	1	0	0	0	0
Kidney(clear cell)	0	0	6	1	0
Kidney (papillary cell)	0	0	1	0	0
Leukemia(AML)	0	0	0	0	0
Lower grade glioma	0	0	6	0	0
Liver	0	0	0	2	0
Lung adenocarcinoma	2	5	2	1	0
Lung(squamous cell)	4	2	6	0	2
Mesothelioma	3	4	5	1	0
Ovary	5	5	4	1	5
Pancreas	6	0	0	4	0
PCPG	4	0	1	0	0
Prostate	0	2	4	3	2
Rectum	1	3	0	1	1
Sarcoma	0	0	0	0	0
Skin(melanoma)	2	0	1	0	0
Stomach	7	4	5	2	0
Testicular	1	2	0	1	1
Thyroid	1	0	1	0	0
Thymoma	3	1	2	2	3
Endometrium	3	0	8	0	0
Uterine carcinosarcoma	0	0	0	0	0
Uveal melanoma	0	0	0	0	0

PI3K-related signaling pathway

	LOX	LOXL1	LOXL2	LOXL3	LOXL4
Adrenocortical	0	0	2	0	0
Bladder	4	3	8	7	2
Breast	3	0	2	3	0
Cervical	0	2	7	5	0
Cholangiocarcinoma	2	0	0	1	0
Colon	2	2	2	2	2
Lymphoma(DLBCL)	2	0	2	2	0
Esophagus	2	4	2	3	2
Glioblastoma multiforme	2	2	2	5	2
Head and neck	2	6	2	3	5
Kidney chromophobe	2	0	0	3	0
Kidney(clear cell)	2	0	0	3	0
Kidney (papillary cell)	0	0	2	6	2
Leukemia(AML)	0	2	2	0	0
Lower grade glioma	2	2	4	4	0
Liver	2	4	2	6	0
Lung adenocarcinoma	6	2	2	6	0
Lung(squamous cell)	6	3	4	8	2
Mesothelioma	2	2	2	2	0
Ovary	5	6	5	8	5
Pancreas	3	0	0	3	0
PCPG	6	0	2	0	0
Prostate	0	7	8	6	7
Rectum	2	0	2	2	2
Sarcoma	0	0	0	0	0
Skin(melanoma)	8	2	4	2	0
Stomach	3	3	2	4	2
Testicular	4	2	0	4	4
Thyroid	6	4	2	4	0
Thymoma	5	4	5	5	4
Endometrium	2	4	9	0	3
Uterine carcinosarcoma	1	0	0	0	0
Uveal melanoma	0	0	0	2	2

MET signaling pathway

	LOX	LOXL1	LOXL2	LOXL3	LOXL4
Adrenocortical	0	0	3	0	0
Bladder	3	3	3	1	1
Breast	3	0	3	3	0
Cervical	0	2	3	2	0
Cholangiocarcinoma	3	0	0	0	0
Colon	2	2	3	0	1
Lymphoma(DLBCL)	3	0	3	3	0
Esophagus	3	3	3	3	0
Glioblastoma multiforme	3	3	3	3	3
Head and neck	3	3	3	0	0
Kidney chromophobe	3	0	3	0	0
Kidney(clear cell)	3	1	3	2	0
Kidney (papillary cell)	0	3	3	3	1
Leukemia(AML)	0	3	1	0	0
Lower grade glioma	3	0	3	3	0
Liver	3	3	3	2	0
Lung adenocarcinoma	3	3	3	3	0
Lung(squamous cell)	3	3	3	3	3
Mesothelioma	3	3	3	3	1
Ovary	3	3	3	0	3
Pancreas	4	0	0	3	0
PCPG	3	0	3	0	0
Prostate	0	3	3	2	1
Rectum	3	0	3	3	0
Sarcoma	0	0	2	0	0
Skin(melanoma)	2	0	0	0	0
Stomach	3	2	4	3	0
Testicular	2	3	0	3	2
Thyroid	2	2	3	2	0
Thymoma	3	2	3	3	3
Endometrium	2	2	3	0	1
Uterine carcinosarcoma	0	0	0	0	0
Uveal melanoma	0	0	0	0	0

JAK/STAT signaling pathway

	LOX	LOXL1	LOXL2	LOXL3	LOXL4
Adrenocortical	0	0	1	0	0
Bladder	1	0	1	1	0
Breast	0	0	0	0	0
Cervical	0	0	0	0	0
Cholangiocarcinoma	0	0	0	0	0
Colon	1	1	1	0	0
Lymphoma(DLBCL)	1	0	0	2	0
Esophagus	0	0	1	0	0
Glioblastoma multiforme	1	1	1	2	1
Head and neck	0	0	1	1	0
Kidney chromophobe	0	0	0	1	0
Kidney(clear cell)	0	0	0	1	0
Kidney (papillary cell)	0	0	0	2	0
Leukemia(AML)	0	0	0	0	0
Lower grade glioma	1	0	1	2	1
Liver	0	0	0	2	0
Lung adenocarcinoma	0	0	0	2	0
Lung(squamous cell)	2	1	1	0	0
Mesothelioma	0	0	0	0	0
Ovary	2	0	1	0	0
Pancreas	2	0	0	1	0
PCPG	3	0	0	0	0
Prostate	0	1	2	2	1
Rectum	0	0	0	1	0
Sarcoma	0	0	0	0	0
Skin(melanoma)	0	0	0	0	0
Stomach	2	0	1	2	0
Testicular	0	0	0	0	0
Thyroid	1	1	0	1	0
Thymoma	0	0	0	1	0
Endometrium	0	0	0	0	0
Uterine carcinosarcoma	0	0	0	0	0
Uveal melanoma	0	0	0	1	0

MAPK signaling pathway

	LOX	LOXL1	LOXL2	LOXL3	LOXL4
Adrenocortical	0	0	0	0	0
Bladder	0	1	2	1	0
Breast	1	0	1	0	0
Cervical	0	0	3	0	0
Cholangiocarcinoma	0	0	0	0	0
Colon	0	1	3	0	0
Lymphoma(DLBCL)	0	0	1	2	0
Esophagus	0	0	0	3	0
Glioblastoma multiforme	0	1	1	1	0
Head and neck	1	0	3	0	0
Kidney chromophobe	0	0	0	0	0
Kidney(clear cell)	0	0	1	1	0
Kidney (papillary cell)	0	0	0	0	0
Leukemia(AML)	0	0	0	0	0
Lower grade glioma	0	0	1	0	0
Liver	0	0	0	0	0
Lung adenocarcinoma	0	0	0	2	0
Lung(squamous cell)	0	2	3	3	0
Mesothelioma	0	0	0	0	0
Ovary	1	3	2	1	0
Pancreas	0	0	0	0	0
PCPG	0	0	0	0	0
Prostate	0	0	3	2	0
Rectum	0	1	0	0	0
Sarcoma	0	0	0	0	0
Skin(melanoma)	0	0	0	0	0
Stomach	1	2	1	3	0
Testicular	0	0	0	0	0
Thyroid	2	0	0	0	0
Thymoma	0	0	0	0	0
Endometrium	0	1	3	0	0
Uterine carcinosarcoma	0	0	0	0	0
Uveal melanoma	0	0	0	0	0

NF-kB Signaling Pathway

	LOX	LOXL1	LOXL2	LOXL3	LOXL4
Adrenocortical	0	0	0	0	0
Bladder	0	0	0	0	0
Breast	0	0	0	0	0
Cervical	0	0	0	0	0
Cholangiocarcinoma	0	0	0	0	0
Colon	0	0	1	0	0
Lymphoma(DLBCL)	0	0	0	0	0
Esophagus	0	0	1	0	0
Glioblastoma multiforme	1	1	2	1	0
Head and neck	0	0	0	0	0
Kidney chromophobe	0	0	0	0	0
Kidney(clear cell)	0	0	1	1	0
Kidney (papillary cell)	0	0	0	1	0
Leukemia(AML)	0	0	0	0	0
Lower grade glioma	0	0	3	1	0
Liver	0	0	0	1	0
Lung adenocarcinoma	0	0	0	0	0
Lung(squamous cell)	2	1	1	0	0
Mesothelioma	0	0	0	0	0
Ovary	1	1	0	0	1
Pancreas	3	0	0	2	0
PCPG	0	0	0	0	0
Prostate	0	0	1	1	0
Rectum	0	1	1	0	0
Sarcoma	0	0	0	0	0
Skin(melanoma)	0	0	0	0	0
Stomach	2	0	2	0	0
Testicular	0	0	0	0	0
Thyroid	2	1	0	1	0
Thymoma	0	0	0	1	0
Endometrium	1	0	0	0	0
Uterine carcinosarcoma	0	0	0	0	0
Uveal melanoma	0	0	0	0	0

PDGF signaling pathway

	LOX	LOXL1	LOXL2	LOXL3	LOXL4
Adrenocortical	0	0	1	0	0
Bladder	1	1	1	0	1
Breast	1	0	1	1	0
Cervical	0	1	1	1	0
Cholangiocarcinoma	0	0	0	0	0
Colon	1	1	1	1	1
Lymphoma(DLBCL)	1	0	0	2	0
Esophagus	1	1	1	1	0
Glioblastoma multiforme	1	1	2	2	1
Head and neck	1	1	1	0	0
Kidney chromophobe	0	0	1	0	0
Kidney(clear cell)	0	0	1	2	0
Kidney (papillary cell)	0	0	0	0	0
Leukemia(AML)	0	1	1	0	0
Lower grade glioma	1	1	1	1	0
Liver	0	0	1	1	0
Lung adenocarcinoma	1	1	1	1	0
Lung(squamous cell)	2	1	1	1	1
Mesothelioma	1	1	1	1	0
Ovary	2	1	1	0	2
Pancreas	5	0	0	4	0
PCPG	1	0	1	0	0
Prostate	0	1	1	1	1
Rectum	1	1	1	1	1
Sarcoma	0	0	0	0	0
Skin(melanoma)	1	1	1	0	0
Stomach	1	1	2	1	0
Testicular	1	1	1	1	1
Thyroid	1	0	1	0	0
Thymoma	1	1	1	1	1
Endometrium	1	1	1	0	1
Uterine carcinosarcoma	0	0	0	0	0
Uveal melanoma	0	0	0	0	0

FGF signaling pathway

	LOX	LOXL1	LOXL2	LOXL3	LOXL4
Adrenocortical	0	0	0	0	0
Bladder	8	5	14	16	0
Breast	0	0	1	12	0
Cervical	0	0	18	25	0
Cholangiocarcinoma	0	0	0	0	0
Colon	0	0	0	0	0
Lymphoma(DLBCL)	0	0	0	0	0
Esophagus	0	15	1	5	0
Glioblastoma multiforme	0	0	0	0	0
Head and neck	0	18	0	0	11
Kidney chromophobe	0	0	0	0	0
Kidney(clear cell)	0	0	0	0	0
Kidney (papillary cell)	0	0	0	0	0
Leukemia(AML)	0	0	0	0	0
Lower grade glioma	0	0	0	0	0
Liver	0	2	0	0	0
Lung adenocarcinoma	13	0	0	2	0
Lung(squamous cell)	5	6	0	34	0
Mesothelioma	1	0	0	0	0
Ovary	0	20	0	13	0
Pancreas	0	0	0	0	0
PCPG	0	0	0	0	0
Prostate	0	7	8	2	5
Rectum	3	0	0	0	0
Sarcoma	0	0	0	0	0
Skin(melanoma)	9	0	0	0	0
Stomach	0	3	0	6	0
Testicular	11	1	0	7	12
Thyroid	0	3	0	0	0
Thymoma	3	2	10	8	3
Endometrium	0	13	30	0	6
Uterine carcinosarcoma	0	0	0	0	0
Uveal melanoma	0	0	0	0	0

NOTCH signaling pathway

	LOX	LOXL1	LOXL2	LOXL3	LOXL4
Adrenocortical	0	0	0	0	0
Bladder	0	0	0	0	0
Breast	0	0	0	0	0
Cervical	0	0	0	0	0
Cholangiocarcinoma	0	0	0	0	0
Colon	0	0	0	0	0
Lymphoma(DLBCL)	0	0	0	0	0
Esophagus	0	0	0	0	0
Glioblastoma multiforme	0	0	0	0	0
Head and neck	0	0	3	0	0
Kidney chromophobe	0	0	0	0	0
Kidney(clear cell)	0	0	7	2	0
Kidney (papillary cell)	0	0	0	0	0
Leukemia(AML)	0	0	0	0	0
Lower grade glioma	0	0	3	1	0
Liver	0	0	0	0	0
Lung adenocarcinoma	2	0	1	0	0
Lung(squamous cell)	0	0	0	0	0
Mesothelioma	0	0	0	0	0
Ovary	0	0	0	0	0
Pancreas	0	0	0	0	0
PCPG	0	0	3	0	0
Prostate	0	0	0	0	0
Rectum	0	0	0	0	0
Sarcoma	0	0	0	0	0
Skin(melanoma)	0	0	0	0	0
Stomach	0	0	1	0	0
Testicular	1	0	0	1	0
Thyroid	0	0	0	0	0
Thymoma	0	0	0	1	0
Endometrium	0	2	2	1	0
Uterine carcinosarcoma	0	0	0	0	0
Uveal melanoma	0	0	0	0	0

VEGF signaling pathway

	LOX	LOXL1	LOXL2	LOXL3	LOXL4
Adrenocortical	0	0	1	0	0
Bladder	1	1	2	0	0
Breast	3	0	2	0	0
Cervical	0	0	1	0	0
Cholangiocarcinoma	0	0	0	0	0
Colon	0	1	3	0	0
Lymphoma(DLBCL)	0	0	0	1	0
Esophagus	0	0	2	0	0
Glioblastoma multiforme	2	3	4	3	1
Head and neck	1	0	4	0	0
Kidney chromophobe	0	0	0	0	0
Kidney(clear cell)	0	0	2	1	0
Kidney (papillary cell)	0	0	0	0	0
Leukemia(AML)	0	0	0	0	0
Lower grade glioma	0	0	2	0	0
Liver	0	0	0	0	0
Lung adenocarcinoma	3	1	2	0	0
Lung(squamous cell)	2	1	3	1	1
Mesothelioma	1	1	1	0	0
Ovary	3	2	2	0	3
Pancreas	4	0	0	2	0
PCPG	2	0	1	0	0
Prostate	0	0	3	0	0
Rectum	0	0	0	0	0
Sarcoma	0	0	0	0	0
Skin(melanoma)	0	0	0	0	0
Stomach	3	1	6	1	0
Testicular	0	0	0	0	0
Thyroid	0	0	0	0	0
Thymoma	0	0	1	1	0
Endometrium	1	0	2	0	0
Uterine carcinosarcoma	0	0	0	0	0
Uveal melanoma	0	0	0	0	0

Figure S7. Count summary of activated pathways enriched by the Gene Set Enrichment Analysis (GSEA) in case of LOX/LOXL overexpression, including Wnt, GPCRs, transforming growth factor-β (TGF-β), PI3Ks, MET, JAK/STAT, mitogen-activated protein kinases (MAPKs), NF-kB, platelet-derived growth factor (PDGF), fibroblast growth factor (FGF), NOTCH and vascular endothelial growth factor (VEGF）.

Figure S8

A

Cell cycle and chromosome

	LOX	LOXL1	LOXL2	LOXL3	LOXL4
Adrenocortical	0	0	10	0	0
Bladder	0	0	0	0	0
Breast	0	0	0	0	0
Cervical	0	0	0	0	0
Cholangiocarcinoma	0	0	0	0	0
Colon	2	0	0	0	0
Lymphoma(DLBCL)	0	0	0	0	0
Esophagus	0	0	0	0	0
Glioblastoma multiforme	0	0	1	0	0
Head and neck	0	0	0	0	0
Kidney chromophobe	15	0	0	0	0
Kidney(clear cell)	0	0	8	3	0
Kidney (papillary cell)	0	0	0	0	0
Leukemia(AML)	0	0	0	0	0
Lower grade glioma	15	0	71	16	0
Liver	0	0	0	0	0
Lung adenocarcinoma	35	0	41	0	0
Lung(squamous cell)	0	0	0	0	0
Mesothelioma	14	5	17	0	0
Ovary	0	0	0	0	0
Pancreas	0	0	0	0	0
PCPG	0	0	0	0	0
Prostate	0	0	0	0	0
Rectum	0	0	0	0	0
Sarcoma	0	0	5	0	0
Skin(melanoma)	0	0	0	0	0
Stomach	0	0	0	0	0
Testicular	0	0	0	0	0
Thyroid	3	0	0	0	0
Thymoma	0	0	0	0	0
Endometrium	1	0	0	21	0
Uterine carcinosarcoma	0	0	0	0	0
Uveal melanoma	13	0	0	0	0

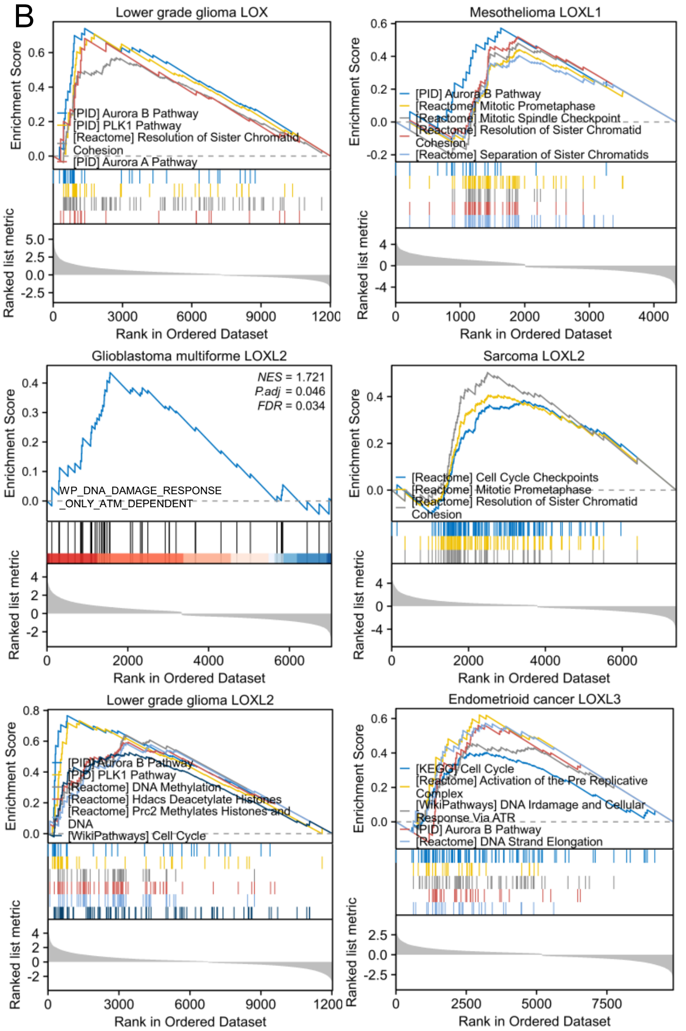

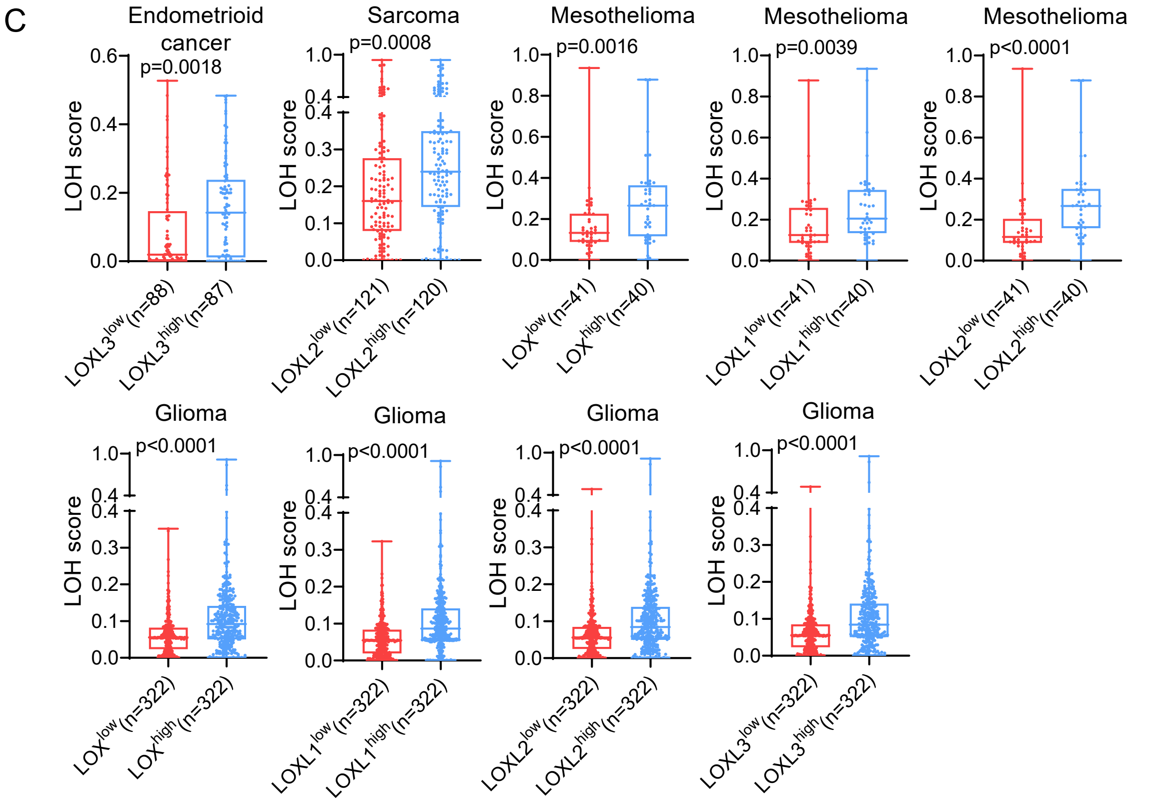

Figure S8. Enrichment of activated pathways involving Cell cycle and chromosome. (A) Count summary of activated pathways in pan cancer. (B) Examples showing the activated pathways enriched by the Gene Set Enrichment Analysis (GSEA) in case of LOX/LOXL overexpression. (C) The score of loss of heterozygosity (LOH) was compared between LOX/LOXL high and low groups in cancer. Wilcoxon rank sum test was performed for statistical analysis. P-value < 0.05 is considered statistically significant.

Figure S9

A

LOX/LOXL	Correlated genes	rvalue	pvalue
LOX	ADPGK	0.73	1.193E-114
LOX	AK2	0.76	1.947E-131
LOX	CAPZA1	0.80	1.931E-154
LOX	CASP4	0.73	3.21E-114
LOX	CLIC1	0.74	3.209E-119
LOX	COL1A1	0.72	4.541E-108
LOX	COL4A2	0.72	1.2E-111
LOX	EMP3	0.71	4.82E-104
LOX	FAM114A1	0.72	6.65E-109
LOX	FNDC3B	0.75	1.988E-122
LOX	GLA	0.71	4.58E-107
LOX	MSN	0.74	6.553E-118
LOX	MYL12A	0.72	2.645E-111
LOX	P3H1	0.73	9.086E-114
LOX	PLEK2	0.73	8.751E-113
LOX	PYGL	0.73	1.167E-115
LOX	RER1	0.78	1.209E-138
LOX	S100A11	0.72	1.969E-110
LOX	SDF4	0.71	6.529E-106
LOX	SERPINH1	0.74	2.178E-120
LOX	VASP	0.73	3.528E-115
LOXL1	COL1A1	0.71	4.906E-105
LOXL1	COL4A2	0.70	1.724E-101
LOXL1	EMP3	0.77	3.905E-133
LOXL1	FAM114A1	0.72	7.448E-109
LOXL1	MYL12A	0.72	7.448E-109
LOXL1	PLEK2	0.71	1.065E-103
LOXL1	SERPINH1	0.73	2.619E-113
LOXL2	ADPGK	0.71	6.841E-106
LOXL2	AK2	0.72	1.095E-113
LOXL2	CAPZA1	0.72	0
LOXL2	CASP4	0.74	2.209E-119
LOXL2	CLIC1	0.73	0
LOXL2	COL1A1	0.71	5.787E-106
LOXL2	COL4A2	0.80	0
LOXL2	FNDC3B	0.74	1.973E-119
LOXL2	GLA	0.71	4.917E-108
LOXL2	P3H1	0.76	1.677E-130
LOXL2	PLEK2	0.73	5.804E-116
LOXL2	PYGL	0.76	3.834E-131
LOXL2	RER1	0.70	0
LOXL2	S100A11	0.72	0
LOXL2	SDF4	0.71	4.234E-108
LOXL2	SERPINH1	0.78	0
LOXL2	VASP	0.77	7.893E-136
LOXL3	ADPGK	0.75	1.683E-122
LOXL3	AK2	0.71	2.27E-104
LOXL3	CAPZA1	0.71	7.058E-106
LOXL3	CASP4	0.71	9.761E-106
LOXL3	CLIC1	0.70	4.591E-103
LOXL3	FAM114A1	0.73	1.133E-113
LOXL3	FNDC3B	0.72	5.517E-108
LOXL3	GLA	0.70	2.472E-102
LOXL3	MSN	0.71	9.772E-105
LOXL3	P3H1	0.71	2.456E-106
LOXL3	PYGL	0.75	1.313E-121
LOXL3	RER1	0.71	1.596E-104
LOXL3	S100A11	0.73	3.045E-112
LOXL3	SDF4	0.72	1.704E-110
LOXL3	VASP	0.71	6.618E-105
LOXL4	EMP3	0.76	8.077E-129
LOXL4	FAM114A1	0.74	1.761E-120
LOXL4	MSN	0.76	1.083E-130
LOXL4	MYL12A	0.70	6.011E-102

B

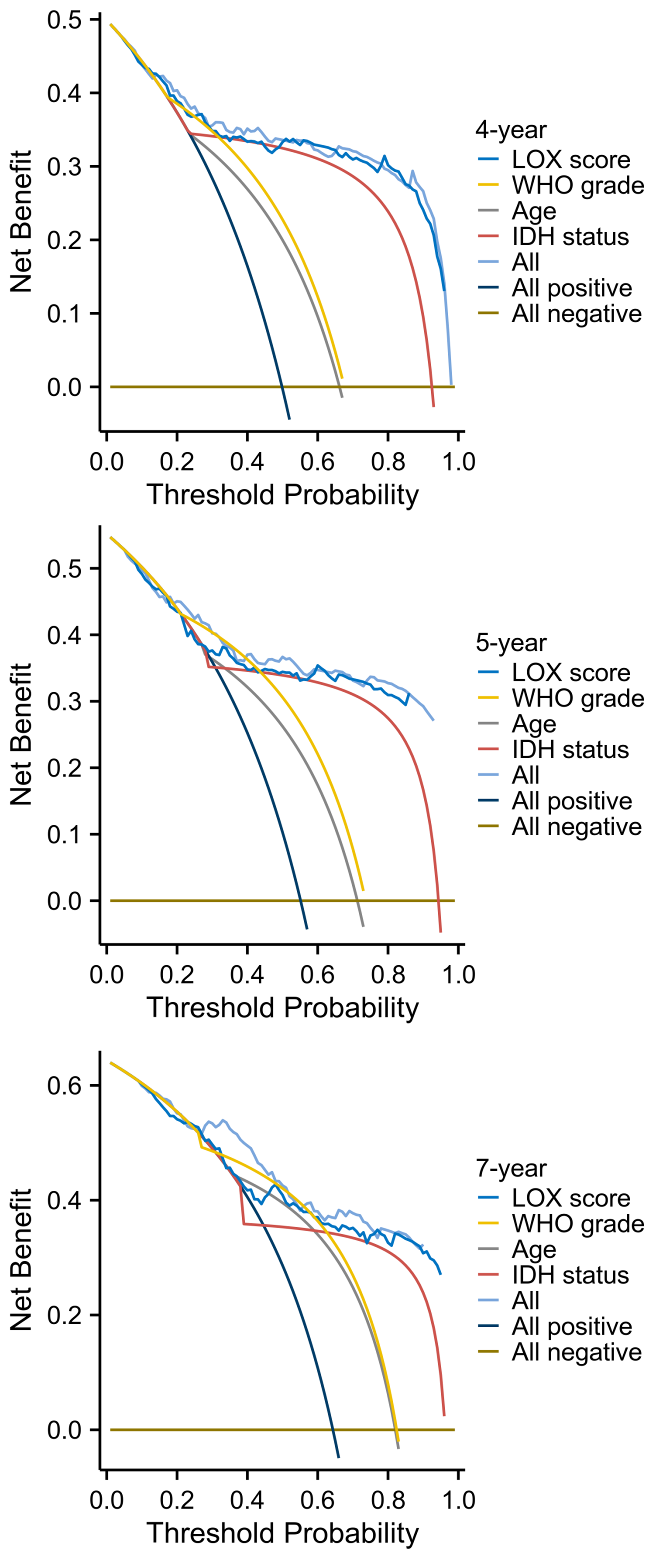

Figure S9. (A) The summary of the genes co-expressed with ≥ three of the five LOX/LOXL genes. Spearman's correlation coefficients between LOX/LOXL gene expression and mRNA expression in TCGA glioma were calculated. P-value < 0.05 is considered statistically significant. (B) DCA curves in TCGA cohort.

Figure S10

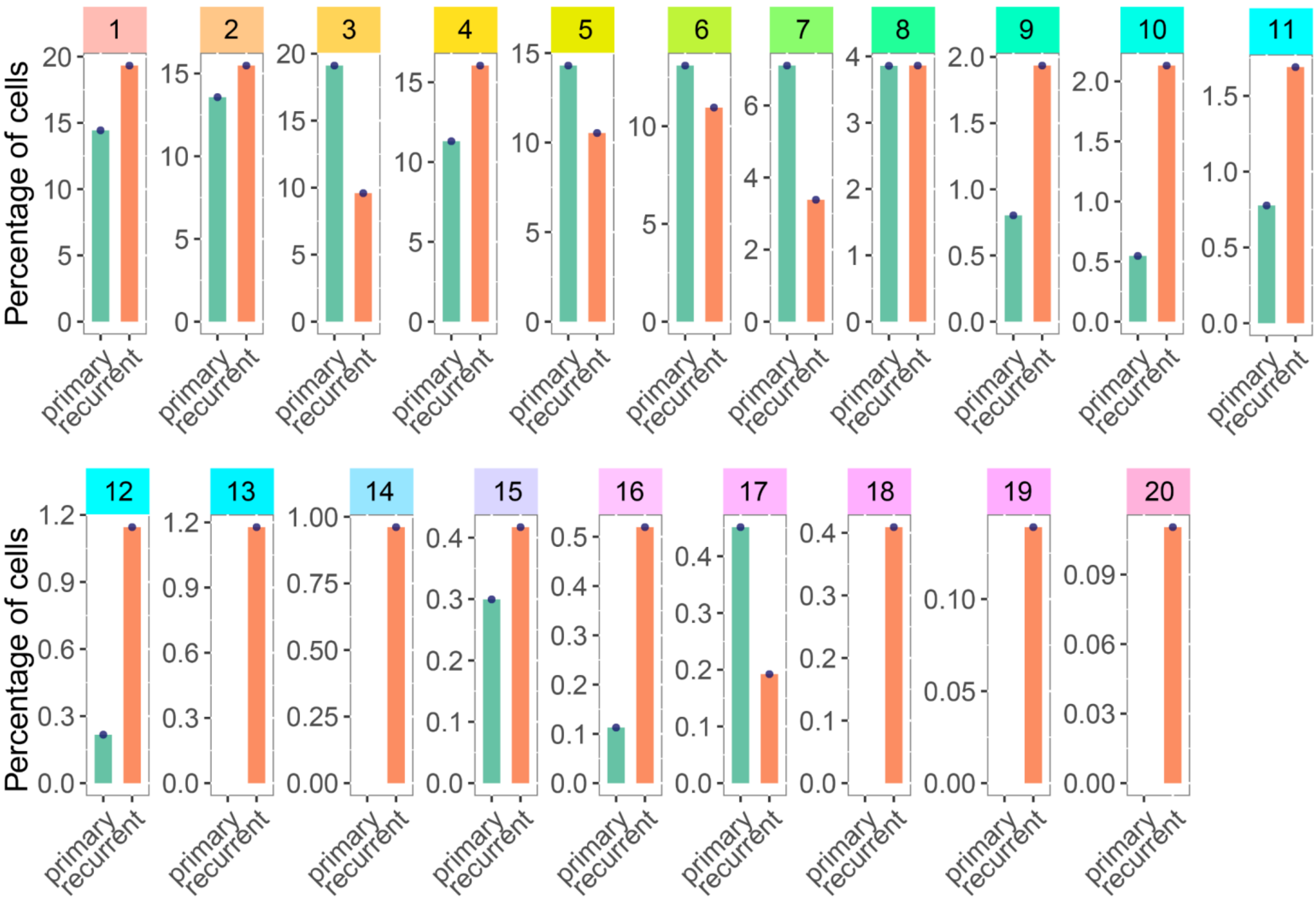

1. Oligodendrocyte progenitor cell 1
2. Microglial cell
3. Oligodendrocyte progenitor cell 2
4. Oligodendrocyte 1
5. Astrocyte 1
6. PDGFRA Oligodendrocyte progenitor cell
7. SLC17A7 Glutamatergic neuron
8. Neural progenitor cell
9. Glutamatergic and GABAergic neuron
10. Endothelial cell 1
11. GABAergic neuron 1
12. Astrocyte cancer stem cell
13. Astrocyte 2
14. **Mesenchymal cell**
15. NKT
16. CD163 Macrophage
17. Oligodendrocyte 2
18. GABAergic neuron 2
19. Astrocyte 3
20. Schwann cell

Figure S10. Percentages of cell types in primary and recurrent tumor samples as assessed by single cell sequencing analysis from samples of n=7 patients with IDH wild-type glioblastoma multiforme.

Figure S11

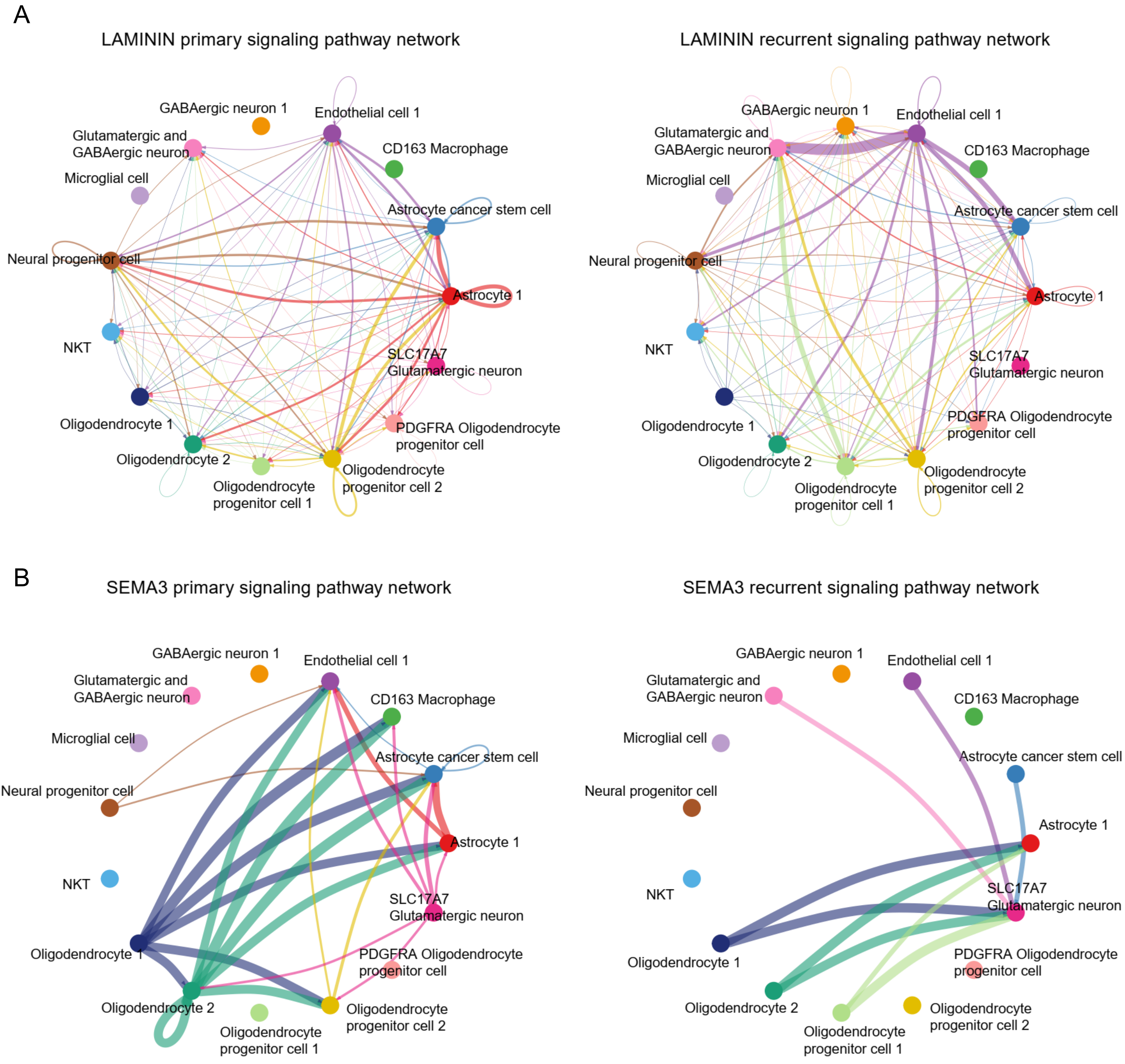

Figure S11. The comparison of cell-cell communication network based on laminin (A) and Class 3 semaphorin protein (SEMA3) (B) pathways between primary and recurrent cell populations. In the circle plots, edge colors are consistent with the sources as sender, and edge weights are proportional to the interaction strength. Thicker edge line indicates a stronger signal.

Figure S12

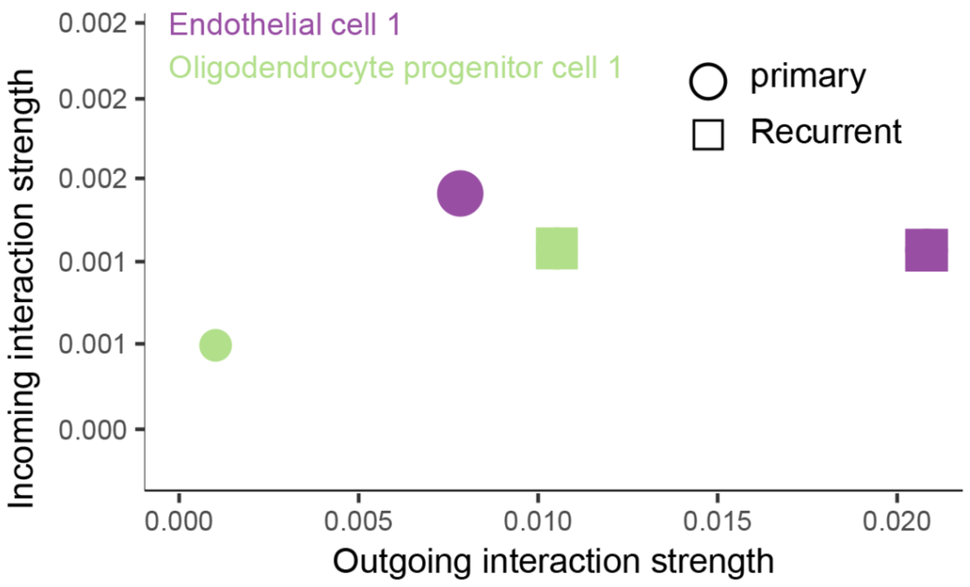

Figure S12. Comparison of the major signal sources and targets in 2D space between primary and recurrent cell populations. The scatter plot demonstrated that both oligodendrocyte progenitor 1 and endothelial cell 1 populations were the prominent sources of signals in recurrence state compared to primary status.

Figure S13

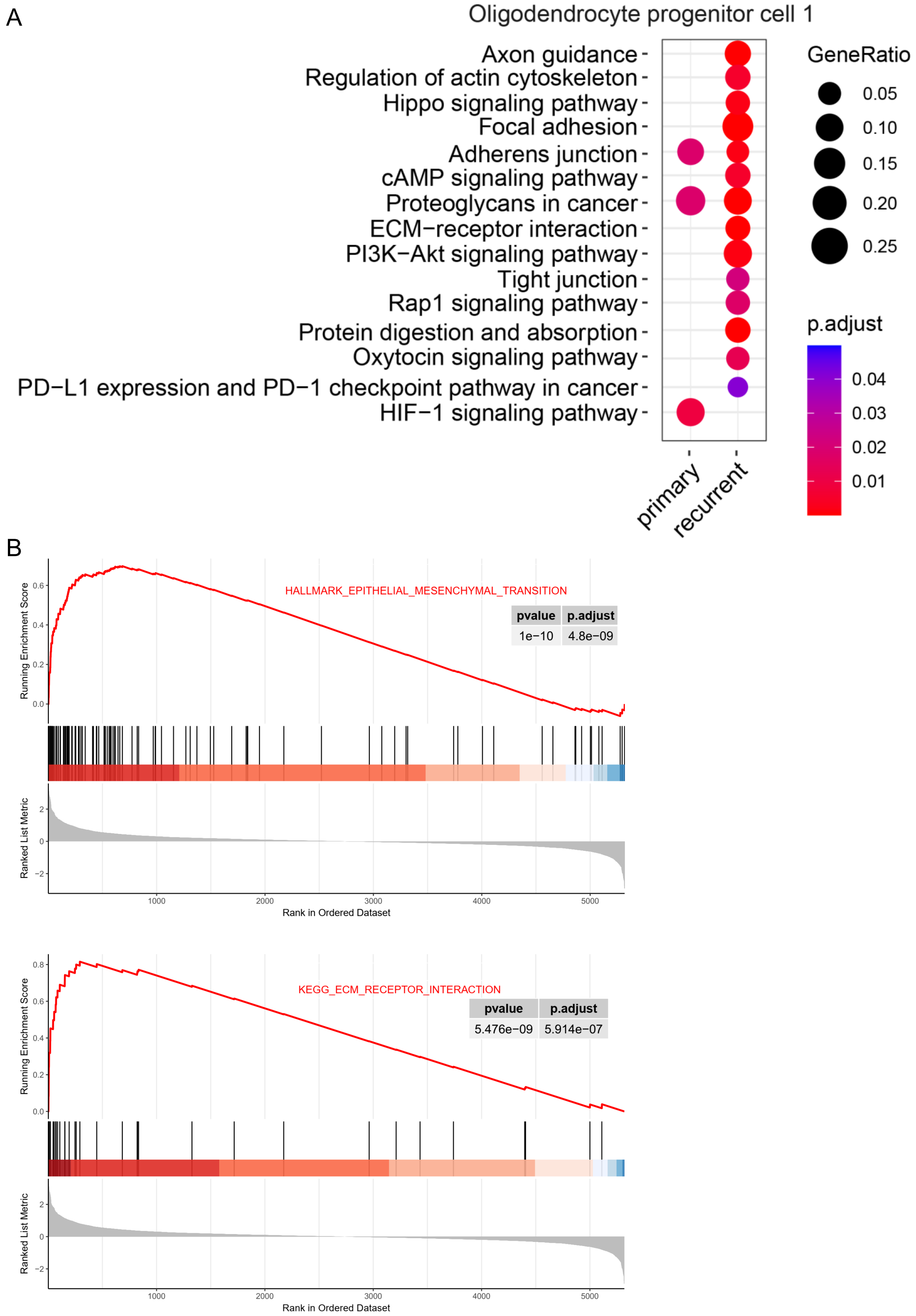

Figure S13. Pathway enrichment of genes upregulated in recurrent oligodendrocyte progenitor cell 1 compared with primary status. Both KEGG pathway analyses (A) and GSEA (B) were performed. GeneRatio indicates the enrichment level of that category within the gene set.

Figure S14

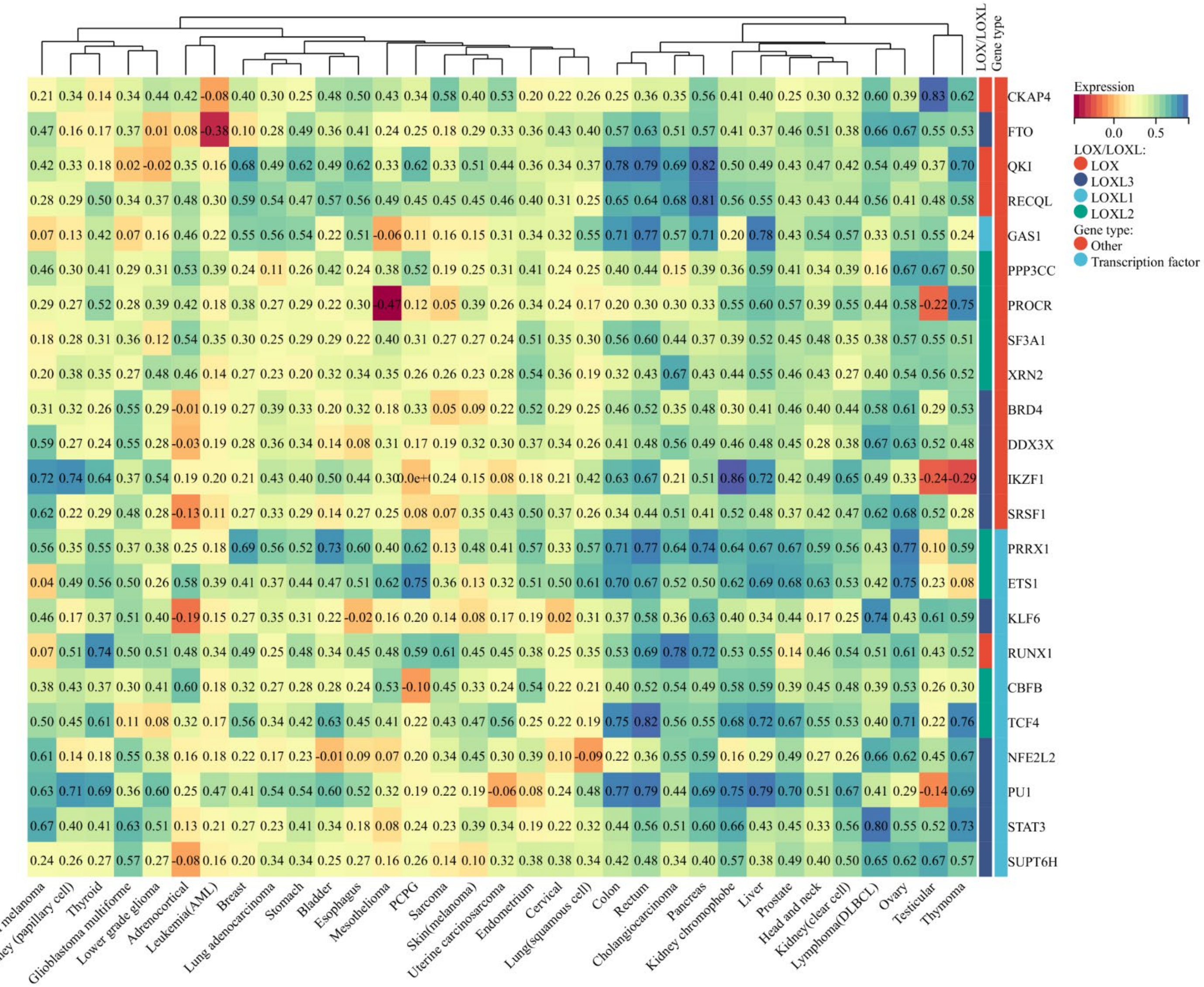

Figure S14. Heatmap of the spearman correlation coefficient in pan cancer between mRNA expression of LOX/LOXL genes and their upstream genes identified by the genetic perturbation similarity analysis database (GPSAdb). In the heatmap each column represents a cancer type and each row is a gene regulating LOX/LOXL expression. Blue color indicates stronger positive spearman correlation coefficient and red color indicates stronger negative spearman correlation coefficient.

Figure S15

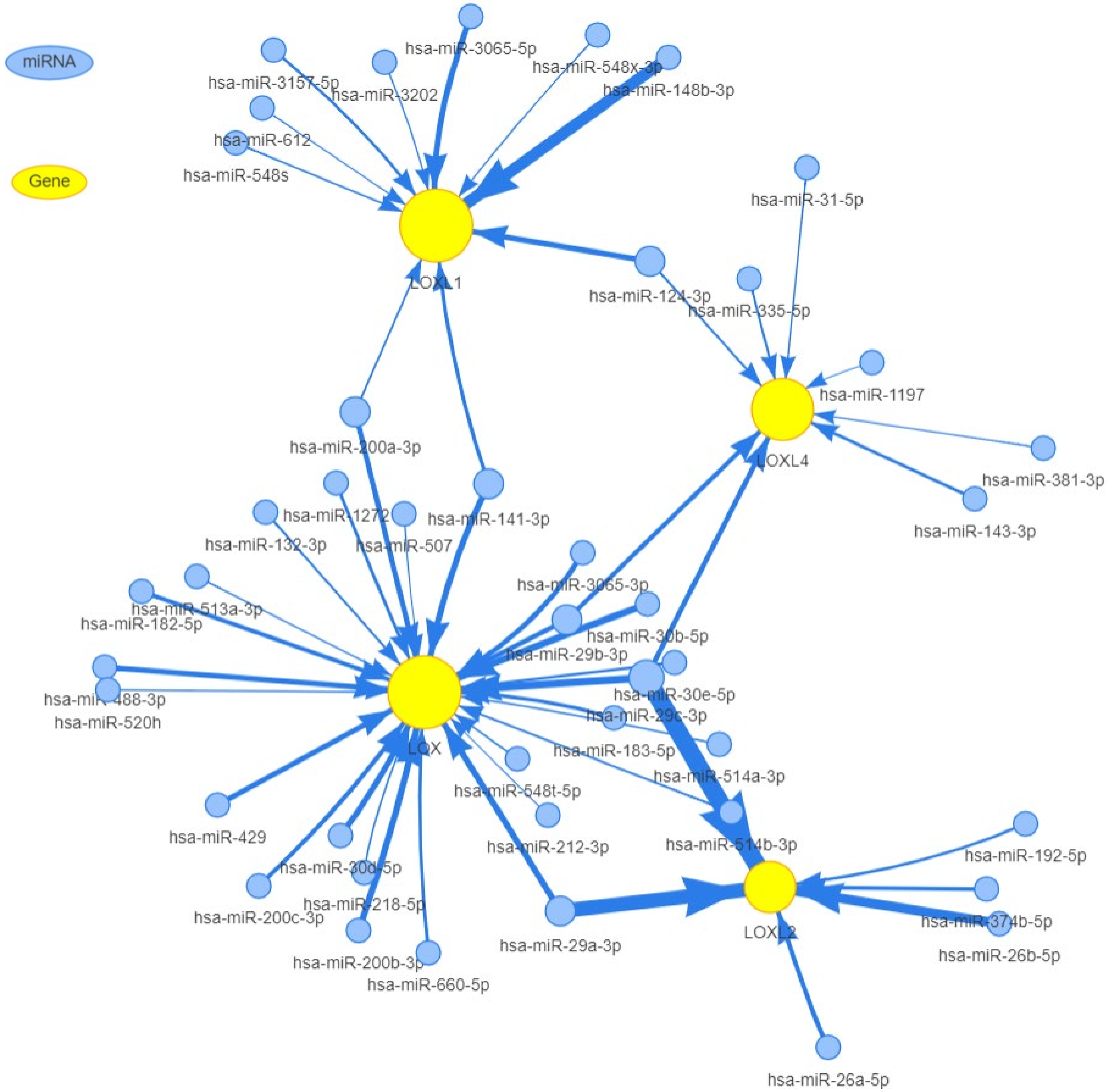

Figure S15. The microRNA (miRNA) network of LOX/LOXL regulators. An miRNA and one regulator connection node represent miRNA regulation of a gene. Edge width is defined by the absolute value of the correlation coefficient.

Figure S16

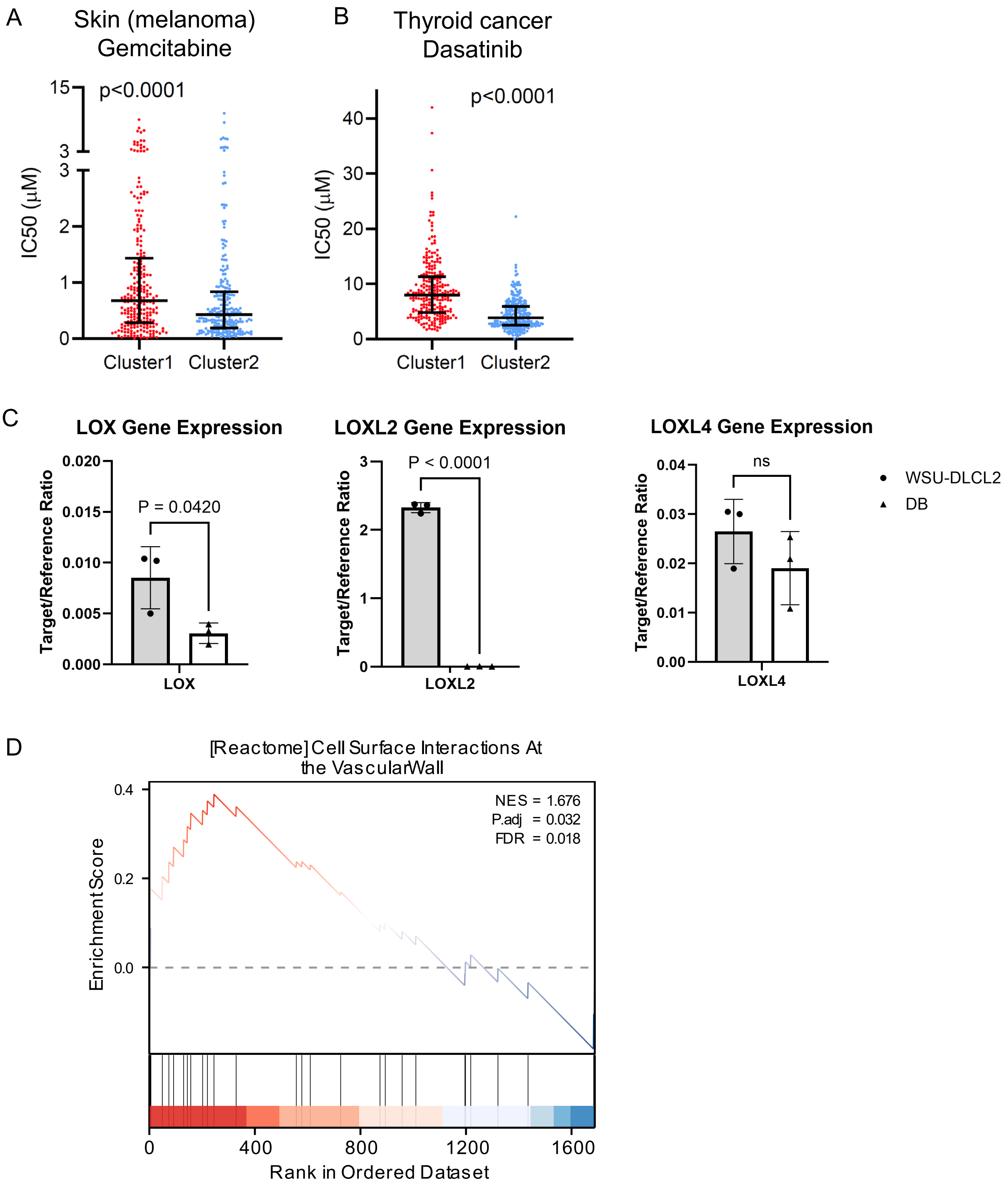

Figure S16. (A-B) IC50 values of gemcitabine (A) and dasatinib (B) were compared between pan-LOX/LOXL-high and -low group in TCGA skin cutaneous melanoma and thyroid cancer, respectively. (C) The expression of *LOX*, *LOXL2*, and *LOXL4* genes in DB and WSU-DLBCL2 cell lines was tested by quantitative reverse transcription PCR (RT-qPCR); median from n=3 technical replicates±SD; Statistical significance was analyzed using Two-sided unpaired Student's t test. $P < 0.05$ indicated statistical significance; (D) Pathway enrichment of genes upregulated in LOX/LOXL-high DLBCL TCGA cohort compared with -low cohort. GSEA were performed, indicating the activated pathway of cell surface interactions at the vascular wall.

Figure S17

T cell

	LOX	LOXL1	LOXL2	LOXL3	LOXL4
Adrenocortical	0	5	0	2	0
Bladder	10	0	7	18	0
Breast	0	0	0	3	1
Cervical	0	0	0	0	0
Cholangiocarcinoma	0	1	0	0	0
Colon	2	0	3	2	0
Lymphoma(DLBCL)	0	0	0	2	0
Esophagus	0	1	1	2	0
Glioblastoma multiforme	3	4	6	7	3
Head and neck	0	0	0	16	0
Kidney chromophobe	0	0	0	16	0
Kidney(clear cell)	0	0	1	21	0
Kidney (papillary cell)	0	0	0	20	0
Leukemia(AML)	0	0	0	0	0
Lower grade glioma	7	4	17	16	0
Liver	0	0	0	14	0
Lung adenocarcinoma	2	3	0	15	0
Lung(squamous cell)	2	0	2	8	0
Mesothelioma	0	0	8	2	0
Ovary	16	2	0	0	3
Pancreas	12	0	0	8	0
PCPG	4	0	0	0	0
Prostate	0	8	11	21	1
Rectum	1	1	0	0	0
Sarcoma	0	0	0	0	0
Skin(melanoma)	0	6	0	0	0
Stomach	3	0	1	11	0
Testicular	0	0	0	0	0
Thyroid	14	13	0	20	0
Thymoma	0	0	0	2	0
Endometrium	0	0	0	0	0
Uterine carcinosarcoma	0	0	0	0	0
Uveal melanoma	0	0	0	18	11

Granulocyte and neutrophil

	LOX	LOXL1	LOXL2	LOXL3	LOXL4
Adrenocortical	0	1	1	1	0
Bladder	2	1	2	1	0
Breast	1	0	0	1	0
Cervical	0	0	0	0	0
Cholangiocarcinoma	0	0	0	0	0
Colon	2	3	3	2	1
Lymphoma(DLBCL)	0	0	0	1	0
Esophagus	0	0	1	1	0
Glioblastoma multiforme	1	2	2	2	1
Head and neck	1	1	2	3	0
Kidney chromophobe	0	0	0	3	0
Kidney(clear cell)	0	0	1	2	0
Kidney (papillary cell)	0	0	0	3	0
Leukemia(AML)	0	0	0	0	0
Lower grade glioma	1	0	1	2	1
Liver	0	2	0	2	0
Lung adenocarcinoma	0	1	0	2	0
Lung(squamous cell)	3	1	2	1	0
Mesothelioma	0	0	0	0	0
Ovary	3	0	2	0	2
Pancreas	3	0	0	1	0
PCPG	2	0	1	0	0
Prostate	0	2	3	3	2
Rectum	2	1	1	2	0
Sarcoma	0	0	0	0	0
Skin(melanoma)	0	1	0	0	0
Stomach	3	1	2	3	0
Testicular	0	0	0	0	0
Thyroid	3	3	0	3	0
Thymoma	0	0	0	1	0
Endometrium	0	0	0	0	0
Uterine carcinosarcoma	0	0	0	0	0
Uveal melanoma	0	0	0	0	0

DC and macrophage

	LOX	LOXL1	LOXL2	LOXL3	LOXL4
Adrenocortical	0	0	1	0	0
Bladder	2	1	1	3	1
Breast	1	0	0	0	0
Cervical	0	0	1	0	0
Cholangiocarcinoma	0	0	0	0	0
Colon	1	1	1	1	1
Lymphoma(DLBCL)	0	0	0	1	0
Esophagus	0	0	1	1	1
Glioblastoma multiforme	1	1	1	1	1
Head and neck	1	1	1	1	0
Kidney chromophobe	0	0	1	1	0
Kidney(clear cell)	0	0	1	1	0
Kidney (papillary cell)	0	0	0	3	0
Leukemia(AML)	0	0	1	0	0
Lower grade glioma	1	0	2	2	1
Liver	0	0	0	0	0
Lung adenocarcinoma	0	0	0	1	0
Lung(squamous cell)	1	0	2	1	0
Mesothelioma	0	0	0	0	0
Ovary	1	1	1	0	1
Pancreas	1	0	0	1	0
PCPG	1	0	1	0	0
Prostate	0	1	1	1	0
Rectum	1	0	1	0	1
Sarcoma	0	0	0	0	0
Skin(melanoma)	0	0	0	0	0
Stomach	1	0	1	1	0
Testicular	1	0	0	1	1
Thyroid	0	1	0	2	0
Thymoma	0	0	1	0	0
Endometrium	0	0	0	0	0
Uterine carcinosarcoma	0	0	0	0	0
Uveal melanoma	0	0	0	0	0

NK cell

	LOX	LOXL1	LOXL2	LOXL3	LOXL4
Adrenocortical	0	1	0	0	0
Bladder	1	0	1	1	0
Breast	0	0	0	1	0
Cervical	0	0	0	0	0
Cholangiocarcinoma	0	0	0	0	0
Colon	0	1	1	0	0
Lymphoma(DLBCL)	0	0	0	1	0
Esophagus	0	0	0	0	0
Glioblastoma multiforme	0	0	1	0	0
Head and neck	0	0	0	2	0
Kidney chromophobe	0	0	0	2	0
Kidney(clear cell)	0	0	0	3	0
Kidney (papillary cell)	0	0	0	2	0
Leukemia(AML)	0	0	0	0	0
Lower grade glioma	0	1	1	1	0
Liver	0	0	0	1	0
Lung adenocarcinoma	1	0	1	2	0
Lung(squamous cell)	1	0	1	0	0
Mesothelioma	0	0	0	1	0
Ovary	2	0	0	0	0
Pancreas	1	0	0	1	0
PCPG	0	0	0	0	0
Prostate	0	1	1	2	0
Rectum	0	0	0	0	0
Sarcoma	0	0	0	0	0
Skin(melanoma)	0	0	0	0	0
Stomach	1	0	1	2	0
Testicular	0	0	0	0	0
Thyroid	3	2	0	2	0
Thymoma	0	0	0	0	0
Endometrium	0	0	0	0	0
Uterine carcinosarcoma	0	0	0	0	0
Uveal melanoma	0	0	0	2	1

B cell

	LOX	LOXL1	LOXL2	LOXL3	LOXL4
Adrenocortical	0	0	0	0	0
Bladder	2	0	1	2	0
Breast	0	0	0	0	0
Cervical	0	0	0	0	0
Cholangiocarcinoma	0	0	0	0	0
Colon	1	1	1	1	0
Lymphoma(DLBCL)	0	0	0	0	0
Esophagus	0	0	0	0	0
Glioblastoma multiforme	0	0	2	0	0
Head and neck	0	0	0	4	0
Kidney chromophobe	0	0	0	4	0
Kidney(clear cell)	0	0	0	6	0
Kidney (papillary cell)	0	0	0	6	0
Leukemia(AML)	0	0	0	0	0
Lower grade glioma	1	0	2	2	0
Liver	0	0	0	3	0
Lung adenocarcinoma	0	0	0	4	0
Lung(squamous cell)	1	0	0	1	0
Mesothelioma	0	0	0	0	0
Ovary	1	0	0	0	0
Pancreas	5	0	0	1	0
PCPG	1	0	0	0	0
Prostate	0	2	2	8	0
Rectum	0	0	0	0	0
Sarcoma	0	0	0	0	0
Skin(melanoma)	0	0	0	0	0
Stomach	0	0	0	1	0
Testicular	0	0	0	0	0
Thyroid	3	1	0	6	0
Thymoma	0	0	0	0	0
Endometrium	0	0	0	0	0
Uterine carcinosarcoma	0	0	0	0	0
Uveal melanoma	0	0	0	0	0

Mast cell

	LOX	LOXL1	LOXL2	LOXL3	LOXL4
Adrenocortical	0	0	0	0	0
Bladder	0	0	0	0	0
Breast	1	0	0	0	0
Cervical	0	0	0	0	0
Cholangiocarcinoma	0	0	0	0	0
Colon	0	0	0	0	0
Lymphoma(DLBCL)	0	0	0	1	0
Esophagus	0	0	0	0	0
Glioblastoma multiforme	0	0	0	0	1
Head and neck	0	0	0	3	0
Kidney chromophobe	0	0	0	3	0
Kidney(clear cell)	0	0	0	3	0
Kidney (papillary cell)	0	0	0	3	0
Leukemia(AML)	0	0	0	0	0
Lower grade glioma	0	0	1	1	0
Liver	0	0	0	0	0
Lung adenocarcinoma	0	0	0	0	0
Lung(squamous cell)	1	0	0	0	0
Mesothelioma	0	0	0	0	0
Ovary	1	0	0	0	2
Pancreas	3	0	0	2	0
PCPG	0	0	0	0	0
Prostate	0	0	1	2	0
Rectum	0	0	0	0	0
Sarcoma	0	0	0	0	0
Skin(melanoma)	0	0	0	0	0
Stomach	0	0	0	0	0
Testicular	0	0	0	0	0
Thyroid	2	0	0	3	0
Thymoma	0	0	0	0	0
Endometrium	0	0	0	0	0
Uterine carcinosarcoma	0	0	0	0	0
Uveal melanoma	0	0	0	0	0

Monocyte

	LOX	LOXL1	LOXL2	LOXL3	LOXL4
Adrenocortical	0	0	0	0	0
Bladder	0	0	0	0	0
Breast	0	0	0	0	0
Cervical	0	0	0	0	0
Cholangiocarcinoma	0	0	0	0	0
Colon	1	1	1	0	0
Lymphoma(DLBCL)	0	0	0	0	0
Esophagus	0	0	0	0	0
Glioblastoma multiforme	0	0	0	0	0
Head and neck	1	0	0	0	0
Kidney chromophobe	0	0	0	0	0
Kidney(clear cell)	0	0	0	1	0
Kidney (papillary cell)	0	0	0	1	0
Leukemia(AML)	0	0	0	0	0
Lower grade glioma	0	0	1	0	0
Liver	0	0	0	0	0
Lung adenocarcinoma	0	0	0	0	0
Lung(squamous cell)	0	0	0	1	0
Mesothelioma	0	0	0	0	0
Ovary	1	0	1	0	0
Pancreas	1	0	0	0	0
PCPG	1	0	0	0	0
Prostate	0	0	1	1	0
Rectum	0	0	0	0	0
Sarcoma	0	0	0	0	0
Skin(melanoma)	0	0	0	0	0
Stomach	0	0	0	1	0
Testicular	0	0	0	0	0
Thyroid	1	0	0	1	0
Thymoma	0	0	0	0	0
Endometrium	0	0	0	0	0
Uterine carcinosarcoma	0	0	0	0	0
Uveal melanoma	0	0	0	0	0

Figure S17. Count summary of activated immune-related pathways enriched by the Gene Set Enrichment Analysis (GSEA) in case of LOX/LOXL overexpression, including pathways involving T cell, granulocyte/neutrophil, dendritic cell (DC), macrophage, natural killer (NK) cell, B cell, mast cell and monocyte.

Figure S18

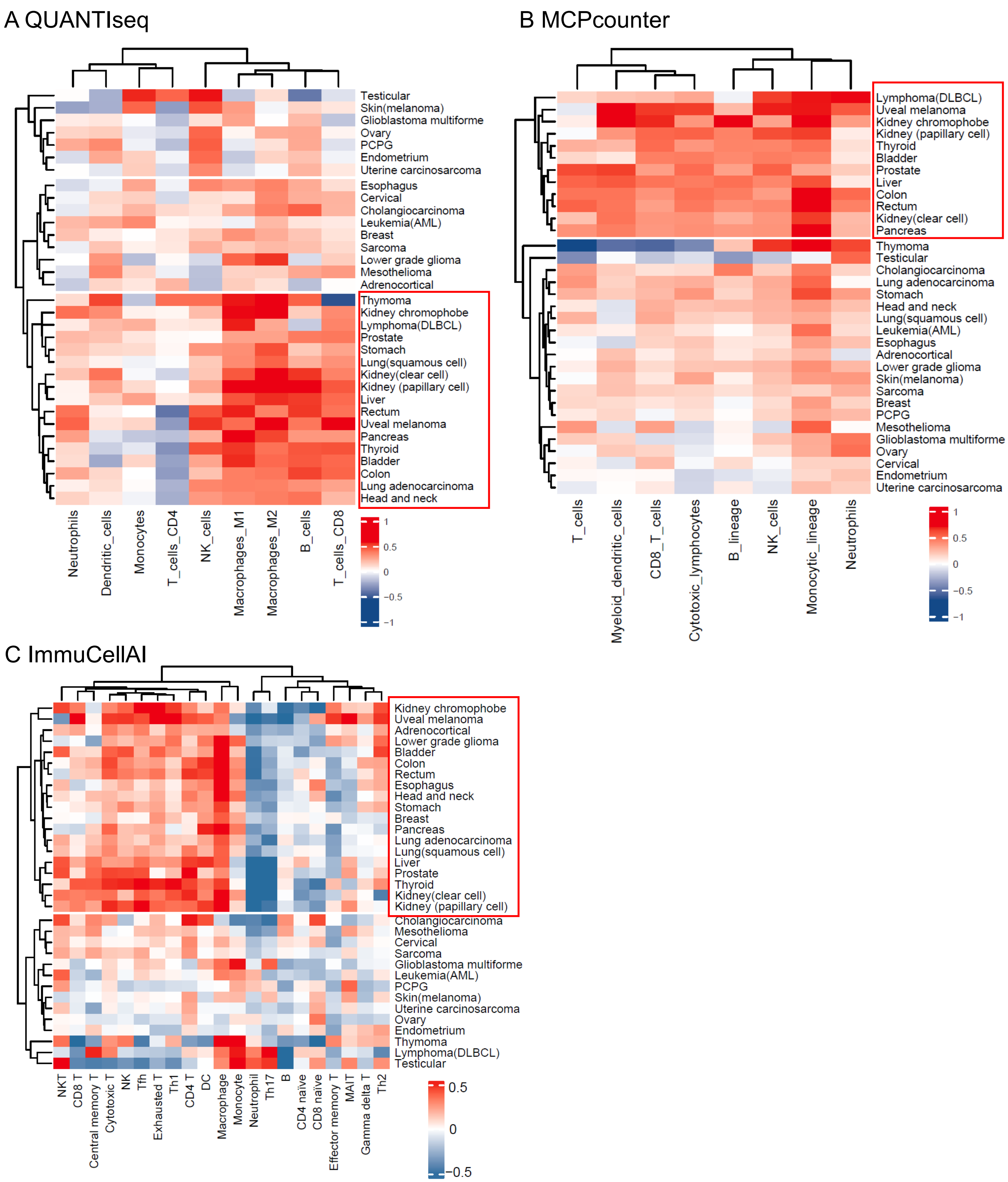

Figure S18. Heatmap of the spearman correlation coefficient between *LOXL3* expression and the infiltration abundance of immune cell types in 33 malignancies. The infiltration abundance was calculated by QUANTIseq (A), MCPcounter (B), and ImmuCellAI (C). The unsupervised consensus clustering in the heatmap reveals a distinct cluster showing malignancies displaying positive correlation, regarded as immunologically hot cancers. Red colour indicates positive correlation, and blue colour indicates negative correlation.

Figure S19

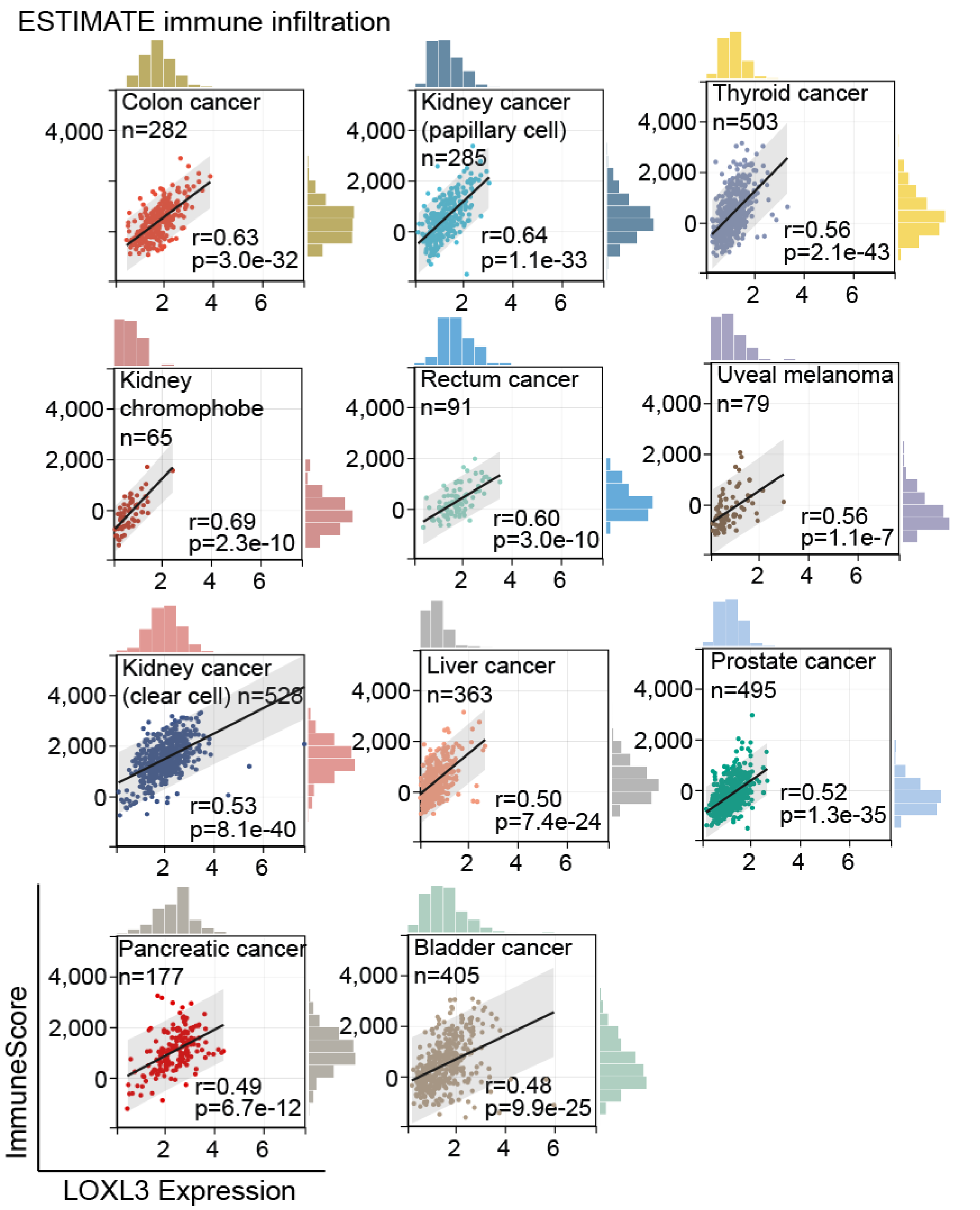

Figure S19. Correlation between LOX/LOXL expression and immune infiltration. The immune infiltration score was calculated by the R package ESTIMATE (v.1.0.13) and correlated with LOX/LOXL expression by the spearman correlation analysis. P-value < 0.05 is considered statistically significant.

Figure S20

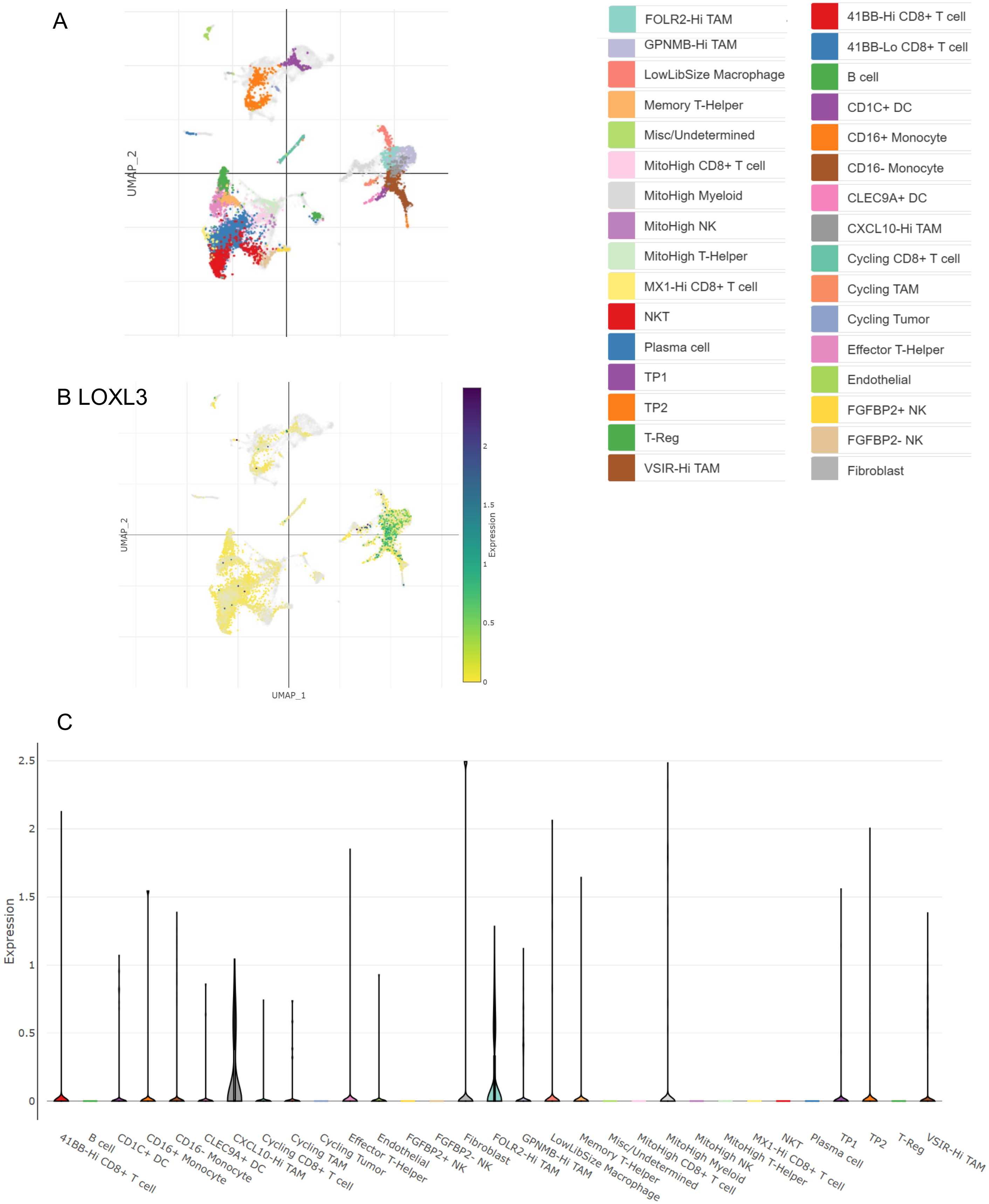

Figure S20. scRNA-seq reveals a distinct distribution of LOXL3 in macrophages of kidney cancer. (A) The UMAP of the study from Kevin Bi et al. was directly obtained from https://singlecell.broadinstitute.org/single_cell (n=8 patients). N=32 clusters were identified. (B) Distribution of *LOXL3* mRNA expression. (C) Violin plots visualization of LOXL3 mRNA expression. Abbreviations: DC, dendritic cell; TAM, tumor-associated macrophage.

Figure S21

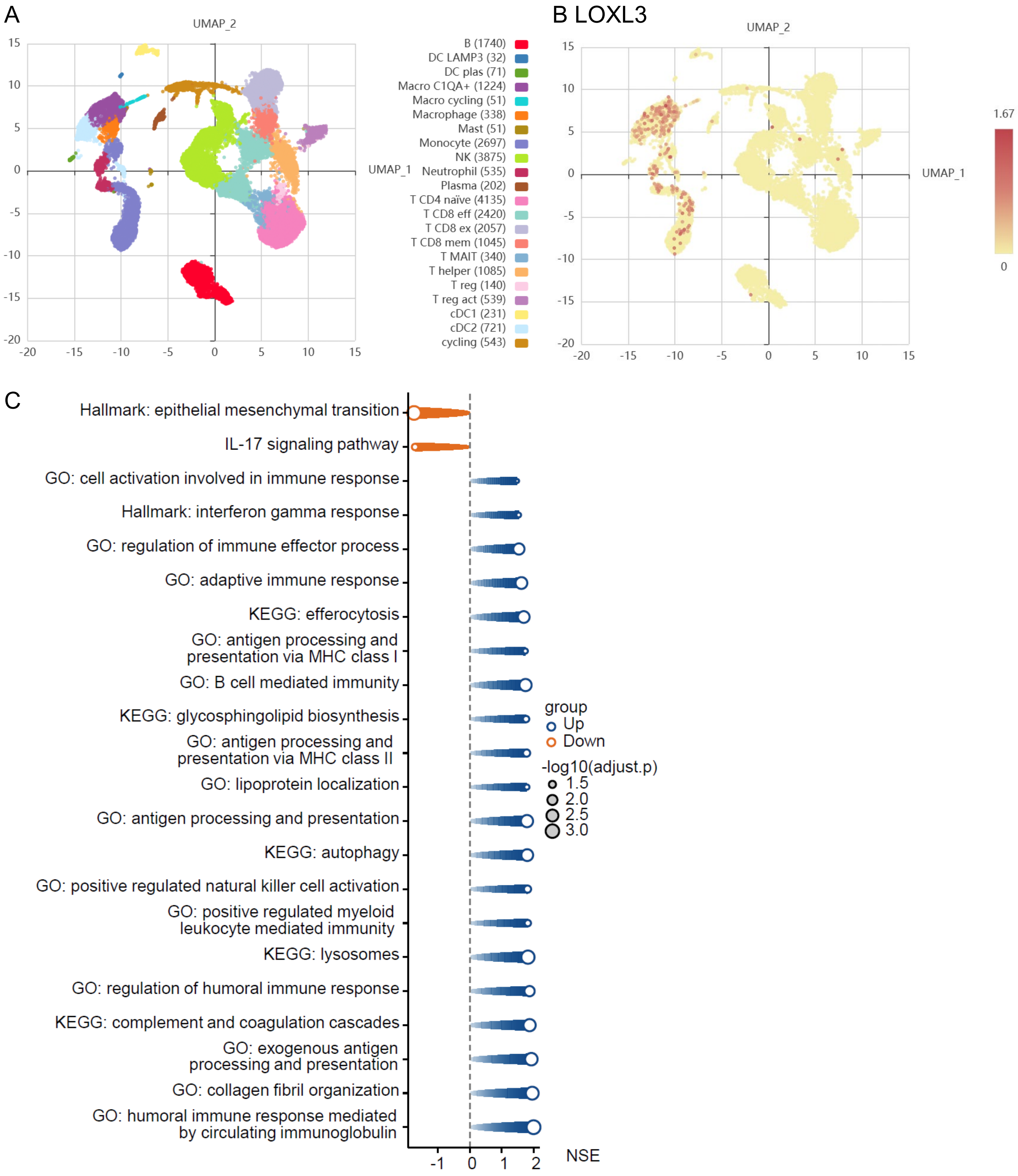

Figure S21. scRNA-seq reveals a distinct distribution of LOXL3 in macrophages of kidney cancer. (A) The UMAP of GSE121638 was directly obtained from https://immucanscdb.vital-it.ch (n=3 patients). N=22 clusters were identified. (B) Distribution of LOXL3 mRNA expression. (C) Pathway enrichment of genes in LOXL3-positive macrophage compared with -negative macrophages. GSEA were performed. Abbreviations: DC, dendritic cell; macro, macrophage; eff, effective; ex, exhausted; mem, memory; act, active.
